# Radical Cyclization-Initiated Difunctionalization Reactions of Alkenes and Alkynes

**DOI:** 10.3390/molecules29112559

**Published:** 2024-05-29

**Authors:** Sanjun Zhi, Xiaoming Ma, Wei Zhang

**Affiliations:** 1 Jiangsu Key Laboratory for the Chemistry of Low-Dimensional Materials, Huaiyin Normal University, 111 Changjiang West Road, Huaian 223300, China; jchyzhi2003@126.com; 2 School of Pharmacy, Changzhou University, 1 Gehu Road, Changzhou 213164, China; mxm.wuxi@cczu.edu.cn; 3 Department of Chemistry, University of Massachusetts Boston, 100 Morrissey Boulevard, Boston, MA 02125, USA

**Keywords:** radical, difunctionalization, atom transfer, cyclization, coupling, addition

## Abstract

Radical reactions are powerful in the synthesis of diverse molecular scaffolds bearing functional groups. In previous review articles, we have presented 1,2-difunctionalizations, remote 1,3-, 1,4-, 1,5-, 1,6- and 1,7-difunctionalizations, and addition followed by cyclization reactions. Presented in this paper is radical cyclization followed by the second functionalization reaction. The second functionalization could be realized by atom transfer reactions, radical or transition metal-assisted coupling reactions, and reactions with neutral molecules, cationic and anionic species.

## 1. Introduction

Feasible radical transformations including addition, cyclization, coupling, atom or group transfer, rearrangement, and fragmentation reactions have made radical reactions a powerful tool for making carbon-carbon and carbon-heteroatom bonds [1,2]. The recent developments in radical cyclative functionalization [3,4], radical-enabled bicyclization [5], photoredox reactions [6,7,8], mechanoredox reactions [9], electrochemical reactions [10], and transition metal-assisted radical reactions [11,12,13,14] have empowered the utility of synthetic radicals. Radical reactions being performed in a cascade sequence for the assembling of complex molecular scaffolds with multiple functional groups in regio- and diastereoselective manners is a unique feature. In addition to the cascade reaction sequence, the final radical intermediates could be trapped by radicals or other active species to introduce new functional groups. This process makes the radical reactions even more attractive. 

**Scheme 1:**
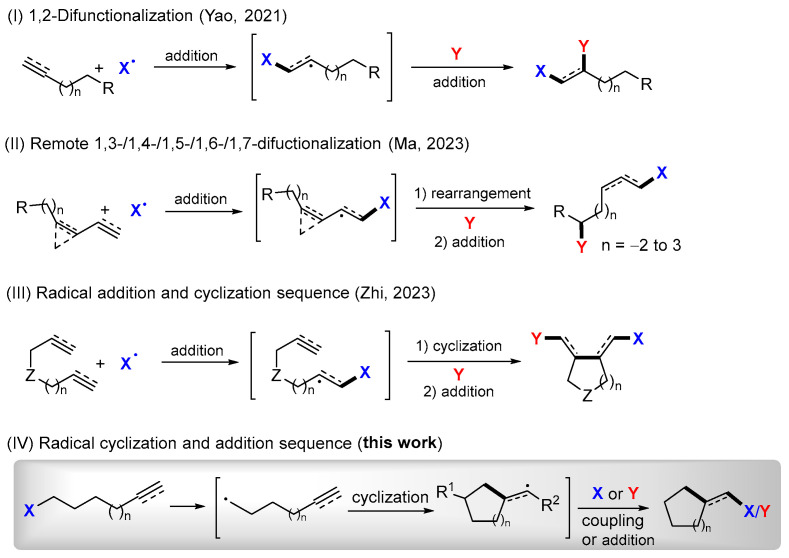
Different kinds of radical difunctionalization reactions [26,27,28].

**Scheme 2:**
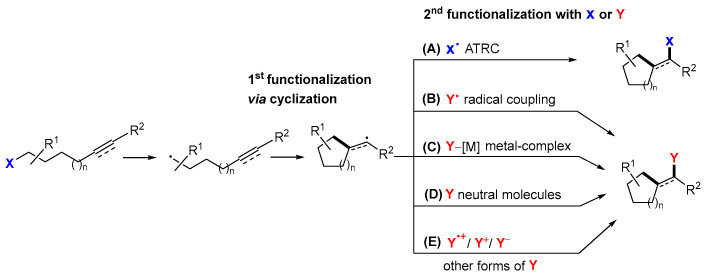
Radical cyclization followed by the 2nd functionalization with **X** or **Y**.

Radical difunctionalization is an attractive topic due to its advantages of synthetic efficiency and product structure diversity. There are a number of reviews on this topic [15,16,17,18,19,20,21,22,23,24,25]. In our recent review articles, we highlighted 1,2-difunctionalization of alkenes and alkynes (Scheme 1, I) [26], remote 1,3-, 1,4-, 1,5-, 1,6- and 1,7-difunctionalization of alkenes and alkynes (Scheme 1, II) [27], and addition/cyclization sequence of dienes, enynes and related compounds (Scheme 1, III) [28]. Presented in this paper are radical cyclization-initiated reactions followed by the second functionalization for the synthesis of cyclic compounds (Scheme 1, IV). It covers the work of the past decade, but most are from the last five years. The second functionalization reaction could be accomplished by atom transfer reactions, radical or transition metal coupling reactions, and reactions involving neutral molecules, cationic and anionic species (Scheme 2). In the reaction process, radical cyclization is considered the first functionalization, while the introduction of X or Y groups is the second functionalization. 

It is noteworthy that the reactions covered in this paper involve only a single cyclization. More sophisticated double or multiple cyclization reactions are not included in this paper. 

## 2. Second Functionalization with X via Atom-Transfer Radical Cyclization 

**Scheme 3:**
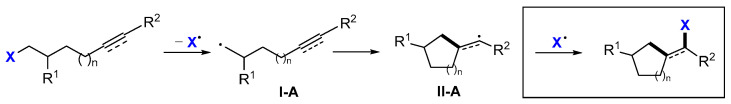
Atom-transfer radical cyclization (ATRC) reaction.

In atom-transfer radical cyclization (ATRC) reactions, radicals **I-A** generated from the homolytic cleavage of X–C bond undergo cyclization to form radicals **II-A** followed by coupling with radical X for the second functionalization to give products (Scheme 3). A majority of radical cyclization-initiated difunctionalization are ATRC reactions. The common X groups could be I, Br and Cl atoms, or carbonate, pyridinyl, xanthyl, and (2,2,6,6-tetramethylpiperidin-1-yl)-oxyl (TEMPO)-related groups. The reactions presented in this section are classified based on the substrates which include halo-alkenes or -alkynes, *N*-allyl-haloacetamides, *N*-allyl-haloamines, *O*-allyl-halo ethers, *O*-allyl-halo- hemiacetal acetates and other related compounds.

### 2.1. Halo Alkenes or Alkynes as Substrates

**Scheme 4:**
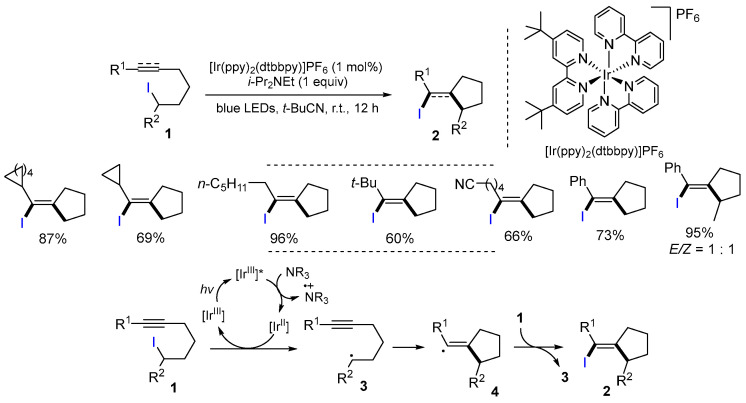
Synthesis of (iodomethylene)cyclopentanes.

The iodine atom is a good transfer group for ATRC. In 2017, Martin and coworkers developed a visible light-promoted ATRC reaction of unactivated alkyl iodides for the synthesis of (iodomethylene)cyclopentanes. The reaction was carried out using alkyl iodides **1** in the presence of [Ir(ppy)_2_(dtbbpy)]PF_6_ and *i*-Pr_2_NEt in *t*-BuCN under blue LEDs irradiation at room temperature for 12 h to give products (iodomethylene)cyclopentanes **2** in good to excellent yields (Scheme 4) [29]. The suggested reaction mechanism indicates that radicals **3** generated via a single electron transfer (SET) process of alkyl iodides **1** with [Ir^II^] undergo a 5-*exo* cyclization to give radicals **4** and then lead to the formation of products **2** after trapping the iodine radical from alkyl iodides **1**. 

**Scheme 5:**
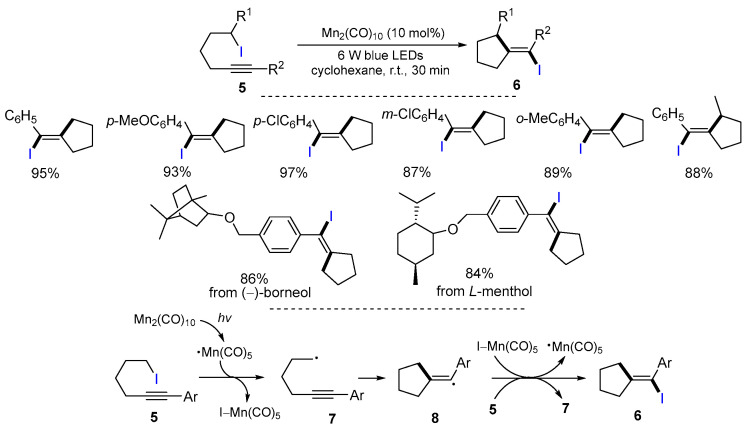
Synthesis of iodocyclic alkenyl iodides.

In 2019, Zhang and coworkers reported a visible-light-induced ATRC reaction of alkyl iodides for the synthesis of cyclic alkenyl iodides (Scheme 5) [30]. The Mn_2_(CO)_10_-catalyzed reaction of alkyl iodides **5** under the irradiation of blue LEDs in cyclohexane at room temperature gave products **6** in moderate to excellent yields. The reactions of natural products such as (−)-borneol and *L*-menthol also afforded the corresponding products in excellent yields. The proposed reaction mechanism suggested that the Mn(CO)_5_ radical is generated by the photo-induced Mn−Mn bond homolysis of Mn_2_(CO)_10_. The reaction of alkyl iodides **5** with the Mn(CO)_5_ radical affords radicals **7** which undergo 5-*exo* cyclization to form radicals **8** followed by iodine atom transfer from **5** to give products **6**.

**Scheme 6:**
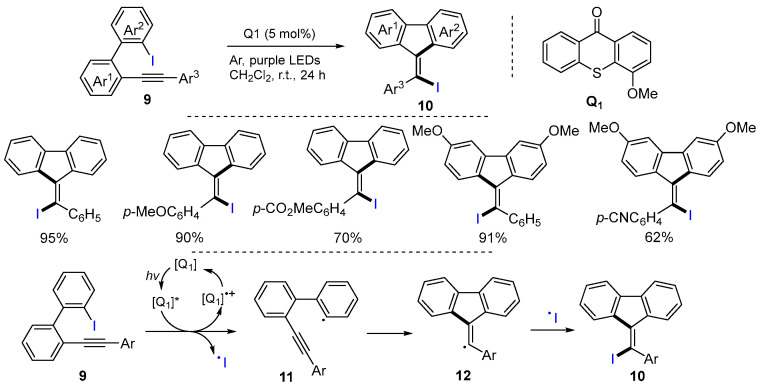
Synthesis of iodo-substituted fluorene derivatives.

Other than iodoalkyl group, iodoaryl groups are also good for ATRC. Guo and coworkers, in 2019, introduced a photo-induced ATRC reaction of aryl iodide for the synthesis of iodine-substituted fluorene derivatives. The reaction of aryl iodide **9** with photosensitizer thioxanthone (**Q_1_**) under the irradiation of purple LEDs in CH_2_Cl_2_ at room temperature gave products **10** in good to excellent yields (Scheme 6) [31]. The reaction process involves the formation of triplet **9** via SET reduction in the photoexcited state [**Q_1_**]^*^ species to [**Q_1_**]^•+^. The Ar–I bond cleavage of triplet **9** produces aryl radicals **11** and the iodine radical. Subsequential radical cyclization to form **12** followed by the iodine radical coupling affords products **10**.

### 2.2. N-Allyl-haloacetamides as Substrates

**Scheme 7:**
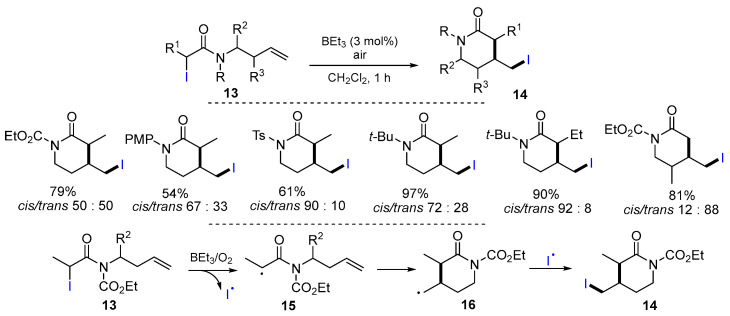
Synthesis of iodinated lactams.

*N*-Allyl-haloacetamides have been used as the substrates for ATRC to make *N*-containing heterocyclic compounds. A BEt_3_/O_2_-initiated iodine-atom-transfer radical cyclization of *N*-(but-3-en-1-yl)-*N*-(*tert*-butyl)-2-iodoalkanamides for the synthesis of iodine substituted *δ*-lactams has been reported by Li, Liu and their coworkers in 2016. The reaction of *N*-(but-3-en-1-yl)-*N*-(*tert*-butyl)-2-iodoalkanamides **13** in the presence of BEt_3_ and air for 1 h afforded products **14** in good yields (Scheme 7) [32]. In this reaction process, radicals **15** formed via the abstraction of iodine atom of **13** by BEt_3_ undergo the 6-*exo* cyclization to give radicals **16** followed by coupling with iodine radical to form products **14**.

**Scheme 8:**
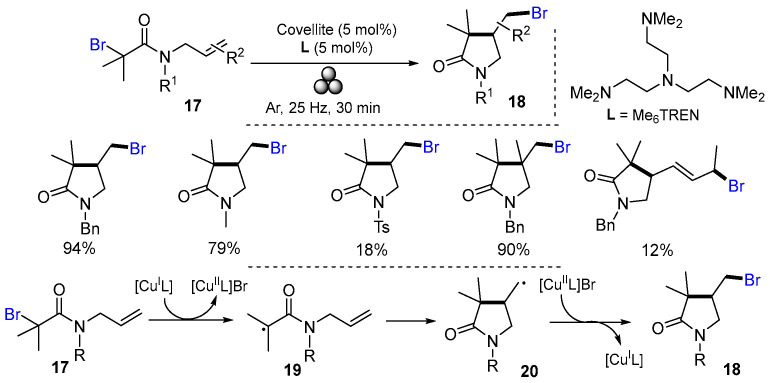
Synthesis of brominated lactams.

In 2020, Bolm and co-workers reported a mechanochemical reaction of *N*-allyl- 2-bromo-propanamides for the synthesis of brominated lactams. The reaction of *N*-allyl-2-bromo-propanamides **17** in the presence of the mineral covellite and tris[2-(dimethyl-amino)ethyl]amine (Me_6_TREN) under mechanochemical conditions in ball mills gave **18** in good to excellent yields (Scheme 8) [33]. The reaction of *N*-allyl-2-bromo-propanamides **17** and [Cu^I^L] generated from covellite and Me_6_TREN afford radicals **19** which undergo 5-*exo* cyclization to give radicals **20** followed by bromide atom transfer from [Cu^II^L]Br to close the catalytic cycle and form products **18**. In another paper, Bolm and co-workers conducted similar reactions but modified the reaction conditions by using Cu(OTf)_2_, tris(2-pyridylmethyl)amine (TPMA) and *tet*-BaTiO_3_ to give brominated lactams in good yields [34].

**Scheme 9:**
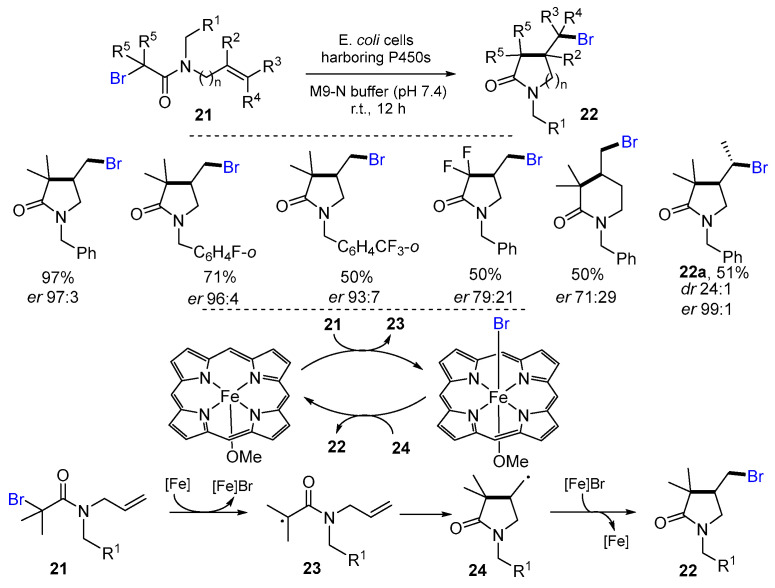
Synthesis of brominated lactams.

In 2021, Yang and coworkers developed cytochromes P450 metalloenzyme-catalyzed radical cyclization reactions of bromo-propanamides for the synthesis of nitrogen heterocycles. The reaction of 2-bromo-propanamides **21** in the presence of whole-cell biotransformation E. *coli* cells resuspended in M9-N buffer (pH 7.4) at room temperature gave products **22** in good yields (Scheme 9) [35]. *γ*-Lactam product **22a** was obtained in a good diastereomeric ratio of 24:1 which is better than the traditional Cu(OTf)_2_/TPMA catalysis of 64:36 diastereomeric ratio [34]. In this metalloenzymatic reaction process, radicals **23** formed by the reaction of *N*-allyl-2-bromo-propanamides **21** and [Fe] undergo 5-*exo* cyclization to give radicals **24** which abstract Br from [Fe]Br to give products **22.**

**Scheme 10:**
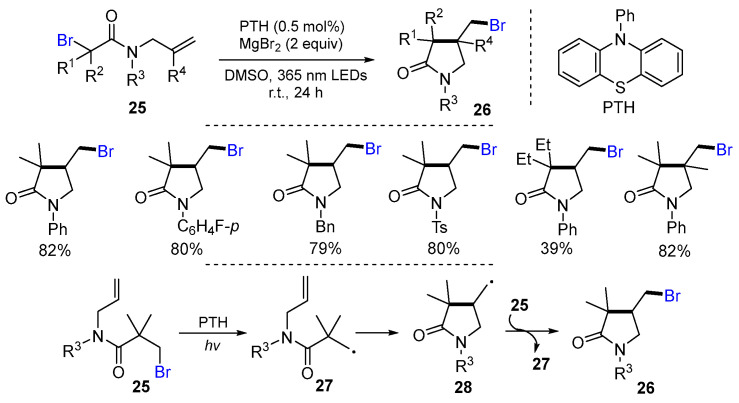
Preparation of brominated *γ*-lactams.

Nishikata and coworkers, in 2021, reported a photo-induced radical reaction of *N*-allyl-*α*-haloamides for the synthesis of *γ*-lactams. The reaction of *N*-allyl-*α*-haloamides **25** in the presence of *N*-Ph-phenothiazine (PTH) and MgBr_2_ in DMSO under the irradiation of 365 nm LEDs at room temperature for 24 h gave products **26** in good to excellent yields (Scheme 10) [36]. The reaction first generates radicals **27** from the reaction of *N*-allyl amide **25** with PTH under the irradiation of LEDs. The cyclization of radicals **27** gives radicals **28** which trap the Br radical from *N*-allyl amides **25** to give products **26**. 

**Scheme 11:**
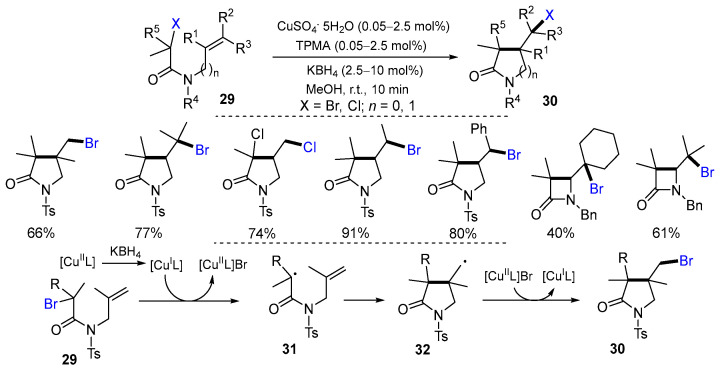
Synthesis of hologenated nitrogen heterocycles.

Clark and coworkers reported a Cu-catalyzed ATRC reaction of halo-olefins for the synthesis of nitrogen heterocycles in 2012. The reaction of *N*-allyl-haloacetamides **29** in the presence of CuSO_4_·5H_2_O, TPMA and borohydride salts at room temperature for 10 min gave products **30** in good yields (Scheme 11) [37]. The proposed mechanism suggested that [Cu^I^L] produced from the reduction in [Cu^II^L] with KBH_4_ reacts with **29** to form radicals **31** followed by the cyclization to give radicals **32**. Final products **30** are obtained from the reaction of radicals **32** and [Cu^II^LBr] along with the regeneration of [Cu^I^L].

**Scheme 12:**
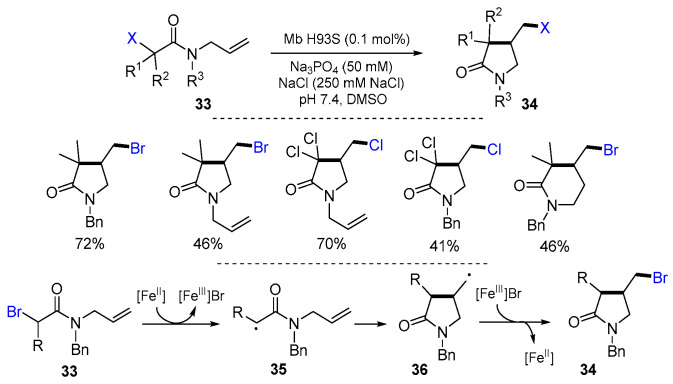
Synthesis of brominated *γ*-lactams.

Pellizzoni and coworkers reported a myoglobin-catalyzed reaction of *N*-allyl-*α*-haloamides for the synthesis of *γ*-lactams in 2022. The reaction of *N*-allyl-*α*-haloamides **33** in the presence of Mb H93S (as purified protein and in whole bacterial cells), sodium ascorbate, and sodium phosphate buffer (pH 7.4) produced products **34** in good yields (Scheme 12) [38]. 

Initial radicals **35** produced by the reaction of *N*-allyl-2-bromo-propanamide **33** and [Fe^II^] of Mb H93S undergo a 5-*exo* cyclization to give radicals **36** followed by coupling with Br radical from [Fe^III^]Br to give products **34**.

**Scheme 13:**
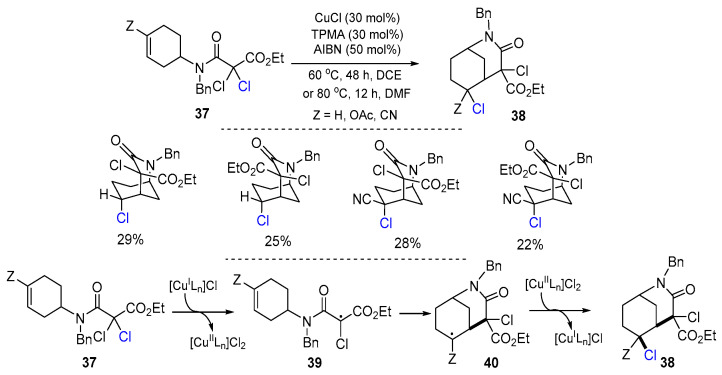
Synthesis of chlorinated nitrogen heterocycles.

Diaba, Bonjoch and coworkers, in 2014, reported a Cu-mediated ATRC reaction of aminotethered dichloromalonamides for the synthesis of 2-azabicyclo[3.3.1]nonanes. The reaction of aminotethered dichloromalonamides **37** (such as carbamoyldichloroacetate-tethered alkenes and *α*,*β*-unsaturated nitriles) in the presence of CuCl, TPMA and AIBN in DCE at 60 °C for 48 h or in DMF at 80 °C for 12 h afforded products **38** in good yields (Scheme 13) [39]. Radicals **39** generated from the reaction of **37** and [Cu^I^L_n_]Cl undergo a 6-*exo* cyclization to give radicals **40** which then trap the Cl radical from [Cu^II^L_n_]Cl_2_ to give products **38** along with the regeneration of [Cu^I^L_n_]Cl.

**Scheme 14:**
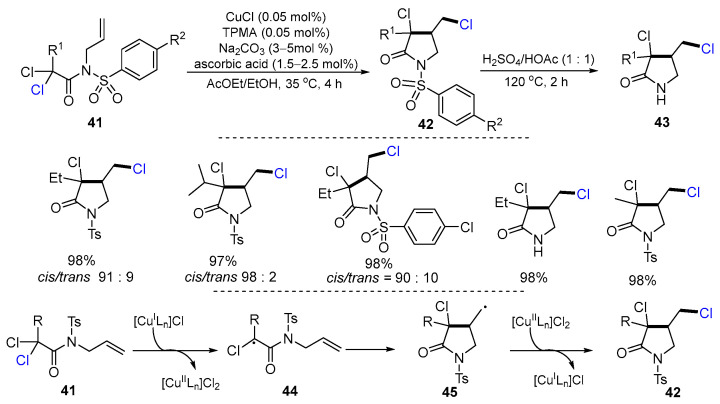
Synthesis of chlorinated nitrogen heterocycles.

In 2014, Ghelfi and coworkers developed a Cu-catalyzed ATRC reaction of dichloromalonamides for the synthesis of nitrogen heterocycles. The reaction of *N*-allyl-*N*-tosyl-2,2-dichlorobutanamides **41** in a mixture of AcOEt/EtOH and in the presence of CuCl/TPMA/ascorbic acid/Na_2_CO_3_ gave *N*-arylsulfonyl-halo-*γ*-lactams **42** in good to excellent yields (Scheme 14) [40]. Next, 3-Pirrolin-2-one **43** could be generated after the deprotection of the *N*-tosyl-halo-*γ*-lactam **42**. The proposed reaction mechanism suggested that radicals **44** generated from the reaction of **41** and [Cu^I^L_n_]Cl undergo a 5-*exo* cyclization to give radicals **45** which then react with [Cu^II^L_n_]Cl_2_ to afford products **42** and [Cu^I^L_n_]Cl.

**Scheme 15:**
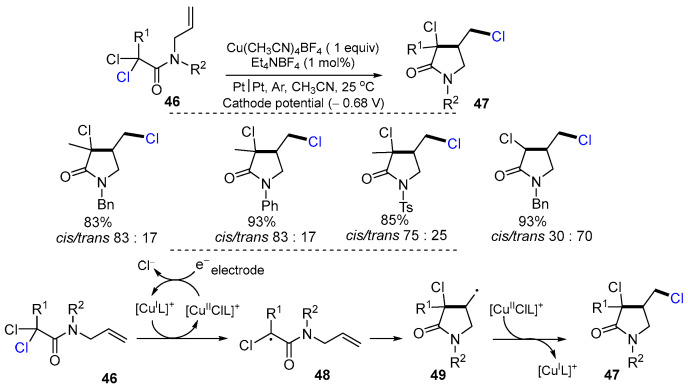
Synthesis of chlorinated nitrogen heterocycles.

In 2015, Isse and Gennaro and their coworkers reported an electrochemical reaction of *N*-allyl-*α*,*α*-dichloroamides for the synthesis of halogenated nitrogen heterocycles. The reaction of *N*-allyl-*α*,*α*-dichloroamides **46** was carried out in a cell assembled with a Pt gauze cathode and a Pt plate anode with the appropriate cathode potential value (often 0.68 V *vs*. SCE). In the presence of Cu(CH_3_CN)_4_BF_4_, TPMA, and Et_4_NBF_4_ in CH_3_CN at 25 °C under argon, the reaction gave products **47** in good to excellent yields (Scheme 15) [41]. In this reaction process, [Cu^I^L]^+^ produced from the reduction in [Cu^II^L]^+^ at the electrode surface reacts with *N*-allyl-*α*,*α*-dichloroamides **46** to form radicals **48** which then undergo cyclization to form radicals **49** followed by the reaction with [ClCu^II^L]^+^ to give products **47**.

**Scheme 16:**
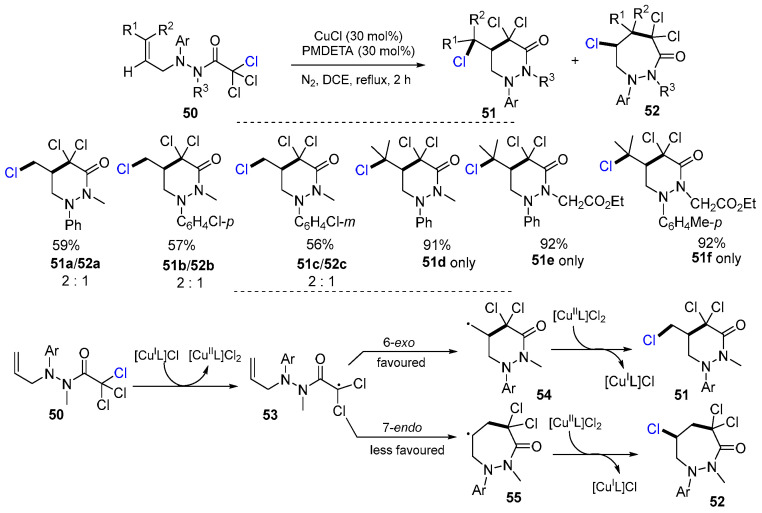
Synthesis of 6- and 7-membered nitrogen heterocycles.

In 2016, Soni and Ram developed Cu-catalyzed radical cyclization reactions of *N*-allyl-*N*″-trichloro-acetylhydrazines for the synthesis of heterocyclic molecules containing two *N*-atoms. The reaction of *N*-allyl-*N*″-trichloroacetylhydrazines **50** in the presence of CuCl and pentamethyldiethylenetriamine (PMDETA) with DCE as solvent at refluxing for 2 h afforded a mixture of chlorinated tetrahydro-pyridazin-3-ones **51** and 1,2-diazepan-3-ones **52** in good yields (Scheme 16) [42]. Two different products were separated based on their solubility in *n*-hexane. The reaction of **50** with [Cu^I^L]Cl first generates dichlorocarbon radical **53** via the abstraction of a chlorine atom by [Cu^I^L]Cl to give [Cu^II^L]Cl_2_. Radicals **53** undergo a more favorable 6-*exo* cyclization to give radicals **54** followed by reaction with [Cu^II^L]Cl_2_ to give 6-membered products **51**. Radicals **53** could also undergo less favorable 7-*endo* cyclization to form radicals **55** and then lead to the formation of 7-membered products **52**. 

### 2.3. N-Allyl-haloamines as Substrates

**Scheme 17:**
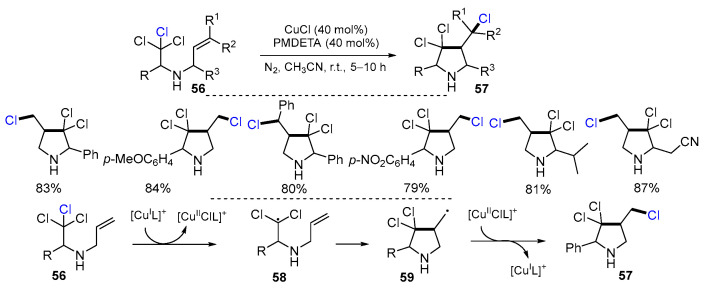
Synthesis of nitrogen heterocycles.

*N*-Allyl-haloamines are another class of substrate for ATRC that originates *N*-containing heterocyclic compounds. A Cu-catalyzed radical cyclization reaction of *N*-allyl-haloamines for the synthesis of substituted 2,4-*trans*-(*N*H)-pyrrolidines was reported by Gupta and coworkers in 2016. The reaction of 2,2,2-trichloro-ethylallyl- *N*H-amines **56** with CuCl and PMDETA with CH_3_CN as solvent at 0 °C gave products **57** in high yields (Scheme 17) [43]. The radicals **58** generated from Cl atom transfer of **56** cyclized to form radicals **59** followed by the reaction with [ClCu^II^L]^+^ to give products **57**.

**Scheme 18:**
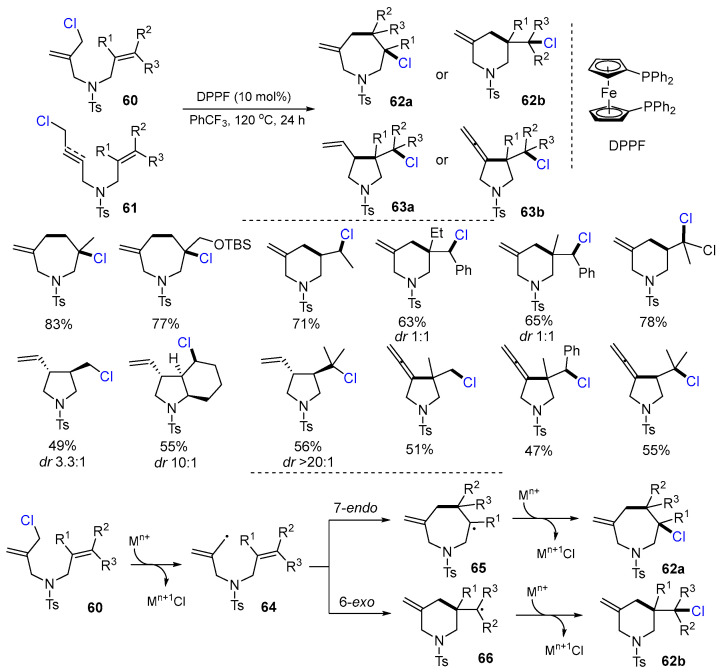
Synthesis of chlorinated heterocycles.

A protocol of Fe-catalyzed radical cyclization reaction of cloromethyl-1,6-dienes /1,6-enynes for the synthesis of nitrogen heterocycles was reported by Tong and coworkers in 2017. The reaction of chloromethyl-1,6-dienes/1,6-enyne **60** or **61** using DPPF [1,1’-bis(diphenylphosphino)-ferrocene] as a catalyst and PhCF_3_ as a solvent at 120 °C for 24 h gave products **62** or **63** in good yields (Scheme 18) [44]. In this reaction process, the abstraction of a chlorine atom from substrates **60** by DPPF leads to the formation of radicals **64** which undergo a 7-*endo* cyclization to form radicals **65**, or a 6-*exo* cyclization to give radicals **66**. The reaction of radicals **65** or **66** with M^n+1^Cl afford products **62a** or **62b**.

**Scheme 19:**
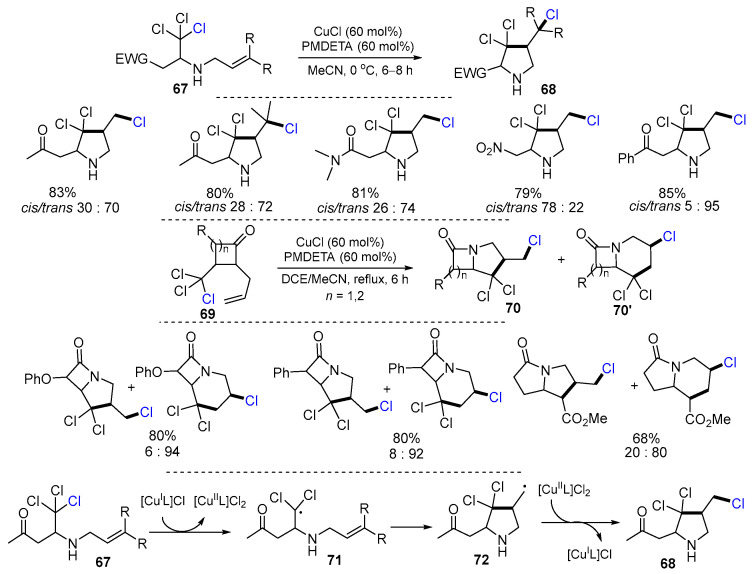
Synthesis of chlorinated heterocycles.

Sadanandan and Gupta, in 2020, developed Cu-catalyzed radical reactions of trichloroethyl allylamines for the synthesis of nitrogen heterocycles. The reaction of trichloroethyl allylamines **67** in the presence of CuCl/PMDETA at 0 °C in MeCN under N_2_ atmosphere gave products **68** in good yields (Scheme 19) [45]. While the reaction of allylamines **69** in refluxing DCE/MeCN under N_2_ atmosphere afforded a mixture of products **70** and **70′**. In the reaction process, radicals **71** formed from **67** via the abstraction of a Cl atom by [Cu^I^L]Cl undergoing a 5-*exo* cyclization to give radicals **72** which then lead to the formation of products **68** after coupling with [Cu^II^L]Cl_2_. 

**Scheme 20:**
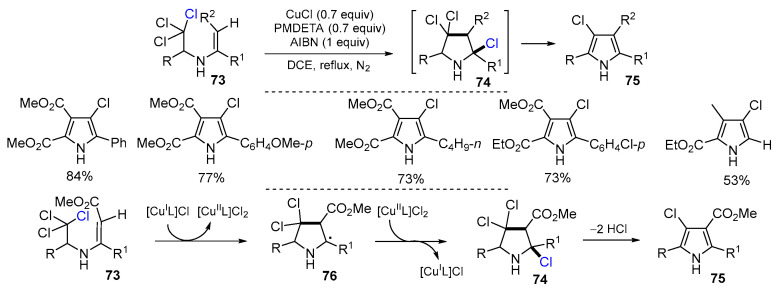
Synthesis of chlorinated tetrahydropyrroles and substituted pyrroles.

In 2019, Gupta and coworkers reported a Cu-catalyzed radical reaction of trichloroethyl-*N*H-enamine for the synthesis of multi-functionalized pyrroles. The reaction of trichloroethyl-*N*H-enamines **73** with CuCl, PMDETA and AIBN in refluxing DCE afforded products **75** in good yields (Scheme 20) [46]. The initial radicals formed via the abstraction of a Cl atom of **73** by [Cu^I^L]Cl undergo a 5-*endo* cyclization to give radicals **76** followed by the Cl atom transfer from [Cu^II^L]Cl_2_ to give products **74.** Sequential dehydrochlorinative aromatization of **74** leads to the substituted pyrroles **75**.

### 2.4. O-Allyl-halo Ethers as Substrates

**Scheme 21:**
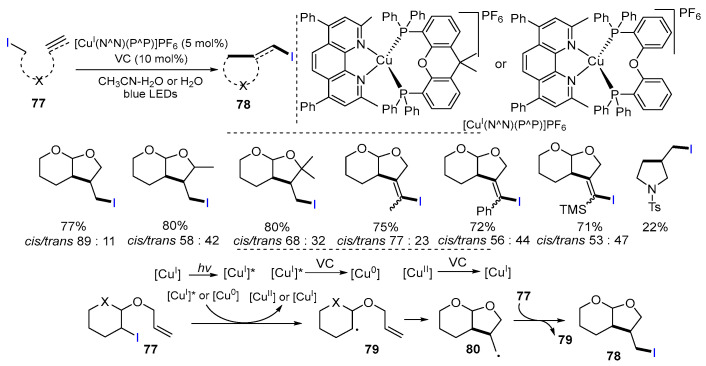
Synthesis of iodine-substituted heterocycles.

There are several examples of using *O*-allyl-halo ethers as starting materials for ATRC to prepare *O*-containing heterocyclic compounds. Yu and Yang [47], in 2022, introduced a Cu-catalyzed radical reaction of unsaturated iodides for the synthesis of iodine-substituted heterocycles. The reaction of 2-allyloxy-3-iodotetrahydropyrans or tetrahydrofurans **77** in the presence of [Cu^I^(N^N)(P^P)]PF_6_ complexes and ascorbic acid (VC) under the irradiation of blue LEDs in a mixture of CH_3_CN and H_2_O or in pure water afforded products **78** in good yields (Scheme 21) [47]. The initial radicals **79** produced from the reaction of **77** under either the photo-excited [Cu^I^]^*^ or the in situ generated [Cu^0^] species from [Cu^I^]^*^ undergo cyclization to form radicals **80** followed by iodine transfer to give products **78**.

**Scheme 22:**
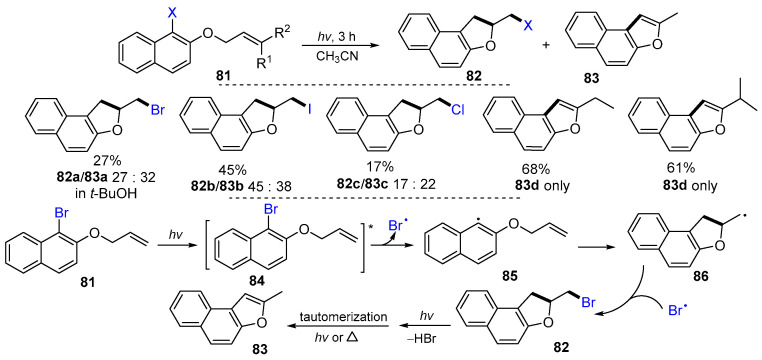
Synthesis of brominated naphtho[*b*]furans.

Yoshimi and co-workers, in 2014, reported a photo-induced radical reaction of allyl-bromonaphthyl ethers for the synthesis of naphtho[*b*]furans. The reaction of allyl-halo-naphthyl ethers **81** in CH_3_CN (or *t*-BuOH) under the irradiation of a 100 W high-pressure mercury lamp with a Pyrex glass filter (>280 nm) at room temperature for 3 h gave 2-halomethyl substituted naphthodihydrofuran **82** and naphtho[*b*]furans **83** in good yields (Scheme 22) [48]. The reaction involves the formation of triplet states of 84* from **81** under photo-irradiation followed by homolytic cleavage of C–X bond to give radicals **85** and Br radical. The 5-*exo* cyclization of radicals **85** gives radicals **86**, followed by Br radical trapping to yield brominated products **82** which could undergo further dehydrobromination and tautomerization to give **83**.

**Scheme 23:**
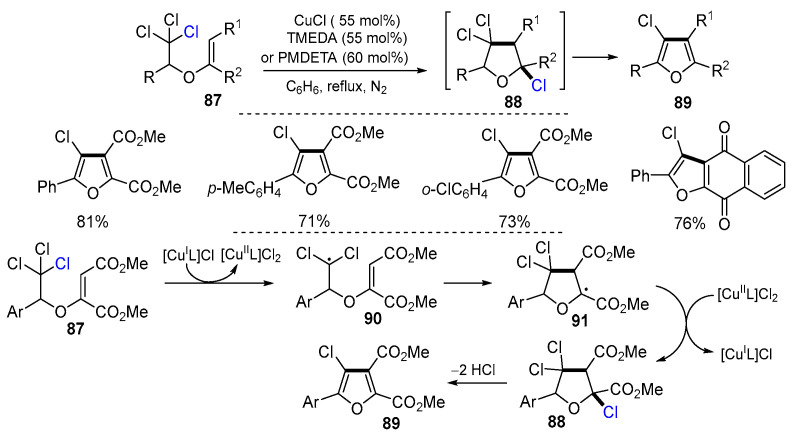
Synthesis of substituted tetrahydrofurans and furans.

In 2016, Soni and coworkers reported a Cu-catalyzed cyclization of 2,2,2-trichloroethyl vinyl ethers for the synthesis of highly substituted 2,3-difunctionalized-4-chlorofurans. The reaction of 2,2,2-trichloroethyl vinyl ethers **87** in the presence of CuCl, and tetramethylethylenediamine (TMEDA) or PMDETA using benzene as solvent at reflux for 12–32 h gave products **89** in good yields (Scheme 23) [49]. Radicals **90**, generated from 2,2,2-trichloroethyl vinyl ethers **87** via abstraction of a chlorine atom by [Cu^I^L]Cl, undergo 5-*endo* cyclization to give radicals **91** which then lead to the formation of products **88** after trapping the Cl radical from [Cu^II^L]Cl_2_. Further transformation of **88** via aromative dehydrochlorination gives substituted furans **89**.

**Scheme 24:**
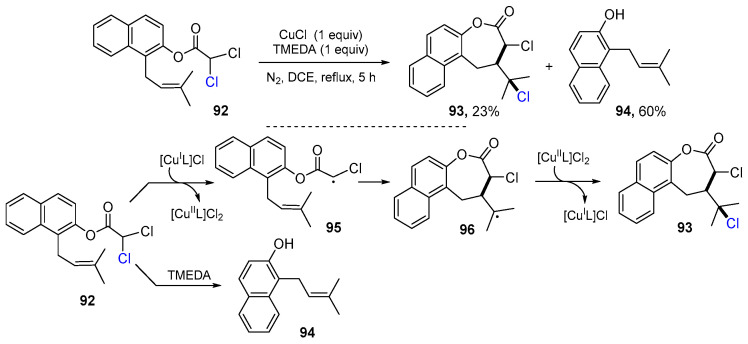
Synthesis of chlorinated 7-membered lactone.

In 2018, the Tittal group reported a Cu-catalysed radical reaction of 1-(3-methyl-but-2-enyl)-naphthalen-2-yl ester for the synthesis of 7-membered lactones. The reaction of 1-(3-methyl-but-2-enyl)-naphthalen-2-yl ester **92** in the presence of CuCl/TMEDA in refluxing DCE for 5 h gave product **93** in 23% yield along with 1-(3-methyl-but-2-enyl)-naphthalen-2-ol **94** in 60 % yield (Scheme 24) [50]. Radical **95,** generated from the homolytic C–Cl bond cleavage of **92,** undergoes 7-*exo* cyclization to give radical **96,** which then traps the Cl radical from [Cu^II^L]Cl_2_ to give product **93**.

### 2.5. O-Allyl-halo-hemiacetal Acetates as Substrates

**Scheme 25:**
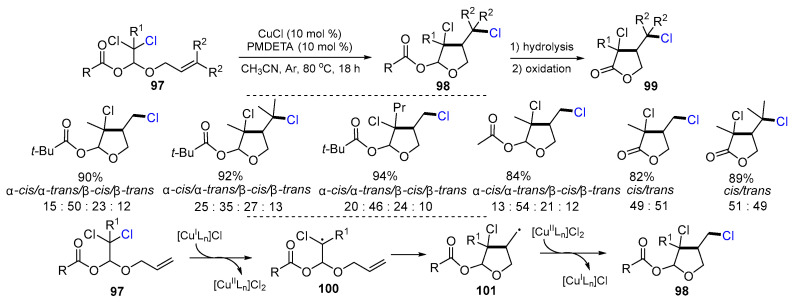
Synthesis of chlorinated cyclic acetals and *γ*-lactones.

The use of *O*- or *S*-allyl-halo-hemiacetal acetates as substrates for ATRC could lead to the formation of cyclic hemiacetal compounds. In 2014, Roncaglia and coworkers introduced a Cu-catalyzed reaction of *O*-allyl-2,2-dichlorohemiacetal acetates for the construction of cyclic acetals and *γ*-lactones. The reaction of *O*-allyl-2,2-dichlorohemiacetal acetates **97** with CuCl and PMDETA in toluene at 80 °C for 18 h afforded cyclic acetals **98** in good to excellent yields (Scheme 25) [51]. The initial radicals **100** generated from the reaction of **97** and [Cu^I^L_n_]Cl undergo cyclization to give radicals **101** which couple with the Cl radical from [Cu^II^L_n_]Cl_2_ to yield cyclic acetals **98**. The cyclic acetals **98** could be readily hydrolyzed and oxidized to form *γ*-lactones **99**.

**Scheme 26:**
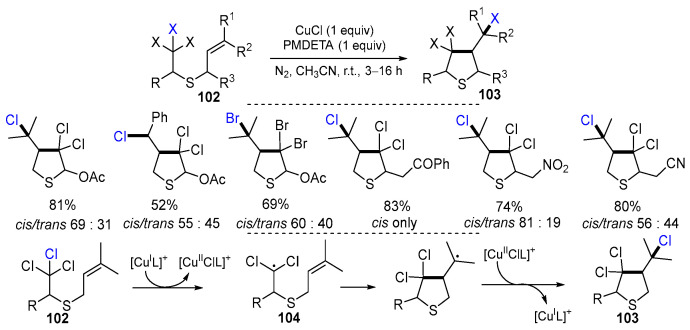
Synthesis of tetrahydrothiophenes.

In 2016, Gupta, Soni and their coworkers reported a Cu-promoted radical reaction of 2,2,2-trihaloethylallyl sulfides for the synthesis of highly substituted tetrahydrothiophenes. The reaction of 2,2,2-trihaloethylallyl sulfides **102** with CuCl/PMDETA in CH_3_CN at room temperature gave products **103** in good to high yields (Scheme 26) [52]. Initial radicals **104** generated from the reaction of 2,2,2-trihaloethylallyl sulfides **102** with [Cu^I^L]^+^ undergo 5-*exo* cyclization followed by the reaction with [Cu^II^ClL]^+^ to give products **103**.

**Scheme 27:**
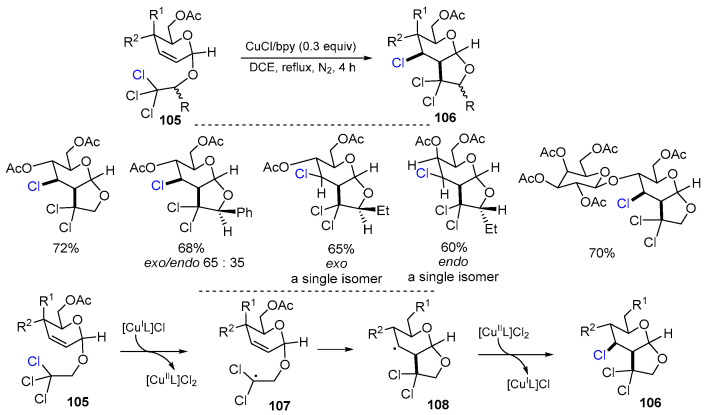
Synthesis of chlorinated perhydrofuro[2,3-*b*]pyrans.

In 2017, Gupta and coworkers reported a Cu-catalyzed radical cyclization reaction of unsaturated carbohydrate-derived chloroacetals for the synthesis of chlorinated perhydrofuro[2,3-*b*]pyrans. The reaction of chloroacetals **105** with CuCl/2,2’-bipyridine in refluxing dichloroehtane (DCE) under N_2_ atmosphere for 4 h gave products **106** in good yields (Scheme 27) [53]. The initial radicals **107** generated from **105** via abstraction of a chlorine atom by [Cu^I^L]Cl undergo 5-*endo*-cyclization to give radicals **108** which then react with [Cu^II^L]Cl_2_ to form products **106**.

### 2.6. Other Vinyl Derivatives as Substrates

**Scheme 28:**
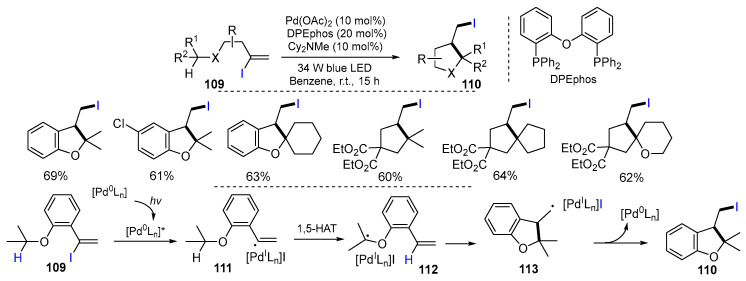
Synthesis of cyclic compounds.

Presented in this section are the ATRC reactions using the substrates which are not included in the previous sections. The transfer groups X could be unique carbonate, pyridinyl, xanthyl, and TEMPO-related groups. Similar to the halogen atoms, these groups are good for homolytic cleavage to generate radicals for ATRC reactions. Gevorgyan and coworkers in 2018 reported a Pd-catalyzed radical reaction of iodovinyl derivatives for the synthesis of cyclic compounds. The reaction of iodovinyl derivatives **109** in the presence of Pd(OAc)_2_, DPEphos and Cy_2_NMe under the irradiation of 34 W blue LEDs in benzene afforded products **110** in good to excellent yields (Scheme 28) [54]. The reaction first generates Pd-radical intermediates **111** via a SET process of vinyl iodides **109** with the active [Pd^0^L_n_]^*^. After 1,5-HAT (hydrogen atom transfer) of **111** to form **112** followed by a 5-*exo* cyclization give radicals **113**, these undergo reductive elimination of [Pd^0^L_n_] to give products **110**.

**Scheme 29:**
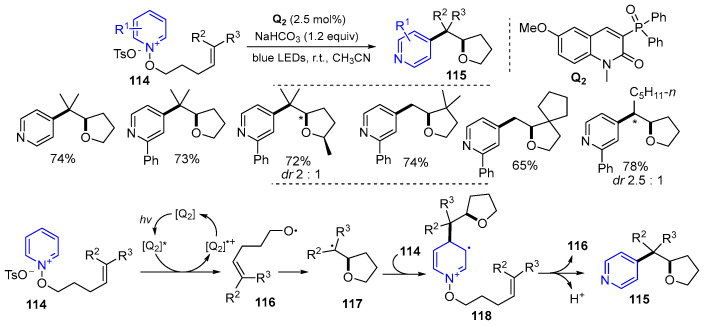
Synthesis of pyridine-tethered tetrahydrofurans.

A unique ATRC reaction involving the pyridinyl group was reported by Hong and coworkers in 2019. They developed a visible-light-induced radical reaction of *N*-alkenyloxypyridinium salts for the synthesis of pyridine-tethered tetrahydrofurans. The reaction of *N*-alkenyloxypyridinium salts **114** in the presence of 3-phosphonated quinolinone (**Q2**) and NaHCO_3_ under the irradiation of blue LEDs at room temperature gave products **115** in good yields (Scheme 29) [55].

**Scheme 30:**
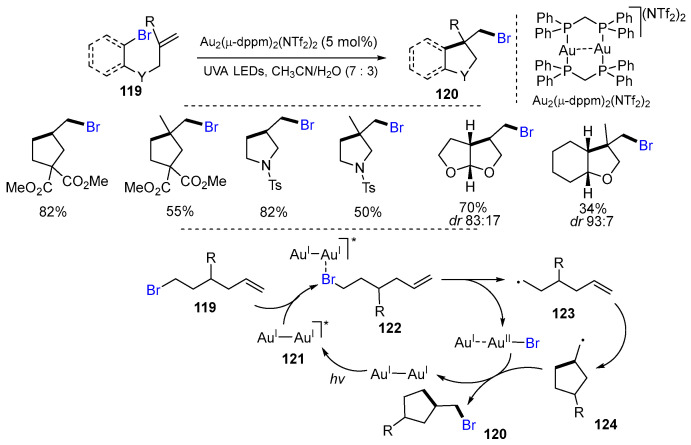
Synthesis of brominated cyclic compounds.

The initial alkoxy radicals **116** generated from *N*-alkoxypyridinium salts **114** via the reaction with photoexcited state [**Q_2_**]^*^ undergo 5-*exo* cyclization to give radicals **117**. Reaction of **117** with substrates **114** for the addition of pyridyl group forms radicals **118** followed by the N–O bond cleavage to give products **115**. In 2020, Barriault and coworkers reported a photo-induced radical reaction of bromoalkanes for the synthesis of cyclic compounds. The reaction of unactivated bromoalkanes **119** in the presence of Au_2_(*μ*-dppm)_2_(NTf_2_)_2_ under the irradiation of UVA LEDs in CH_3_CN/H_2_O produced products **120** in good yields (Scheme 30) [56]. In the reaction process, bromoalkenes **119** react with [Au^I^−Au^I^]^*^ species **121** to form intermediates **122** followed by C–Br bond cleavage to give radicals **123** and 5-*exo* cyclization to form radicals **124**. In the end, the products **120** are obtained from radicals **124** after Br atom abstraction. 

**Scheme 31:**
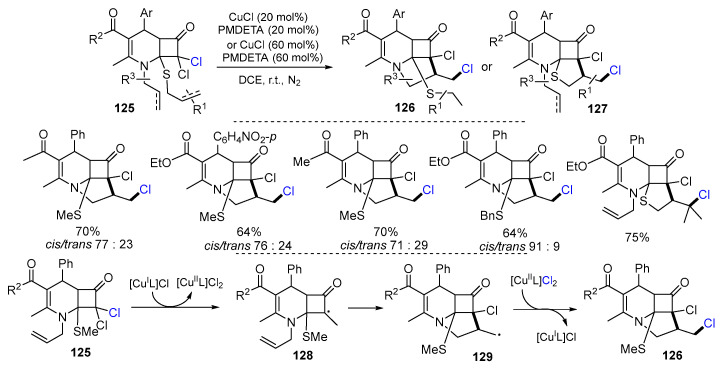
Synthesis of tricyclic *β*-lactams.

A Cu-catalyzed radical reaction of allyl bicyclic *β*-lactam for the synthesis of tricyclic *β*-lactams has been reported by Dawra and coworkers in 2020. The reaction of allyl bicyclic *β*-lactam **125** under the catalysis of CuCl/PMDETA in DCE at room temperature gave products **126** or **127** in good to excellent yields (Scheme 31) [57]. Between two alkenyl groups (*S*-allyl and *N*-allyl) the competitive experiments revealed that the ATRC is more favorable to the *S*-allyl group than the *N*-allyl group. It was suggested that radicals **128** formed via the abstraction of a Cl atom of **125** by [Cu^I^L]Cl undergo a 5-*exo* cyclization to give radicals **129** which then react with [Cu^II^L]Cl_2_ to give products **126**. 

**Scheme 32:**
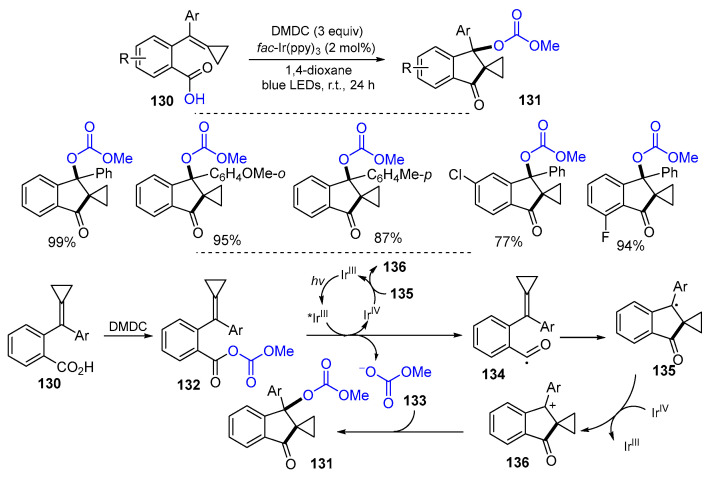
Synthesis of methyl carbonated spiro[cyclopropane-1,2-indan]ones.

Shi and coworkers in 2021 introduced an interesting ATRC reaction involving carbonate radical transformation. The photo-induced radical reaction of methylenecyclopropanes tethered with carboxylic acid for the synthesis of spiro[cyclopropane- 1,2-indan]ones. The reaction of carboxylic acids tethered methylenecyclopropanes **130** and dimethyl dicarbonate (DMDC) in the presence of *fac*-Ir(ppy)_3_ under 5 w blue LEDs in 1,4-dioxane at room temperature gave products **131** in excellent yields (Scheme 32) [58]. In the reaction process, carbonates **132** generated in situ from the reaction of carboxylic acid **130** and DMDC first form radical anions that undergo fragmentation of methyl carbonate **133** to give acyl radicals **134** followed by a 5-*exo* cyclization to form radicals **135**. Sequential oxidation of **135** to cations **136** and nucleophilic attack by cation **133** produces products **131**. 

**Scheme 33:**
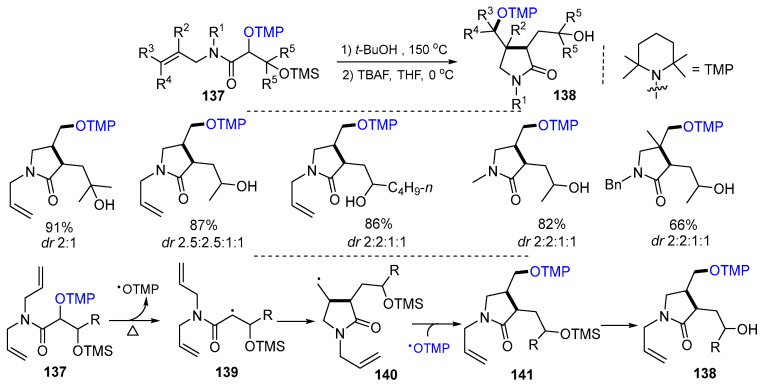
Synthesis of OTMP-functionalized *γ*-lactams.

Jahn and co-workers reported a TEMPO-related ATRC of *α,γ*-dioxygenated amides for the synthesis of *γ*-lactams. The reaction of *α,γ*-dioxygenated amides **137** in *t*-BuOH under the irradiation of microwave at 150 °C for 1 h followed by the treatment with TBAF in THF gave products **138** in good to excellent yields (Scheme 33) [59]. The proposed reaction mechanism suggests that radicals **139** generated from *N*-allyl amides **137** undergo a 5-*exo* cyclization to give radicals **140** which then couple with OTMP radical to form **141** and then products **138** after OTMS deprotection.

**Scheme 34:**
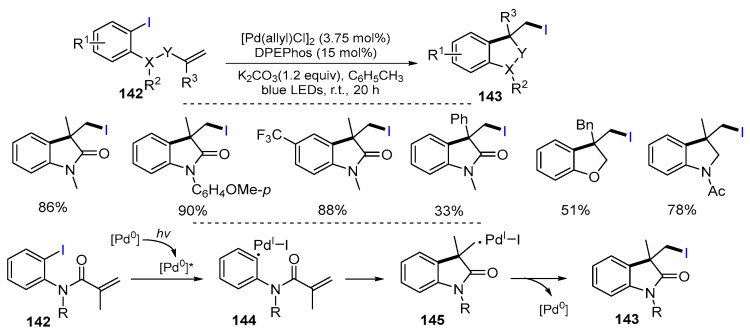
Synthesis of nitrogen heterocycles.

Marchese and coworkers, in 2022, developed a photo-induced radical reaction of allyl 2-iodobenzenes for the synthesis of iodinated heterocyclic compounds. The reaction of allyl 2-iodobenzenes **142** in the presence of [Pd(allyl)Cl]_2_, DPEPhos and K_2_CO_3_ in toluene under the irradiation of blue LEDs gave products **143** in good yields (Scheme 34) [60]. The initial radicals **144** produced by photo reaction of **142** with [Pd^0^]^*^ undergo a 5-*exo* cyclization to form **145** followed by iodine transfer to give product **143**.

**Scheme 35:**
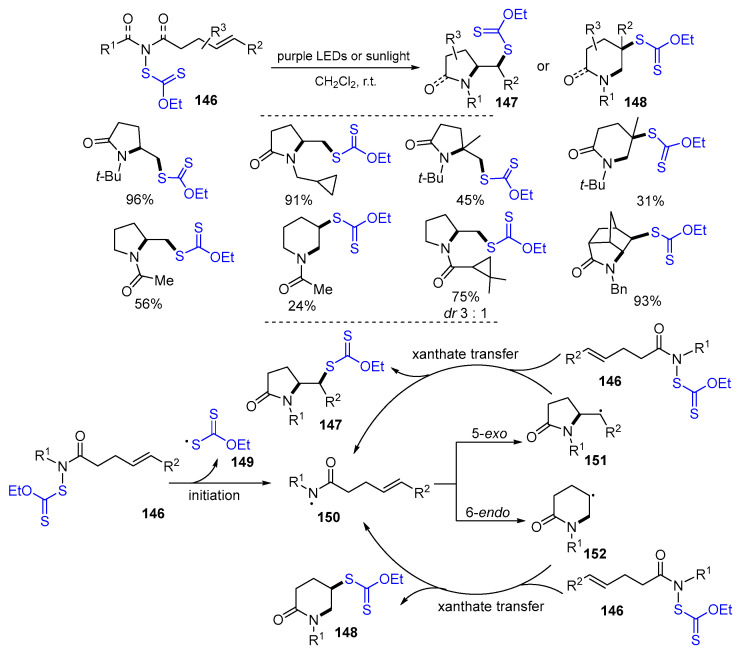
Synthesis of nitrogen heterocycles.

Chen and Wang and coworkers, in 2022, introduced a xanthate-involved ATRC reaction of unactivated olefins for the synthesis of various nitrogen heterocycles such as *γ*-lactams, *δ*-lactams, pyrrolidines, indolones, quinolinones, and fused polycyclic compounds. The reaction of *N*-xanthylamides **146** in CH_2_Cl_2_ under the irradiation of 24 W 400 nm LEDs at room temperature gave products **147** or **148** in good to excellent yields (Scheme 35) [61]. It is worth mentioning that the xanthate-transfer reaction proceeded without photo-catalyst and additive. The proposed reaction mechanism suggested that visible light-induced N−S bond homolysis of substrates **146** gives xanthate radical **149** and amidyl radicals **150**. The 5-*exo* or 6-*endo* cyclization of **150** gives radicals **151** and **152**, respectively. Finally, the coupling of **151** or **152** with xanthates **146** affords products **147** or **148**.

**Scheme 36:**
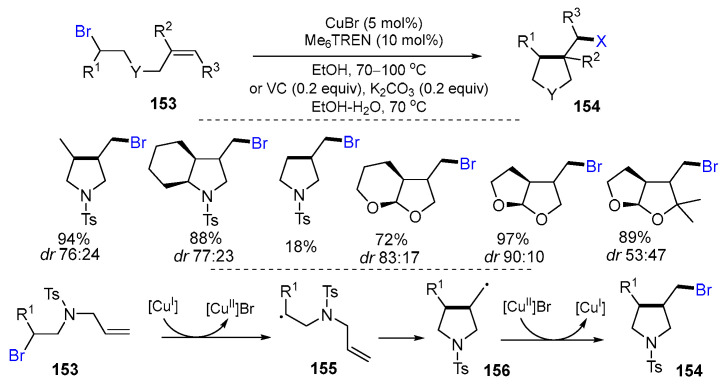
Synthesis of heterocycles.

Yu and coworkers developed a Cu-catalyzed radical reaction of unactivated alkyl bromides for the synthesis of five-membered heterocyclic rings in 2023. The reaction of unactivated alkyl bromides **153** in the presence of CuBr and Me_6_TREN gave products **154** in good to excellent yields (Scheme 36) [62]. It was suggested that radicals **155** produced from the reaction of alkyl bromides **153** and [Cu^I^] undergo a 5-*exo* cyclization to give radicals **156** that then lead to the formation of products **154** after coupling with [Cu^II^]Br.

## 3. Second Functionalization with Y via Radical Coupling 

**Scheme 37:**
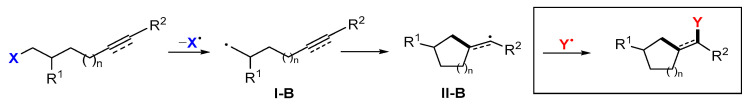
The second functionalization via radical coupling.

Presented in this section are the reactions in which the second functionalization is accomplished by coupling with radical Y (Scheme 37). Different from the ATRC in which the X radical is from the same substrate for cyclization, the Y radical is generated from a different substrate. As shown in Scheme 37, radicals I-B generated from the homolytic cleavage of X–C bond undergo cyclization to form radicals II-B followed by coupling with radical Y generated from a different reactant to give the products. The reactions presented in this section are classified based on the substrates including alkenyl oximes, amino-substituted alkenes, alkynes, and allenes, halo-substituted alkenes, alkynes and allenes as well as other alkenes and allenes. There is a wide range of Y radicals that could be used for the second functionalization. The Y groups presented in this section include halogen atoms and NO, CF_3_, Bpin, 2-azaallyl, lauroyl, alkenyl, N_3_, arylthiomethyl, ketyl, and TEMPO-related groups.

### 3.1. Alkenyl Oximes as Substrates

**Scheme 38:**
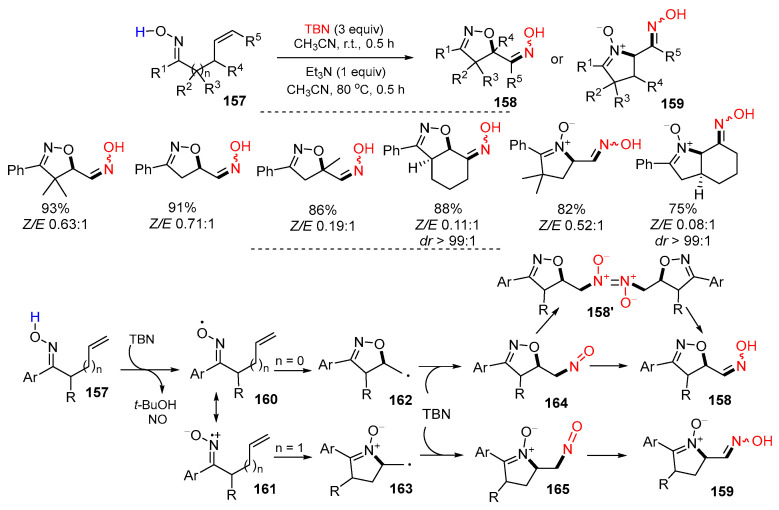
Synthesis of heterocyclic compounds.

There are several examples using alkenyl oximes as substrates for the generation of iminoxyl radicals for cyclization and sequential difunctionalization reactions. Han and coworkers, in 2014, reported a radical cyclization reaction of unsaturated ketoximes for the synthesis of heterocyclic compounds. The reaction of ketoximes **157** in the presence of *tert*-butyl nitrite (TBN) in CH_3_CN at room temperature for 0.5 h, followed by the addition of Et_3_N and heating at 80 °C for 0.5 h afforded oxime featured 4,5-dihydroisoxazoles **158** or cyclic nitrones **159** in good to excellent yields (Scheme 38) [63]. In this reaction, TBN is used as the iminoxyl radical initiator and the carbon radical trap. Initially, the reaction of ketoximes **157** with TBN generates unsaturated iminoxyl radicals (*O*-atom radical **160** and *N*-atom radical **161**) which undergo 5-*exo* cyclization to form radicals **162** (n = 0) and **163** (n = 1), respectively. Radical trapping of **162** or **163** with TBN affords intermediates **164** or **165** which could be further converted to products **158** or **159**. The dimerized **158’** generated from intermediates **164** was isolated and its structure was confirmed by a single-crystal X-ray diffraction study.

**Scheme 39:**
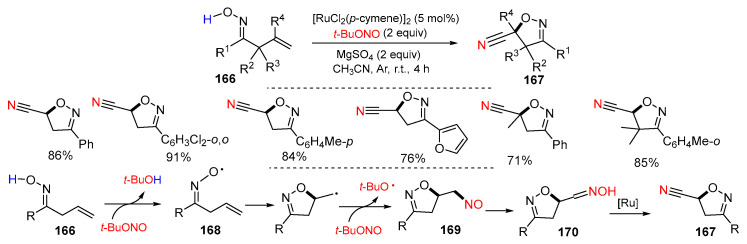
Synthesis of 5-cyanated isoxazolines.

Zhang and coworkers, in 2018, reported a Ru-catalyzed radical reaction of alkenyl oximes for the synthesis of 5-cyanated isoxazolines. The reaction of alkenyl oximes **166** and TBN catalyzed with RuCl_2_(*p*-cymene)]_2_ and MgSO_4_ in CH_3_CN at room temperature for 4 h gave 5-cyanated isoxazolines **167** in good to high yields (Scheme 39) [64]. In the reaction process, TBN acts as an oxidant and a nitrogen source to avoid the use of toxic radical initiators or cyanide reagents. The initial oxime radicals **168** produced via oxidization of alkenyl oximes **166** with TBN undergo 5-*exo* cyclization followed by the coupling with *t*-BuO radical to give intermediates **169** which could be tautomerized to form intermediates **170**. Ru-Catalyzed reduction in **170** gives final products **167**.

**Scheme 40:**
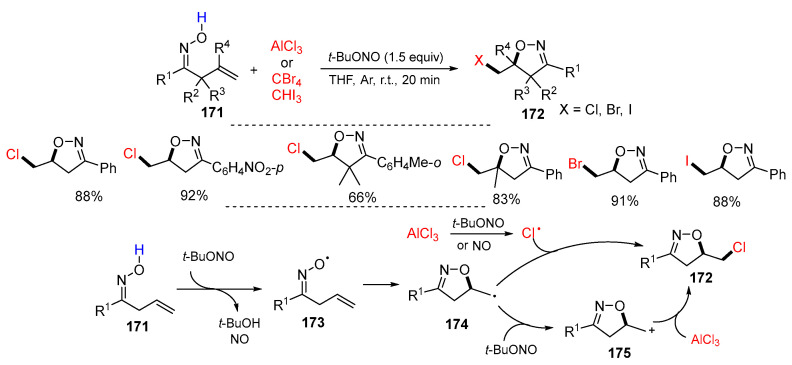
Synthesis of halo-isoxazolines.

In 2016, Kang and coworkers introduced a similar radical reaction of alkenyl oximes for the synthesis of halo-isoxazolines. The reaction of alkenyl oximes **171** with TBN as a dual oxidant and AlCl_3_, CBr_4_ or CHI_3_ as a halogen source in THF/H_2_O for 20 min gave halo-isoxazolines **172** in moderate to good yields (Scheme 40) [65]. In this reaction, the oximes **171** were oxidized by TBN to yield the iminoxyl radicals **173** with the generation of NO and *tert*-butanol, followed by 5-*exo* cyclization to give the radical intermediates **174**. Simultaneously, TBN oxidizes the Cl-anion to the chlorine radical. Next, the final products **172** were obtained from radicals **174** by combining them with the chlorine radical. Otherwise, the cations **175** were formed by oxidization of TBN, followed by reaction with Cl-anion to access the products **172**. 

**Scheme 41:**
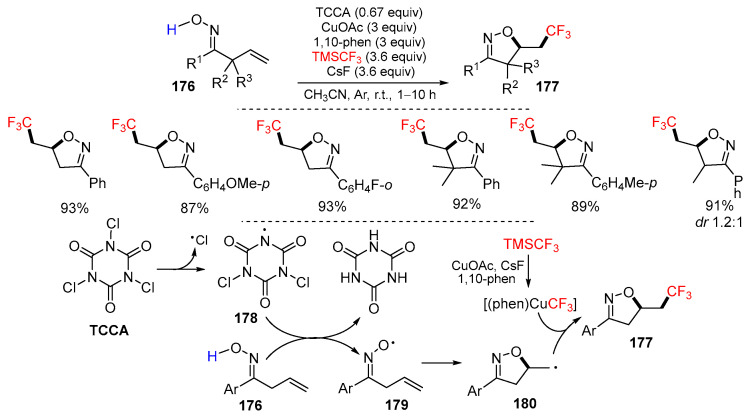
Synthesis of trifluoromethylated isoxazolines.

In 2017, Hu and coworkers developed Cu-catalyzed radical reaction of allylic oximes for the synthesis of trifluoromethylated isoxazolines. The reaction of allylic oximes **176** and TMSCF_3_ in the presence of trichloroisocyanuric acid (TCCA), CuOAc, 1,10-phenanthroline (1,10-phen) and CsF in CH_3_CN at room temperature for 1–10 h afforded products **177** in good to excellent yields (Scheme 41) [66]. In the reaction process, radicals **178** generated from TCCA abstract H atom from allylic oximes **176** to form radicals **179** which undergo 5-*exo* cyclization to give radical **180**. The trapping of CF_3_ radical derived from TMSCF_3_ with **180** generates trifluoromethylated isoxazolines **177**.

**Scheme 42:**
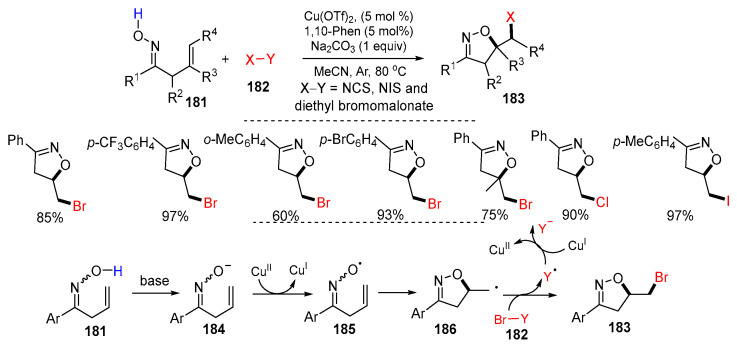
Synthesis of isoxazoline derivatives.

In 2019, Xu and Li and coworkers reported a Cu-catalyzed radical reaction of unsaturated ketoximes for the synthesis of 5-halomethyl isoxazolines. The reaction of ketoximes **181** and halo reagents **182** (such as diethyl bromomalonate, *N*-chlorosuccinimide, and *N*-iodosuccinimide) in the presence of Cu(OTf)_2_, 1,10-Phen and Na_2_CO_3_ in CH_3_CN at 80 °C for 0.5 h produced 5-halomethyl isoxazolines **183** in good to excellent yields (Scheme 42) [67]. The iminoxyl anions **184** formed by the deprotonation of ketoximes **181** with a base undergo SET with Cu^II^ to generate iminoxyl radicals **185**. Subsequent radical cyclization of **185** to form **186** followed by halogen atom transfer with Br–Y **182** originates the desired products **183**. 

### 3.2. Amino-Substituted Alkenes, Alkynes, and Allenes as Substrates

**Scheme 43:**
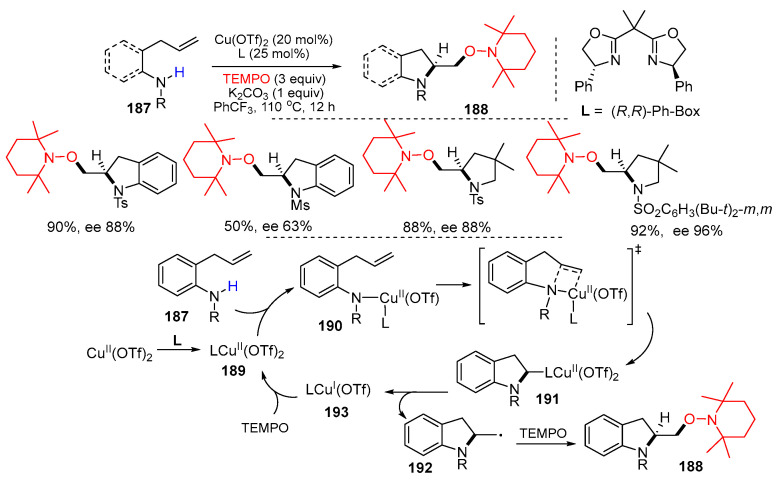
Synthesis of TEMPO-functionalized chiral indolines and pyrrolidines.

Amino-substituted alkenes, alkynes, and allenes could be used as the substrates to generate *N*-radicals for cyclative difunctionalization reactions. In 2013, Chemler and coworkers introduced a Cu-catalyzed enantioselective radical reaction of alkenyl sulfonamides for the synthesis of chiral indolines and pyrrolidines. The aminooxygenation reaction of alkenyl sulfonamides **187** with the use of TEMPO as the oxygen source and under the catalysis of Cu(OTf)_2_ and (*R*,*R*)-Ph-Box in PhCF_3_ gave desired products **188** in good to excellent yields and high enantioselectivity (Scheme 43) [68]. The reaction kinetics showed a first-order dependence in the sulfonamide substrate and the Cu−bis(oxazoline) complex and zero order in TEMPO. In the reaction process, (*R*,*R*)-Ph-Box)/Cu(OTf)_2_ complex **189** reacts with **187** to produce N−Cu^II^ intermediates **190** which then leads to the formation of **191**. Homolysis of **191** to give **192** followed by trapping with TEMPO affords products **188**. The reactive Cu^II^ species **189** is regenerated by oxidization of the Cu^I^ species **193** with TEMPO to complete the catalytic cycle.

**Scheme 44:**
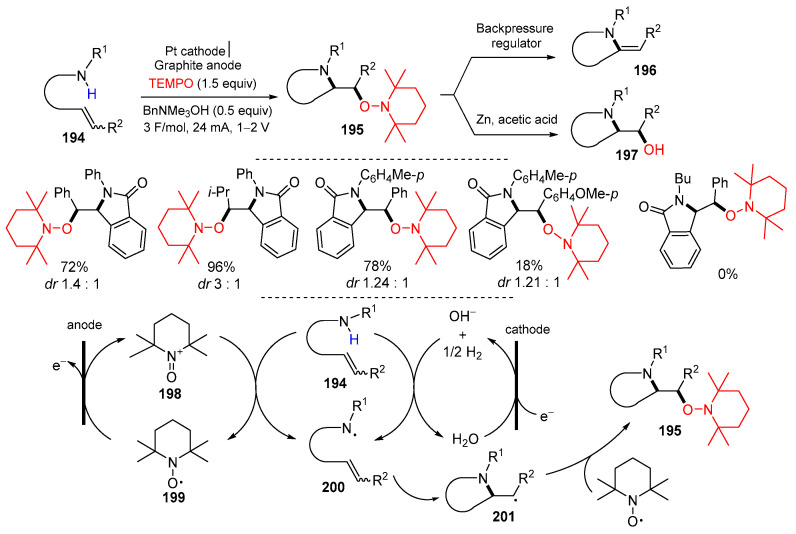
Synthesis of TEMPO-functionalized isoindolinone derivatives.

Wirth and coworkers reported an electrochemical flow reaction of carbamates for the synthesis of isoindolinone derivatives in 2017. The reaction of carbamates **194** and TEMPO using benzyltrimethylammonium hydroxide ([BnNMe_3_]^+^OH^−^) as the supporting electrolyte in an electrochemical flow micro-reactor under the optimized reaction conditions (3 F mol^−1^, 24 mA, 1–2 V) gave products **195** in good yields (Scheme 44) [69]. The isoindolinone products **195** could be used for the following two subsequent functionalizations: (1) elimination of the TEMPO moiety with Backpressure regulator to yield alkenes **196**, and (2) reduction in the N–O bond with Zn and acetic acid to yield corresponding alcohols **197**. In this reaction process, the TEMPO cation **198** reacts with carbamates **194** generating TEMPO radical **199** and nitrogen radicals **200**. Cyclization of radicals **200** to form **201** followed by a radical trapping with TEMPO gives products **195**.

**Scheme 45:**
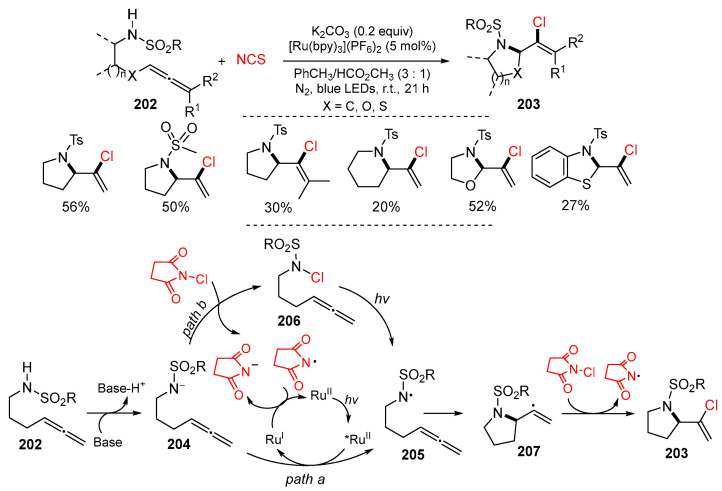
Synthesis of 2-(1-chlorovinyl)pyrrolidines.

In 2023, Renzi and coworkers reported a photo-induced radical reaction of *N*-(allenyl)sulfonylamides for the synthesis of 2-(1-chlorovinyl)pyrrolidines. The blue LEDs irradiated reactions of *N*-(allenyl)sulfonylamides **202**, *N*-chlorosuccinimide (NCS) and K_2_CO_3_ under the catalysis of [Ru(bpy)_3_](PF_6_)_2_ using anhydrous PhCH_3_ and HCO_2_CH_3_ as a co-solvent for 21 h gave products 2-(1-chlorovinyl)pyrrolidines **203** in good yields (Scheme 45) [70]. After the initial deprotonation of allenes **202** with a base to form **204**, there are two different pathways for the formation of radicals *N*-centered radicals **205**. In path a, radicals **205** are generated from the oxidation of **204** with the photoexcited state of the Ru-catalyst. In the path b, reaction of **204** with NCS to form **206** followed by the photodissociation of Cl gives **205**. The 5-*exo* cyclization of radical **205** affords vinyl radical **207** followed by radical trapping to give final 2-(1-chlorovinyl)pyrrolidines **203**. 

### 3.3. Halo-Substituted Alkenes, Alkynes and Allenes as Substrates

**Scheme 46:**
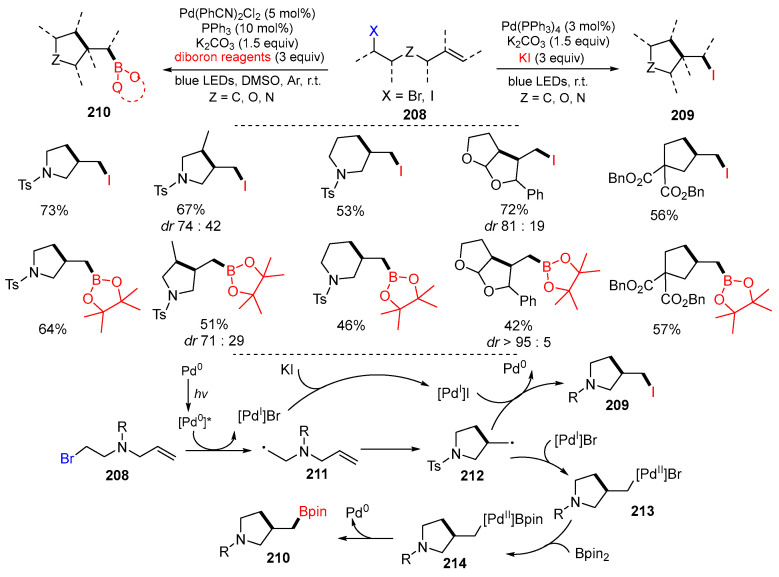
Synthesis of heterocyclic compounds.

Halogenated alkenes, alkynes and allenes are good substrates to generate carbon radicals for cyclative difunctionalization reactions. Liang and coworkers, in 2020, introduced a visible-light-induced radical reaction of alkyl bromides or alkyl iodine for the synthesis of heterocyclic compounds. The reaction of alkyl bromides or iodines **208** and KI with Pd(PPh_3_)_4_ as a photo-catalyst and potassium carbonate as a base under the irradiation of blue LEDs afforded heterocyclic compounds **209** in moderate yields (Scheme 46) [71]. Under the conditions of using Pd(PhCN)_2_Cl_2_ as a catalyst and potassium carbonate as a base, the reaction with diboron reagents gave borylated products **210** in moderate yields. In the reaction process for products **209**, the [Pd^0^]^*^-promoted homolytic cleavage of the aryl C−Br bond of **208** yields radicals **211** which undergo 5-*exo* cyclization to form radicals **212** followed by iodiozation with [Pd^I^]I to give products **209**. For the borylation reaction, recombination of radicals **212** with [Pd^I^]Br gives Pd-complexes **213** which undergo transmetalation with bis(pinacolato)diboron to form Pd-complexes **214**. Finally, the reductive elimination of Pd-cat of **214** affords borylated products **210**. 

**Scheme 47:**
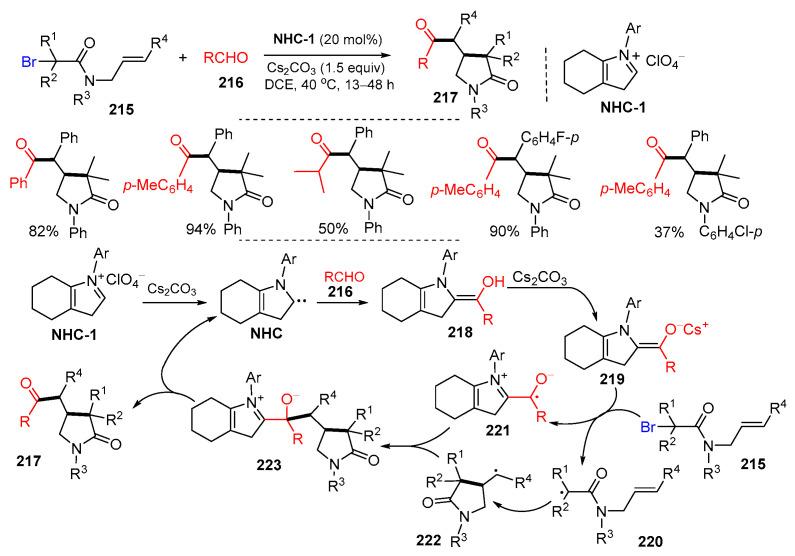
Synthesis of 2-pyrrolidinone derivatives.

In 2023, Yao and coworkers reported an *N*-heterocyclic carbene (NHC)-catalyzed radical reaction of *α*-bromo-*N*-cinnamylamides for the synthesis of 2-pyrrolidinone derivatives. The reaction of *α*-bromo-*N*-cinnamylamides **215** and aldehydes **216** in the presence of **NHC** precursor **NHC-1** and Cs_2_CO_3_ in DCE at 40 °C for 13–48 h afforded 2-pyrrolidinone derivatives **217** in good to excellent yields (Scheme 47) [72]. The **NHC** catalyst generated by the reaction of catalyst precursor **NHC-1** with Cs_2_CO_3_ couples with aldehyde **216** to form intermediates **218** which are then deprotonated with Cs_2_CO_3_ to give enolate intermediates **219**. A process of SET of *α*-bromo-*N*-cinnamylamides **215** with **219** produces the radical zwitterionic intermediates **220** and radical intermediates **221**. The 5-*exo* cyclization of the radicals **220** gives **222** followed by radical coupling with the radicals **221** to provide **223** and the last step of **NHC** catalyst elimination to give products **217**.

**Scheme 48:**
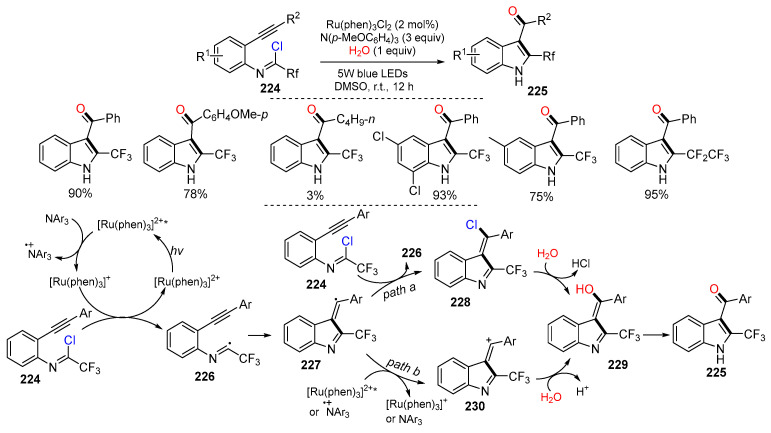
Synthesis of 2-trifluoromethyl-3-acylindoles.

Zhou and coworkers, in 2015, reported a visible-light-induced radical reaction of trifluoroacetimidoyl chlorides for the synthesis of 2-trifluoromethyl-3-acylindoles. The reaction of trifluoroacetimidoyl chlorides **224** in the presence of Ru(phen)_3_Cl_2_, (*p*-MeOC_6_H_4_)_3_N (Ar_3_N), and H_2_O in DMSO under the irradiation of 5 W blue LEDs at room temperature gave products **225** in good to excellent yields (Scheme 48) [73]. The proposed reaction mechanism suggested that imidoyl radicals **226** produced by the C–Cl bond cleavage of trifluoroacetimidoyl chlorides **224** undergo a 5-*exo* cyclization to form vinyl radicals **227** which then go through two different pathways to generate 2-CF_3_ indoles **225**. For the favorable pathway (path a), the reaction of vinyl radicals **227** and trifluoroacetimidoyl chlorides **224** afford vinyl chlorides **228** followed by hydrolysis to give enols **229** and then isomerization to give **225**. While in the less favorable pathway (path b), vinyl cations **230** are formed by the oxidation of radicals **227** with excited [Ru(phen)_3_]^2+*^ or Ar_3_N radical cation followed by trapping with H_2_O to yield enols **229** and then products **225** after isomerization. 

**Scheme 49:**
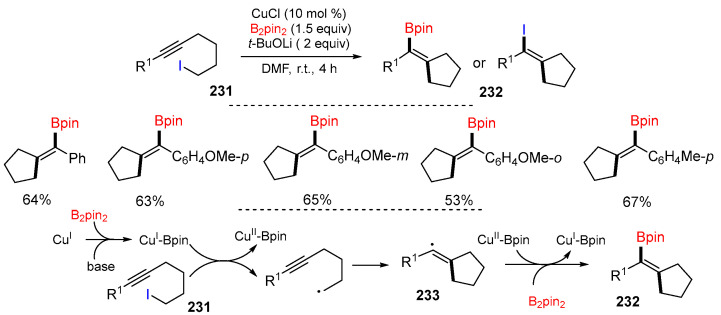
Synthesis of exocyclic alkenes.

In 2018, Zhao and coworkers introduced a Cu-catalyzed radical reaction of acetylenic iodides for the synthesis of functionalized exocyclic alkenes. The reaction of acetylenic iodides **231** and B_2_pin_2_ in the presence of CuCl and *t*-BuOLi in DMF at room temperature for 4 h gave products **232** in good yields (Scheme 49) [74]. The proposed reaction mechanism suggested that the reaction of acetylenic halides **231** with Cu^I^–Bpin followed by a 5-*exo* cyclization affords intermediates **233** which then react with the Cu^II^ species to give borylated products **232**.

**Scheme 50:**
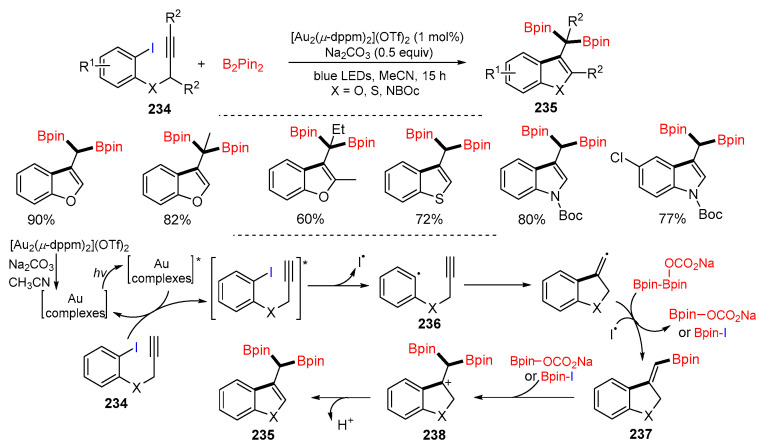
Synthesis of *gem*-diboronated heterocyclic compounds.

A visible-light-induced radical reaction of aryl iodides for the synthesis of benzofuran-, indole-, and benzothiophene-based benzylic *gem*-diboronates was reported by the Hashmi group in 2020. The reaction of aryl iodides **234** and B_2_Pin_2_ under the irradiation of blue LEDs in the presence of [Au_2_(*μ*-dppm)_2_](OTf)_2_ and Na_2_CO_3_ in MeCN for 15 h afforded products **235** in good to excellent yields (Scheme 50) [75]. The photoactivated Au-complex promotes the homolytic cleavage of the C−I bond of aryl iodide **234** to give aryl radicals **236** which undergo 5-*exo* cyclization followed by radical trapping to give vinyl boronates **237**. Formation of benzyl cations **238** by electrophilic borylation of boronates **237** with Bpin−I or Bpin−OCO_2_Na followed by rapid *β*-H elimination gives products **235**.

**Scheme 51:**
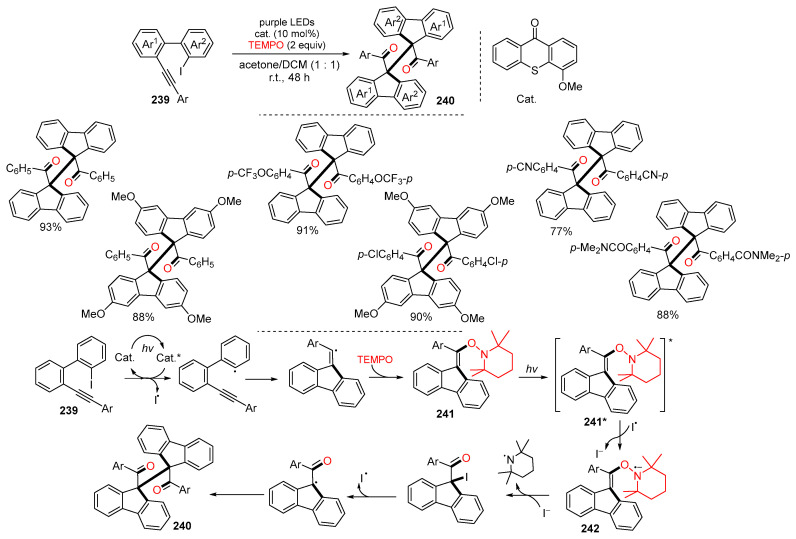
Synthesis of 1,4-dicarbonyl compounds.

The Guo group reported a visible-light-induced radical reaction of alkyne-containing aryl iodides for the synthesis of 1,4-dicarbonyl compounds. The reaction of alkyne-containing aryl iodides **239**, TEMPO and 4-methoxythioxanthone under the irradiation of purple LEDs using acetone/DCM as solvent gave products **240** in good to excellent yields (Scheme 51) [76]. Under the irradiation of purple LEDs, the photosensitizer 4-methoxythioxanthone converts substrates **239** to triplet state 239^*^ to form aryl radicals for 5-*exo* cyclization followed by trapping with TEMPO to afford intermediates **241**. The photo-excited triplet state intermediates 241^*^ undergo a single-electron reduction and coupling with I^−^ to give intermediates **242** followed by removal of iodine radical and biradical coupling to provide 1,4-dicarbonyl compounds **240**.

**Scheme 52:**
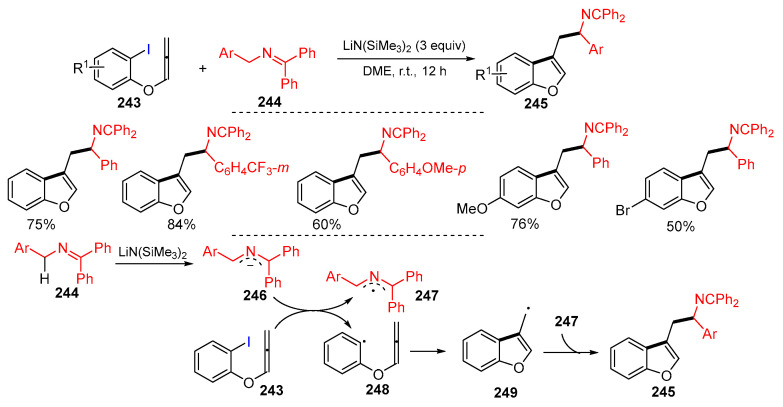
Synthesis of substituted benzofurans.

**Scheme 53:**
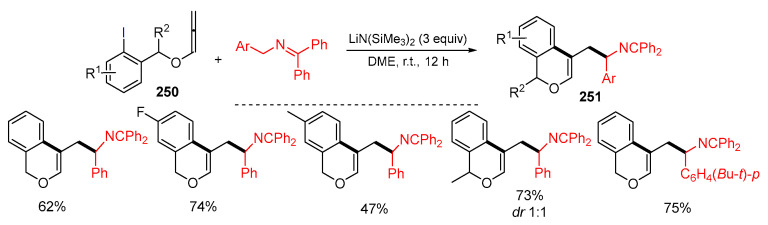
Synthesis of substituted isochromenes.

A radical reaction of 2-iodoaryl-allenyl ethers for the synthesis of benzofuran derivatives was reported by the Walsh group in 2019. The reaction of 2-iodoaryl-allenyl ethers **243** and ketimines **244** in the presence of LiN(SiMe_3_)_2_ in DME for 12 h gave products **245** in good to excellent yields (Scheme 52) [77]. In the reaction process, 2-azaallyl anions **246** generated from ketimines **244** serve as a “super electron donor” (SED). The SET of **246** to aryl iodides **243** generates aryl radicals **248** which undergo 5-*exo* cyclization to form 2-azaallyl radicals **249** followed by trapping radicals **247** to afford products **245**. The same group extended the scope of this reaction using 2-iodobenzyl allenyl ethers **250** as substrates for the synthesis of substituted isochromenes **251** (Scheme 53) [78].

### 3.4. Other Functionalized Alkenes and Allenes as Substrates

**Scheme 54:**
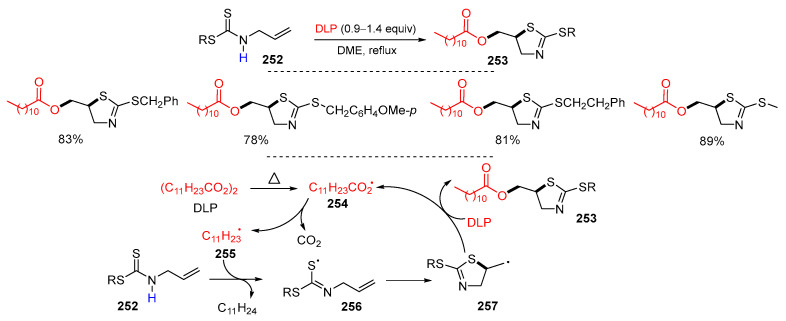
Synthesis of (2-alkylthiothiazolin-5-yl)methyl dodecanoates.

In addition to the substrates presented above, compounds used for the formation of initial radicals could be used as the source for the second functionalization. Xu and Kakaei in 2013 reported a radical reaction of alkyl *N*-allylcarbamodithioates for the synthesis of (2-alkylthiothiazolin-5-yl)methyl dodecanoates. The reaction of alkyl *N*-allylcarbamodithioates **252** and dilauroyl peroxide (DLP) in refluxing DME for 4–7 h gave products **253** in good yields (Scheme 54) [79]. The heating of DLP gives lauroyl radical **254** followed by decarboxylation to form undecyl radical **255**. H-abstraction of **255** from *N*-allylcarbamodithioates **252** generates thiyl radicals **256** which undergo 5-*exo* cyclization to yield (2-alkylthiothiazolin-5-yl)-methyl radicals **257**. Final products (2-alkylthiothiazolin-5-yl)methyl dodecanoates **253** are obtained from the reaction of radicals **257** with DLP along with regeneration of the lauroyl radical **254**.

**Scheme 55:**
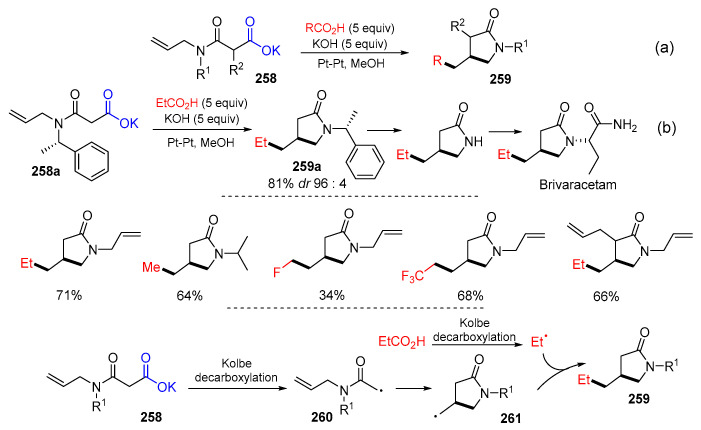
Synthesis of 4-substituted pyrrolidin-2-ones.

In 2020, Riant and coworkers reported a radical electrocyclization of potassium 3-(diallylamino)-3-oxopropanoate for the synthesis of 4-substituted pyrrolidin-2-ones. In Teflon half-cells equipped with platinum-plated electrodes, the reaction of potassium 3-(diallylamino)-3-oxopropanoates **258** in the presence of KOH and RCOOH with MeOH as solvent gave 4-substituted pyrrolidin-2-ones **259** in good yields (Scheme 55a) [80]. The application of this method for continuous flow electrochemical reaction in a loop-reactor setup (equipped with a 5 mL container) allowed for an excellent productivity of 0.40 g/(h·mL) and afforded 2-pyrrolidinone **259a** in 81% yield (Scheme 55b) [81]. The product could be used for the synthesis of Brivaracetam in 23% yield. For the synthesis of **259**, radicals **260** generated via Kolbe decarboxylation of **258** undergo 5-*exo* cyclization followed by coupling of Et radical with **261** to give the final products.

**Scheme 56:**
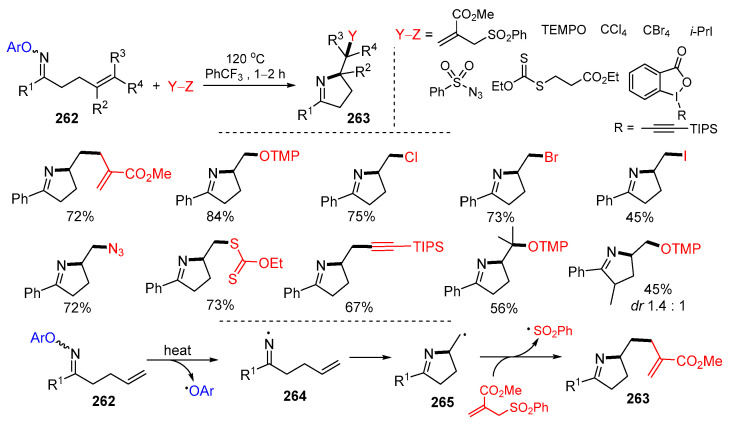
Synthesis of functionalized pyrrolines.

In 2022, Castle and coworkers developed a protocol of thermal and microwave-promoted radical cyclization of *O*-aryloximes for the synthesis of functionalized pyrrolines. The reaction was carried out using *O*-aryloximes **262** and the radical traps Y−Z in PhCF_3_ at 120 °C for 1−2 h, and the products functionalized pyrrolines **263** were obtained in good yields (Scheme 56) [82]. The reactions could be triggered by either microwave irradiation or conventional heating in an oil bath. Initially, the iminyl radicals **264** are generated by direct homolysis of the weak N−O bond of *O*-aryloximes **262**, which could be promoted by either microwave irradiation or conventional heating. Subsequently, radicals **265** are formed by a 5-*exo* radical cyclization of radicals **264**, followed by radical trapping to give the final functionalized pyrrolines **263**.

**Scheme 57:**
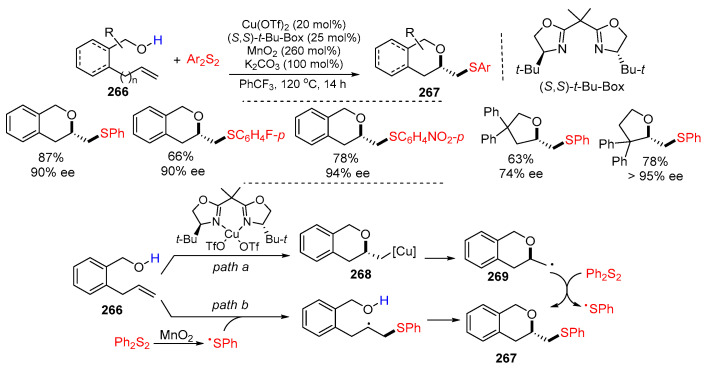
Asymmetric synthesis of arylthiomethylated cyclic ethers.

In 2022, Chemler and coworkers reported a Cu-catalyzed radical reaction of alkenols for the synthesis of arylthiomethyl-substituted cyclic ethers. The reaction of alkenols **266** and diaryl disulfides in the presence of Cu(OTf)_2_, (*S*,*S*)-*t*-Bu-Box, MnO_2_ and K_2_CO_3_ in PhCF_3_ at 120 °C for 14 h afforded arylthiomethyl substituted cyclic ethers **267** in moderate to good yields and high enantioselectivity (Scheme 57) [83]. It should be mentioned that the target compound was obtained in a lower yield without an external oxidant (MnO_2_ or O_2_). The proposed reaction mechanism suggests that the enantioselective route (path a) is lower in energy than the competing racemic route (path b). First, C−O bond formation via enantioselective oxycupration of **266** affords intermediate **268** followed by C−[Cu] homolysis to give radical intermediate **269**. The formation of C−S bond via coupling of **269** with PhS radical gives oxysulfenylated product **267**. 

**Scheme 58:**
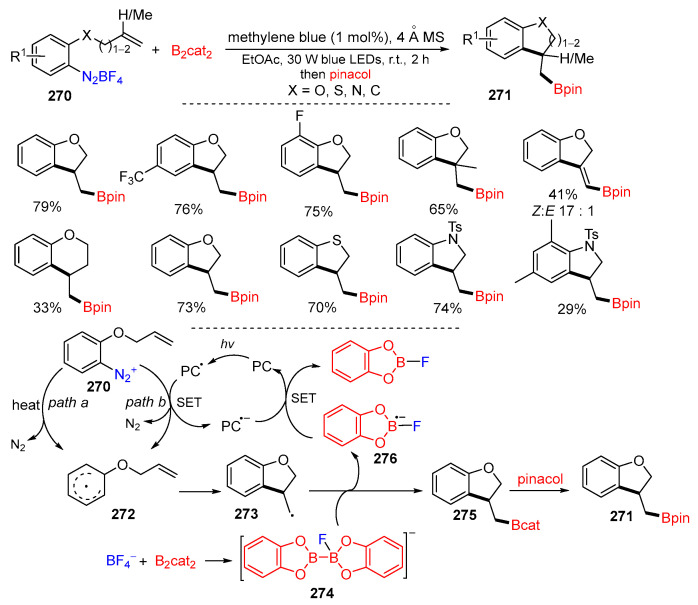
Synthesis of benzocyclic boronates.

A photo-promoted radical cyclization of alkenes for the synthesis of benzocyclic boronates was reported by the Li group in 2023. The reaction of allyl aryldiazonium salts **270** and bis(catecholato)diboron (B_2_cat_2_) using methylene blue as catalyst under the irradiation of 30 W blue LEDs in dimethylacetamide (DMA) for 2 h at room temperature followed by the treatment with pinacol gave benzocyclic boronates **271** in good yields (Scheme 58) [84]. The proposed mechanism suggests the generation of an aryl radical **272** from the diazonium salt **270** either by heat-induced decomposition (path a) or by SET through an excited photocatalyst (path b). The 5-*exo* cyclization of **272** forms benzocyclic alkyl radical **273** followed by the boron-transfer with **274** to give the borylated product **275** and the radical anion **276**. Alcohol exchange between **275** and pinacol gives the final product **271**.

**Scheme 59:**
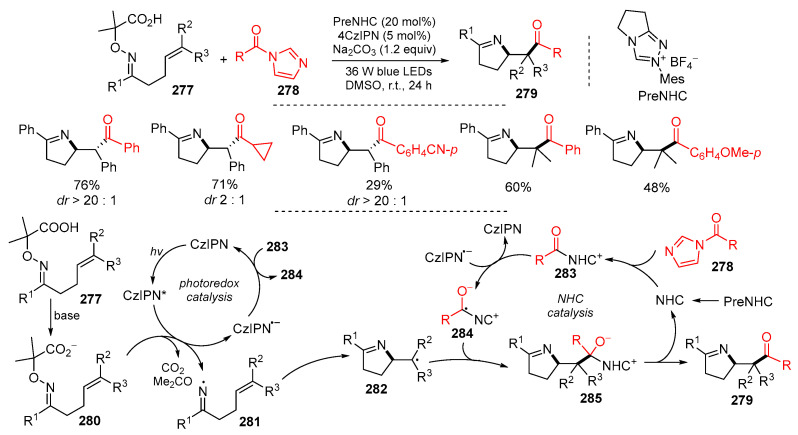
Synthesis of substituted 3,4-dihydro-2*H*-pyrroles.

In 2023, Ye and coworkers developed a protocol of photoredox *N*-heterocyclic carbene catalyzed radical cyclization of alkene-tethered *α*-imino-oxy acids for the synthesis of substituted 3,4-dihydro-2*H*-pyrroles. The reaction was carried out using alkene-tethered *α*-imino-oxy acids **277** and acyl imidazoles **278** with *N*-Mes-substituted triazolium (preNHC, Mes = 2,4,6-trimethylphenyl) as the catalyst and 2,4,5,6-tetra(9*H*-carbazol-9-yl)isophthalonitrile (4CzIPN) as photocatalyst in the presence of Na_2_CO_3_ in DMSO under irradiation of blue LEDs at room temperature for 24 h, and the products substituted 3,4-dihydro-2*H*-pyrroles **279** were obtained in moderate to good yields and with good to high diastereoselectivities (Scheme 59) [85]. Initially, the iminyl radical intermediates **281** were generated via oxidization of the carboxylate anions **280** of *α*-imino-oxy acids **277** with the excited photosensitizer 4CzIPN^*^, followed by 5-*exo* cyclization to give the dihydropyrrole-derived *C*-radicals **282**. In the meantime, the acyl azoliums **283** were formed via the addition of acyl imidazoles **278** with in situ generated free NHC, followed by reduction with 4CzIPN^•−^ to give the ketyl radicals **284** and 4CzIPN. Next, the intermediate adducts **285** were provided by the radical coupling of the radicals **282** with the radicals **284**, followed by fragmentation to deliver the final iminoacylation products **279** and release the **NHC** catalyst for the catalytic cycle.

**Scheme 60:**
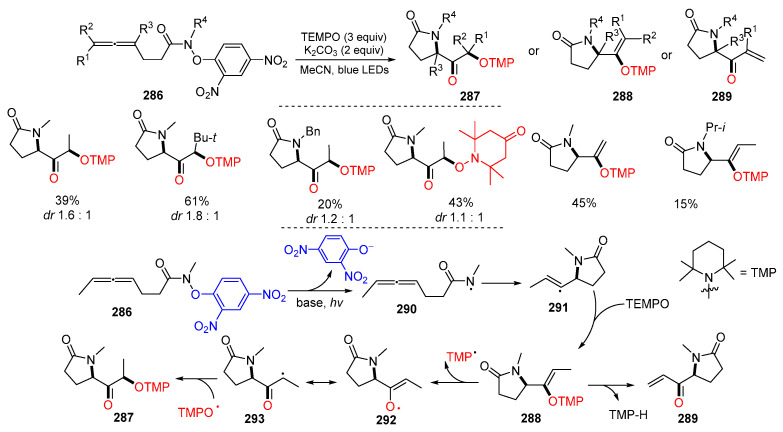
Synthesis of pyrrolidin-2-one derivatives.

In 2021, Schomaker and coworkers developed a protocol of photo-promoted radical cyclization of allenes for the synthesis of pyrrolidin-2-one derivatives. The reaction was carried out using allenes **286** and TEMPO in the presence of K_2_CO_3_ under the irradiation of blue LEDs with MeCN as solvent, and the products pyrrolidin-2-one derivatives **287**, **288** and **289** were obtained in moderate yields (Scheme 60) [86]. Initially, radical **290** was generated from allene **286** under irradiation of visible light, followed by 5-*exo* cyclization to give radical **291**. Then, product **288** was formed via radical trapping, followed by H exchange to give the enone **289**. Meanwhile, the *O*-centered radical **292** was produced by radical hemolysis of **288**, followed by electron transfer to give the *C*-centered radical **293**. Finally, product **287** was obtained by radical trapping of radical **293**.

## 4. Second Functionalization with Metal Complexes

**Scheme 61:**
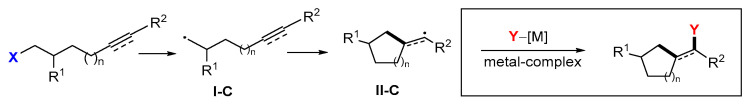
Second functionalization with metal complexes.

In this section, examples of the use of metal complexes for the second functionalization are presented (Scheme 61). In addition to Cu complexes, Pd, Ni, and Co complexes have been developed for the coupling reactions.

**Scheme 62:**
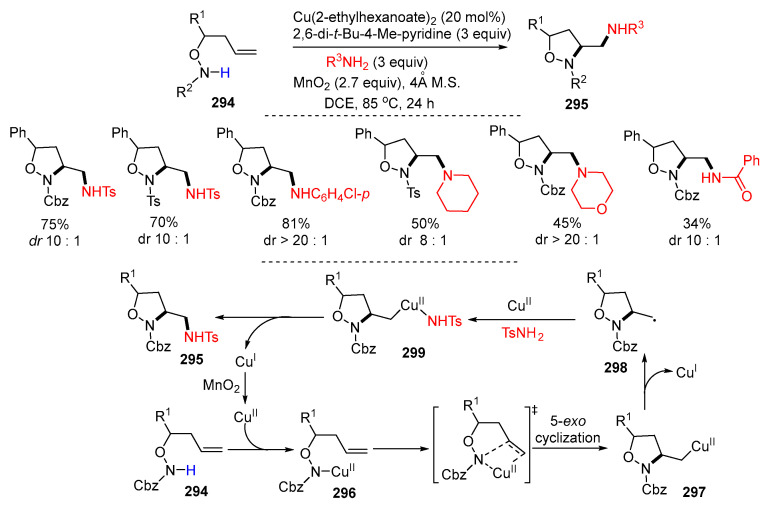
Radical cyclization for preparation of isoxazolidines.

Chemler and coworkers reported a protocol of Cu-catalyzed radical reaction of alkyne-tethered Cbz-protected hydroxylamines for the synthesis of functionalized isoxazolidines in 2017. The reaction of alkyne-tethered Cbz-protected hydroxylamines **294** and amine sources in the presence of Cu(2-ethylhexanoate)_2_, 2,6-di-*t*-Bu-4-Me-pyridine, MnO_2_ and 4Å M.S. in DCE at 85 °C for 24 h gave isoxazolidines **295** in good yields (Scheme 62) [87]. It should be mentioned that sulfonamides, anilines, piperidine, morpholine and benzamide could serve as the external amine source. The proposed reaction mechanism suggests that the reaction of **294** with Cu^II^ leads to the formation of Cu^II^-complexes **296,** which participate in 5-*exo* cyclization to form **297** intermediates, followed by homolysis of the C−Cu^II^ bond to give **298**. The reactions of **298** with Cu^II^ and amine give Cu^II^-complex **299** which then leads to the formation of isoxazolidines **295** after reductive elimination of the Cu^I^ catalyst.

**Scheme 63:**
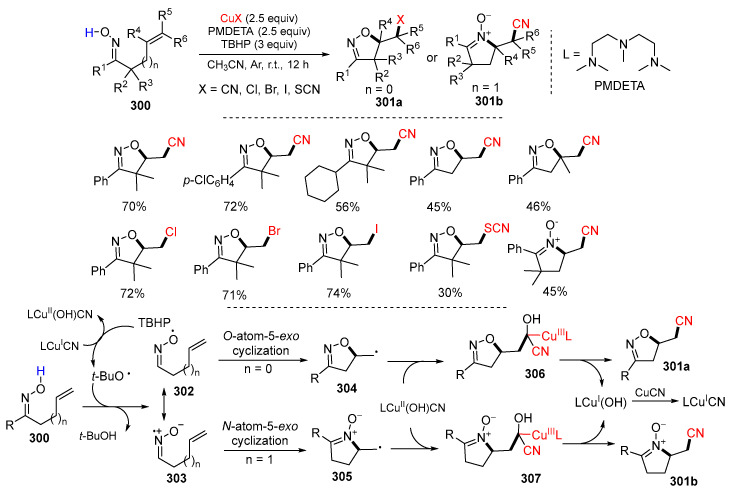
Synthesis of isoxazolines and cyclic nitrones.

Han and coworkers, in 2017, reported a radical reaction of unsaturated ketoximes for the synthesis of isoxazolines and cyclic nitrones. The reaction of unsaturated ketoximes **300** in the presence of *tert*-butyl hydroperoxide (TBHP), Cu-X and PMDETA in CH_3_CN at room temperature for 12 h gave **301** in good yields (Scheme 63) [88]. In the reaction process, *t*-BuO radical derived from TBHP reacts with oximes **300** to form iminoxyl radicals which have resonance structures **302** and **303**. Radicals **302** or **303** undergo *O*-radical 5-*exo* cyclization (n = 0) or *N*-radical 5-*exo* cyclization (n = 1) to form radicals **304** or **305** which then interact with **L**Cu^II^(OH)CN to generate Cu^III^ species **306** or **307**. Finally, products **301a** or **301b** are obtained by reductive elimination of Cu^III^ from **306** or **307** and the **L**Cu^I^CN species is regenerated.

**Scheme 64:**
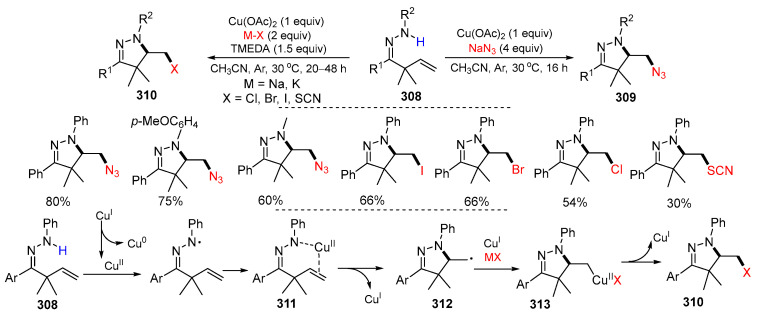
Synthesis of functionalized pyrazolines.

A Cu-mediated radical reaction of *β*,*γ*-unsaturated hydrazones for the synthesis of functionalized pyrazolines was reported by Li and coworkers in 2018. The reaction of *β*,*γ*-unsaturated hydrazones **308** and M–X (M = Na, K; X = N_3_, Cl, Br, I, SCN) and Cu(OAc)_2_ in CH_3_CN provided products **309** or **310** in good to high yields (Scheme 64) [89]. In the reaction process, HAT of *β*,*γ*-unsaturated hydrazones **308** generates *N*-radicals which coordinate with Cu^II^ to form complexes **311**. Reductive elimination of Cu^II^ of **311** followed by a radical cyclization give radicals **312** which couple with Cu^I^ and M–X to provide Cu^II^ complexes **313** and then products **310** after reductive elimination of the Cu^I^ catalyst.

**Scheme 65:**
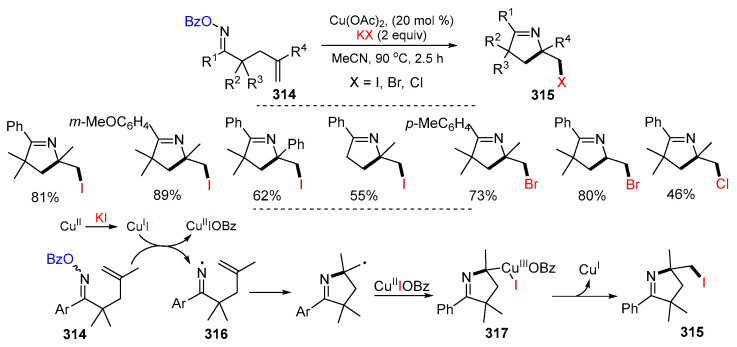
Synthesis of pyrroline derivatives.

Zhu and coworkers, in 2019, introduced a Cu-catalyzed radical reaction of unsaturated oxime esters for the synthesis of 2-halomethyl pyrrolines. The reaction of *γ*, *δ*-unsaturated oxime esters **314** and halide salts (such as KI, KBr and KCl) and Cu(OAc)_2_ in CH_3_CN at 120 °C for 2.5 h gave 2-halomethyl pyrrolines **315** in good yields (Scheme 65) [90]. The proposed reaction mechanism suggested that iminoxyl radicals **316** generated from oxime esters **314** undergo 5-*exo* cyclization followed by coordination with Cu^II^IOBz to form complexes **317** followed by reductive elimination to afford products **315**. 

**Scheme 66:**
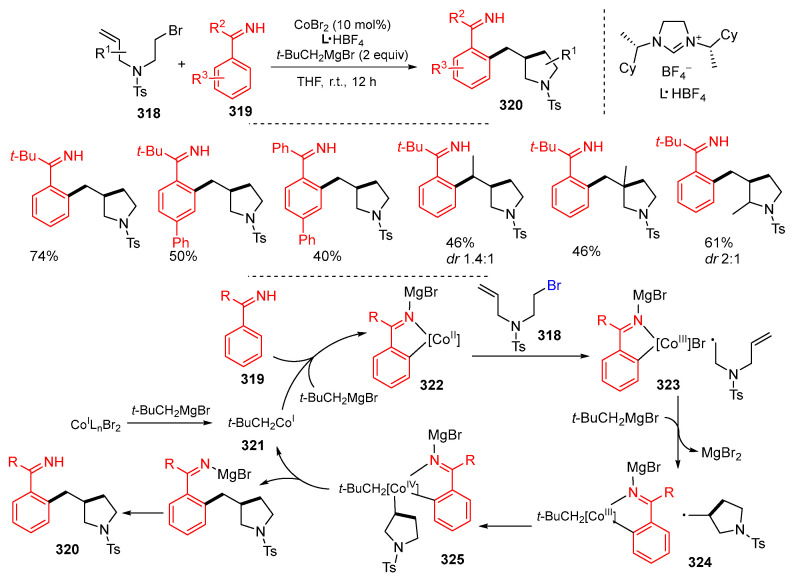
Synthesis of pyrrolidine derivatives.

In 2019, Yoshikai and coworkers reported a Co−*N*-heterocyclic carbene catalyzed radical reaction of tosylamide-tethered bromo-alkenes for the synthesis of 3-(arylmethyl)pyrrolidine derivatives. The reaction of tosylamide-tethered bromoalkenes **318** and aryl N−H imines **319** in the presence of CoBr_2_, NHCs bearing cyclohexylethyl groups (**L**) and *t*-BuCH_2_MgBr in THF for 12 h afforded products **320** in good yields (Scheme 66) [91]. In the reaction process, *t*-BuCH_2_Co^I^ **321** is generated from the reaction of Co^I^**L**_n_Br_2_ and the Grignard reagent *t*-BuCH_2_MgBr. It then reacts with N−H imines **319** and *t*-BuCH_2_MgBr to give Co^II^ complexes **322** and then reacts with bromoalkene **318** to form complex pairs **323** which undergo 5-*exo* cyclization and transmetalation with the Grignard reagent to give radical complex pairs **324** and then **325**. Products **320** are generated from **325** after reductive elimination of the Co catalyst and hydrolysis of the MgBr moiety. 

**Scheme 67:**
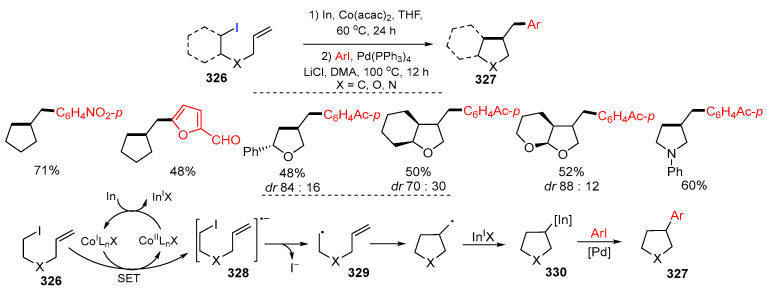
Radical cyclization for preparation of cyclic compounds.

In 2020, Shen, Han and their coworkers reported a metal-catalyzed radical reaction of iodoalkyl-tethered unactivated alkenes for the synthesis of a wide variety of cyclic compounds, including substituted cyclopentanes, furans, pyrrolidines, octahydro-1*H*-indenes, octahydro-benzofurans, hexahydro-4*H*-furo[2,3-*b*]pyrans, and hexahydro-furo[2,3-*b*]furans. The reaction of iodoalkyl-tethered alkenes **326** in the presence of In powder and Co(acac)_2_ at 60 °C for 24 h in THF followed by the treatment with ArI in the presence of Pd(PhPh_3_)_4_ and LiCl in DMA at 100 °C for 12 h gave products **327** in moderate to good yields (Scheme 67) [92]. The proposed reaction mechanism suggested that Co^I^L_n_X converts olefin-tethered alkyl iodides **326** to alkyl radical anions **328** and then alkyl radical **329** after elimination of iodine anion. Cyclization of radicals **329** followed by radical trapping with In^I^X gives alkyl indium reagent **330** which then undergoes Pd-catalyzed cross-coupling with aryl iodides to form products **327**.

**Scheme 68:**
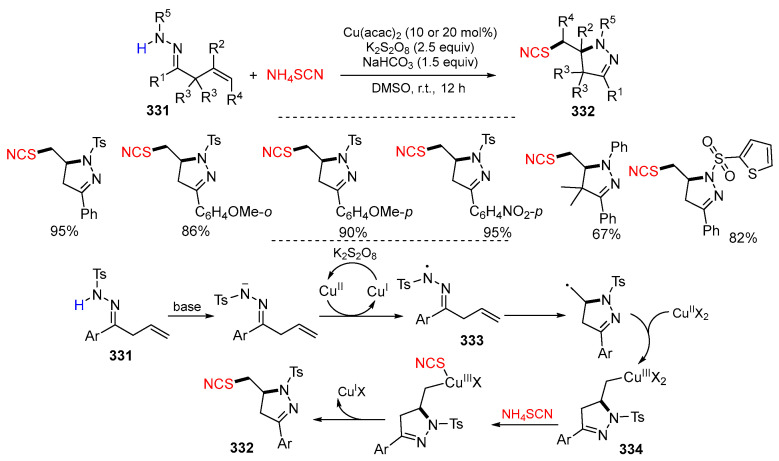
Synthesis of SCN-containing pyrazolines.

Zhu and coworkers reported a metal-catalyzed radical reaction of unactivated alkenes for the synthesis of SCN-substituted pyrazolines. The reaction of alkenes **331**, NH_4_SCN, Co(acac)_2_, K_2_S_2_O_8_, and NaHCO_3_ in DMSO at room temperature for 12 h gave products **332** in good to excellent yields (Scheme 68) [93]. In the reaction process, ketoximes **331** are deprotonated with a base and oxidized by Cu^II^ to iminoxyl radicals **333** for 5-*exo* cyclization followed by the reaction with Cu^II^X_2_ to form the Cu^III^ complexes **334**. The reactions of **334** with NH_4_SCN followed by reductive elimination afford products **332**.

**Scheme 69:**
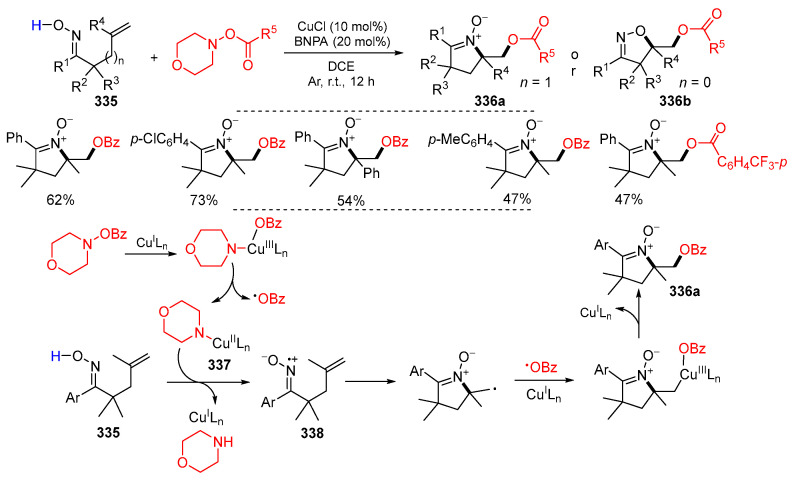
Radical cyclization for preparation of cyclic nitrones.

Wang and coworkers reported a Cu-catalyzed radical reaction of unsaturated ketoximes for the synthesis of cyclic nitrone products in 2021. The reaction of ketoximes **335** and morpholino benzoates in the presence of CuCl and 1,1-binaphthyl-2,2-diyl hydrogen-phosphate (BNPA) with DCE as a solvent, and the cyclic nitrone gave products **336** in good yields (Scheme 69) [94]. In the synthesis of **336a**, Cu^II^ complex **337** generated from morpholino benzoate and Cu^I^Ln reacts with ketoximes **335** (n = 1) to form radicals **338** via oxidative SET. Cyclization of **338** followed by coupling with OBz radical and then reductive elimination give final products **336a**.

**Scheme 70:**
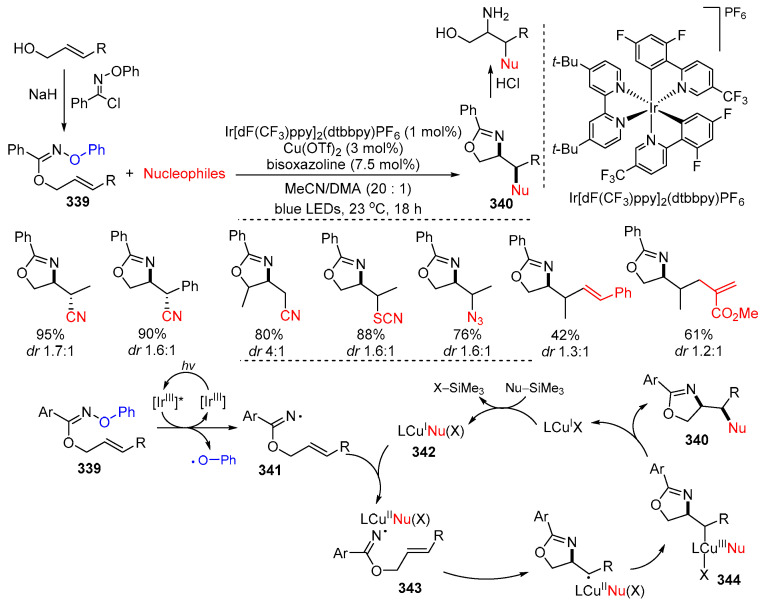
Synthesis of functionalized oxazolines.

An Ir and Cu dual-catalysts-promoted photo reaction of oxime of allyl alcohols for the synthesis of functionalized oxazolines was developed by the Nagib group in 2021. The reaction of oxime **339** derived from allyl alcohols with Ir[dF(CF_3_)ppy]_2_(dtbbpy)PF_6_ and Cu(OTf)_2_ in the presence of bisoxazoline and nucleophiles such as CN, SCN, N_3_, vinyl, allyl and under the irradiation of blue LEDs for 18 h in 20:1 MeCN/DMA, gave functionalized oxazolines **340** in moderate to good yields (Scheme 70) [95]. The functional oxazolines **340** could be hydrolyzed with HCl to *β*-amino-*γ*-cyano alcohols. 

A dual catalytic mechanism was proposed for this reaction. Oxime **339** undergoes [Ir^III^]^*^-catalyzed homolysis to form radicals **341** that react with LCu^I^Nu(X) **342** to give Cu^II^ complex **343**. The 5-*exo* cyclization of *N*-centered radicals **343**, followed by radical trapping, yields Cu^III^ species **344**, which form products **340** after reductive elimination of the CuI catalyst. 

**Scheme 71:**
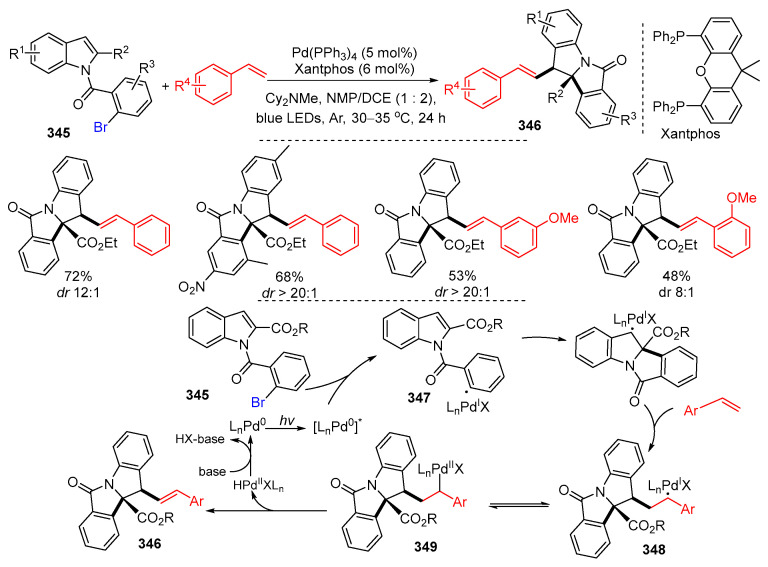
Synthesis of indoline derivatives.

A Pd-catalyzed radical reaction of *N*-(2-bromobenzoyl)indoles for the synthesis of 2,3-disubstituted indolines was introduced by the Sharma group in 2020 (Scheme 71) [96]. The reaction of *N*-(2-bromobenzoyl)indoles **345** and styrenes with Pd(PPh_3_)_4_, Xantphos and Cy_2_NMe in *N*-methyl-2-pyrrolidone (NMP)/DCE under the irradiation of blue LEDs for 24 h gave 2,3-disubstituted indoline derivatives **346** in moderate to good yields and good to excellent diastereoselectivities. The proposed mechanism indicated that aryl radicals **347** produced via a SET reduction in aryl bromides **345** undergo 5-*exo* radical cyclization followed by the addition of styrenes to give hybrid alkyl [Pd^I^] radicals **348**. The radicals **348** have equilibrium structures **349** which undergo *β*-H elimination to give products **346**.

**Scheme 72:**
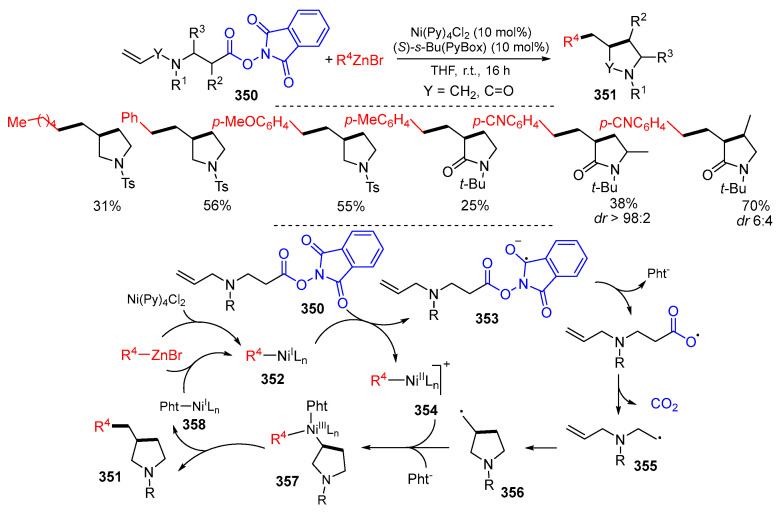
Synthesis of functionalized pyrrolidines and pyrrolidinones.

In 2022, Cárdenas and coworkers reported a Ni-catalyzed radical reaction of allylamines and acryl-amides containing an active ester group for the synthesis of nitrogen heterocycles. The reaction of allylamines or acryl-amides **350**, alkylzinc or arylzinc bromides, Ni(Py)_4_Cl_2_ and (*S*)-*s*-Bu(PyBox) in THF at room temperature for 16 h afforded pyrrolidines and pyrrolidinones **351** in good yields (Scheme 72) [97]. In this reaction, [RNi^I^L_n_] complexes **352** produced by the reaction of Ni(py)_4_Cl_2_ and RZnBr undergo SET with esters **350** to form radical anions **353** and [RNi^I^L_n_]^+^ complexes **354**. Homolytic N−O bond cleavage of radical anions **353** followed by decarboxylation gives radicals **355** which undergo 5-*exo* cyclization to form **356**. Further reaction of **356** with **354** and Pht^−^ originates [Ni^III^] complexes **357**. Products **351** are obtained by reductive elimination of complexes **357** while the [Ni^I^] complex **358** could react with RZnBr to regenerate the [RNi^I^L_n_] complexes **352** for the catalytic cycle.

## 5. Second Functionalization with Y of Neutral Molecules

**Scheme 73:**
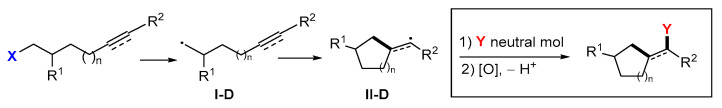
Second functionalization with Y from neutral molecules.

Presented in this section are the reactions in which the second functionalization is accomplished by reacting with neutral molecules Y followed by a reduction in the resulting radicals to anions and then deprotonation to give the final products (Scheme 73). Alkenes, alkynes, arenes, molecular oxygen (O_2_), sulfur dioxide (SO_2_), 2-isocyanobiaryl, CO, B_2_(OH)_4_, and glycine derivatives could serve as the neutral molecules for the second functionalization reactions. 

### 5.1. Alkenes, Alkynes and Arenes as Y

**Scheme 74:**
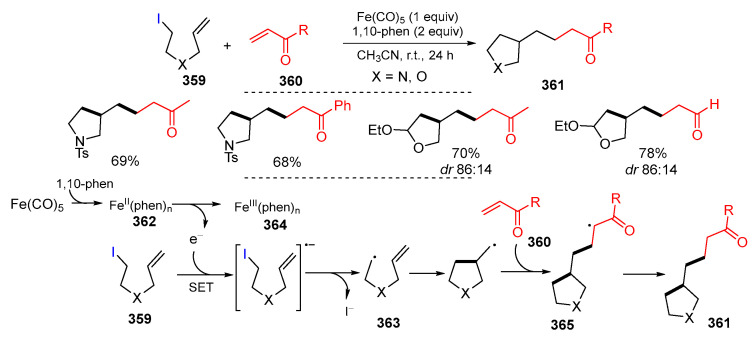
Synthesis of heterocyclic compounds.

There are several examples of using alkenes, alkynes and arenes for the second functionalization reactions. In 2016, Kang and coworkers reported a radical reaction of *ω*-iodoalkenes for the synthesis of heterocyclic compounds. The reaction of *ω*-iodoalkenes **359** and *α*,*β*-unsaturated carbonyl compounds **360** in the presence of Fe(CO)_5_ and 1,10-phen monohydrate in CH_3_CN for 24 h gave products **361** in good to excellent yields (Scheme 74) [98]. The proposed reaction mechanism suggests that complex **362**, generated from Fe(CO)_5_ and 1,10-phen, reacts with alkyl iodides **359** to form alkyl radical intermediates **363** which undergo 5-*exo* cyclization. Subsequent radical addition to the *β*-C in the *α*,*β*-unsaturated carbonyl compound **360** produces the *α*-carbonyl radicals **365** and then products **361**.

**Scheme 75:**
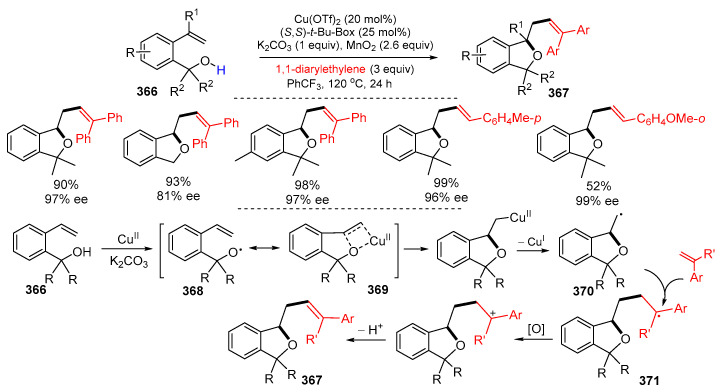
Synthesis of phthalans.

A Cu-catalyzed asymmetric reaction of 2-vinylbenzyl alcohols for the synthesis of phthalans was reported by Chemler and Chen in 2018. The reaction of 2-vinylbenzyl alcohols **366** and 1,1-diarylethylene in the presence of Cu(OTf)_2_, (*S*,*S*)-*t*-Bu-Box, K_2_CO_3_, and MnO_2_ in PhCF_3_ at 120 °C for 24 h gave phthalan products **367** in good to high yields and enantioselectivity (Scheme 75) [99]. Radicals **368** generated from benzylalcohols **366** coordinate with Cu^II^ to give intermediates **369** and lead to the formation of radicals **370** after a 5-*exo* cyclization. The addition of **370** to arylethylenes gives benzylic radicals **371** followed by oxidation and H-elimination to afford products **367**.

**Scheme 76:**
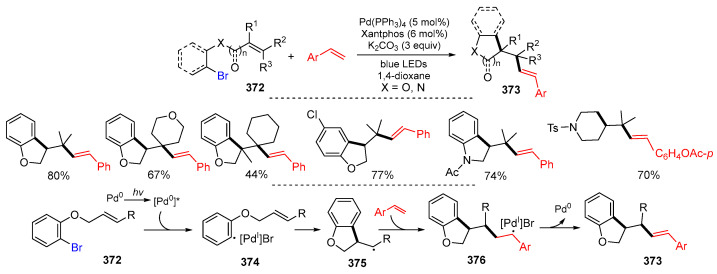
Synthesis of substituted dihydrobenzofurans.

Glorius and coworkers, in 2020, reported a visible-light-induced radical reaction of prenylated 2-bromophenols for the synthesis of substituted dihydrobenzofurans. The reaction of 2-bromophenols **372** and styrenes with Pd(PPh_3_)_4_ as photo-catalyst, xantphos as an auxiliary ligand, and potassium carbonate as a base, and 1,4-dioxane as a solvent and under irradiation of blue LEDs gave products **373** in good to excellent yields (Scheme 76) [100]. In the reaction process, photo-excited [Pd^0^]^*^ induces the homolytic cleavage of aryl C−Br bond of **372** to form radicals **374** for the subsequential 5-*exo* cyclization to give radical intermediates **375**. The addition of radicals **375** to styrene derivatives followed by substituted dihydrobenzofurans **373** was obtained via *β*-H elimination of the intermediates **376**, and the photo-catalyst Pd^0^ was regenerated for the catalytic cycle.

**Scheme 77:**
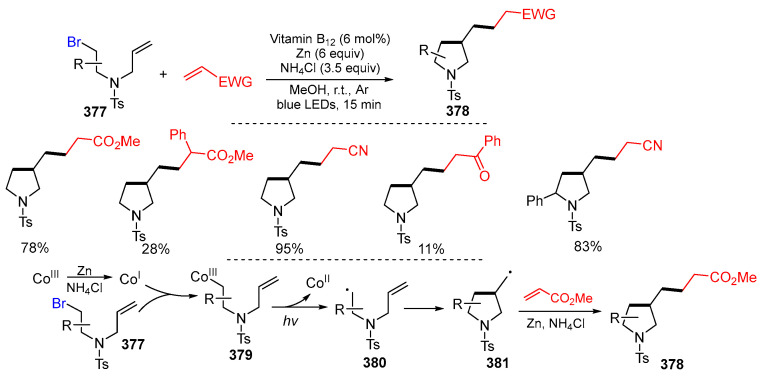
Synthesis of functional pyrrolidines and piperidines.

In 2021, Gryko and coworkers introduced a vitamin B_12_-catalyzed radical reaction of bromoalkenes for the synthesis of substituted pyrrolidines and piperidines. Under the irradiation of blue LEDs, the vitamin B_12_-catalyzed reaction of bromoalkenes **377** and electrophilic olefins in the presence of Zn and NH_4_Cl in MeOH for only 15 min afforded substituted pyrrolidines **378** in low to good yields (Scheme 77) [101]. The reaction of **377** with Co^I^ which is derived from vitamin B_12_ leads to the formation of Co-complexes **379** and radicals **380** for 5-*exo* cyclization to form radicals **381**. The addition of radicals **381** to an electron-deficient olefin followed by a single electron reduction with the Zn affords products pyrrolidines **378**.

**Scheme 78:**
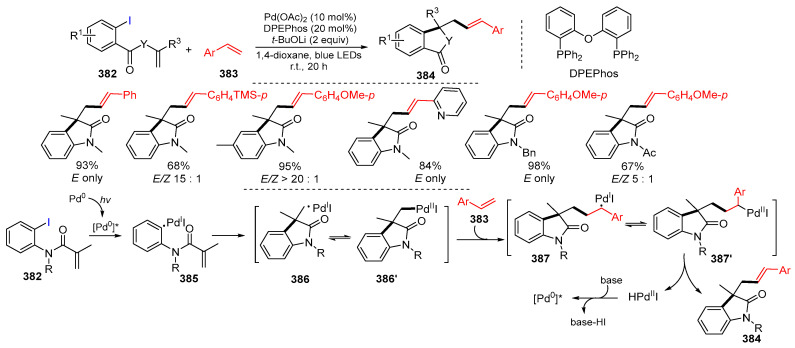
Synthesis of functionalized oxindoles.

A photo and Pd-catalyzed radical cyclization of *N*-(2-iodo-aryl) acrylamides for the synthesis of diverse oxindole scaffolds was reported by Chen, Teng and their coworkers in 2023. The reaction of *N*-(2-iodo-aryl) acrylamides **382** and vinyl arenes **383** in the presence of Pd(OAc)_2_, DPEPhos and *t*-BuOLi in 1,4-dioxane under the irradiation of blue LEDs at room temperature for 20 h gave oxindole products **384** in good to excellent yields (Scheme 78) [102]. In the proposed reaction mechanism the visible light-excited [Pd^0^]^*^ reacts with **382** to provide aryl hybrid Pd-radical intermediates **385** which leads to the formation of alkyl hybrid Pd-radical species **386** and **386′** after 5-*exo* cyclization. The reaction of styrene **383** with 3**86′** to form **387** and **387′** followed by *β*-H elimination gives products **384**.

**Scheme 79:**
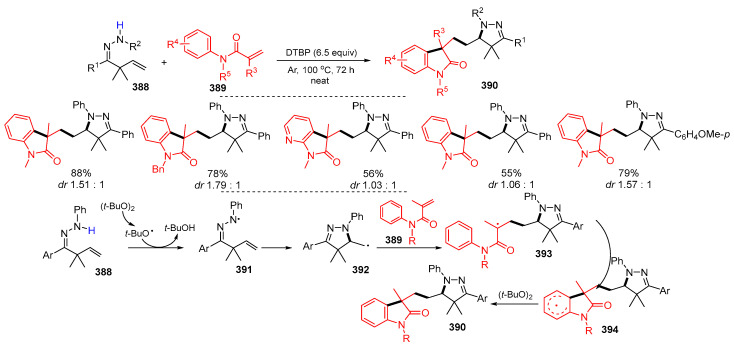
Synthesis of pyrazoline-functionalized oxindoles.

**Scheme 80:**
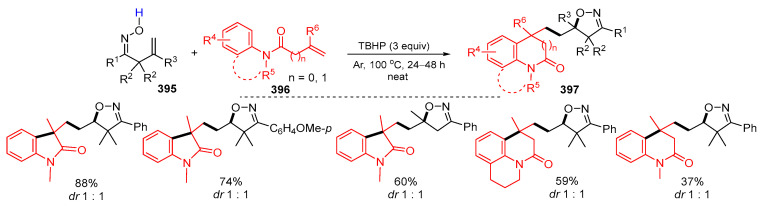
Synthesis of isoxazoline-bearing oxindoles and dihydroquinolinones.

Han and coworkers introduced a radical reaction of *β*,*γ*-unsaturated hydrazones for the synthesis of pyrazoline-functionalized oxindoles in 2015. The reaction of *β*,*γ*-unsaturated hydrazones **388** and *N*-aryl acrylamides **389** in the presence of DTBP (*di*-*tert*-butyl peroxide) under the solvent-free conditions at 100 °C for 72 h gave products **390** in good to excellent yields (Scheme 79) [103]. The *t*-BuO radical derived from DTBP abstracts H from *β*,*γ*-unsaturated hydrazones **388** to give radicals **391** followed by cyclization to form radicals **392**. The addition of **392** to *N*-aryl acrylamides **389** form radicals **393** followed by the second cyclization and oxidative aromatization with DTBP yield pyrazoline-functionalized oxindoles **390**. The same group extended the scope for the reaction of unsaturated ketoximes for the synthesis of isoxazoline-functionalized oxindoles and dihydroquinolinones. The reaction of unsaturated ketoximes **395** and *N*-arylpropiolamides or *N*-arylacrylamides **396** in the presence of TBHP at 100 °C for 24–48 h gave products **397** in good to excellent yields (Scheme 80) [104].

**Scheme 81:**
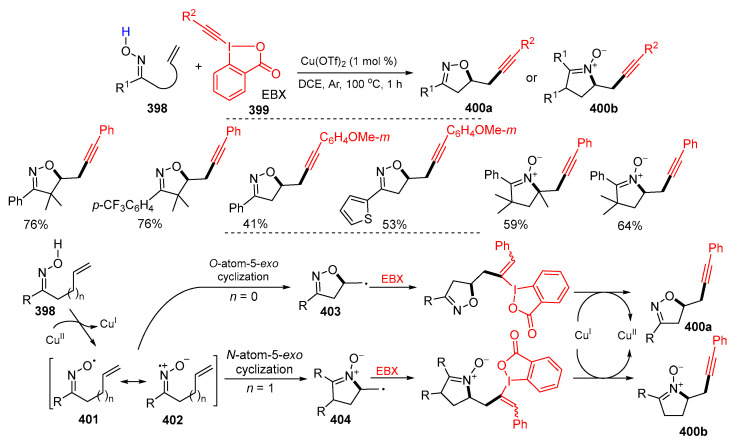
Synthesis of isoxazolines and cyclic nitrones.

Han and coworkers reported a Cu-catalyzed reaction of unsaturated ketoximes for the synthesis of alkynylated isoxazolines and cyclic nitrones. The reaction of unsaturated ketoximes **398** and ethynylbenziodoxolones (EBX) **399** in the presence of Cu(OTf)_2_ in DCE under Ar at 100 °C for 1 h gave products **400** in moderate to good yields (Scheme 81) [105]. In the reaction process, Cu^II^ catalysis converts ketoximes **398** to iminoxyl radicals which have resonance structures **401** and **402**. Subsequent *O*-/*N*-atom 5-*exo* cyclizations of **401** and **402** yield radical intermediates **403** and **404**, respectively. The addition of **403** or **404** at alkyne moiety of EBX **399** followed by the Cu^I^-assisted elimination of 2-iodobenzoate gives products **400a** or **400b**.

**Scheme 82:**
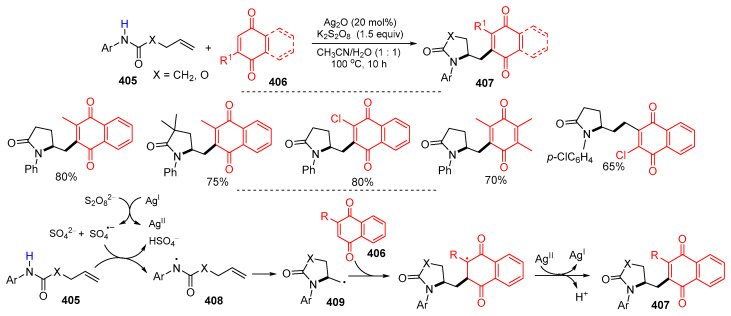
Synthesis of substituted quinones.

An Ag-catalyzed radical reaction of unactivated olefins for the synthesis of substituted quinone was reported by Li and coworkers in 2022. The reaction of unactivated olefins **405** (such as *N*-aryl-4-pentenamides and *N*-aryl allyl carbamates) and quinones **406** in the presence of Ag_2_O and K_2_S_2_O_8_ in CH_3_CN/H_2_O at 100 °C for 10 h gave substituted quinone products **407** in good to excellent yields (Scheme 82) [106]. Amidyl radicals **408** generated via H-abstraction of *N*-aryl-4-pentenamides **405** by SO_4_^•−^ radical undergo 5-*exo* cyclization to give radicals **409** which then add to menadiones **406** followed by an Ag^II^-induced HAT to give products **407**.

**Scheme 83:**
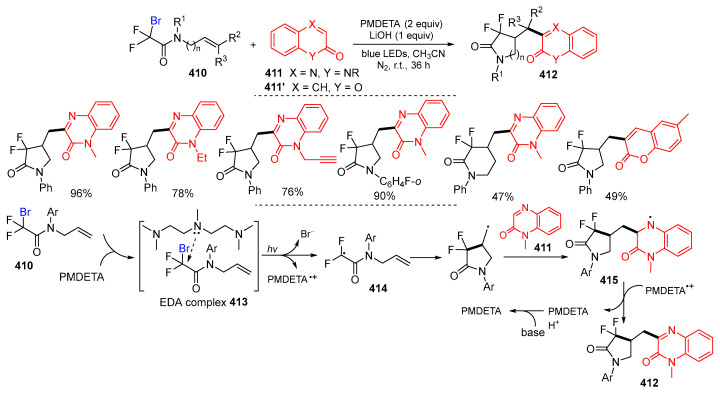
Synthesis of *α*,*α*-difluoro-*g*-lactam-fused heterocyclic compounds.

In 2023, Chen, Huang and their coworkers reported a photo-induced radical reaction of *N*-allyl-2-bromo-2,2-difluoroacetamides for the synthesis of *α*,*α*-difluoro-*g*- lactam-fused quinoxalin-2(1*H*)-ones and coumarins. The reaction of *N*-allyl-2-bromo- 2,2-difluoroacetamides **410** and quinoxalin-2(1*H*)-ones **411** or coumarins **411’** in the presence of PMDETA and LiOH in CH_3_CN under the irradiation of 24 W blue LEDs for 36 h gave *α*,*α*-difluoro-*g*-lactam-fused quinoxalin-2(1*H*)-ones or coumarins **412** in moderate to excellent yields (Scheme 83) [107]. In this reaction process, PMDETA plays a dual role as an electron donor and hydrogen atom transfer reagent. The EDA (electron donor-acceptor) complexes **413** generated from **410** and PMDETA lead to the formation of difluoroalkyl radicals **414** after SET under the irradiation of blue LEDs. The 5-*exo* cyclization of radicals **414** followed by radical addition to quinoxalin-2(1*H*)-one **411** afford intermediates **415** which undergo H-abstraction with PMDETA^·+^ to afford products **412**.

### 5.2. O_2_ as Y

**Scheme 84:**
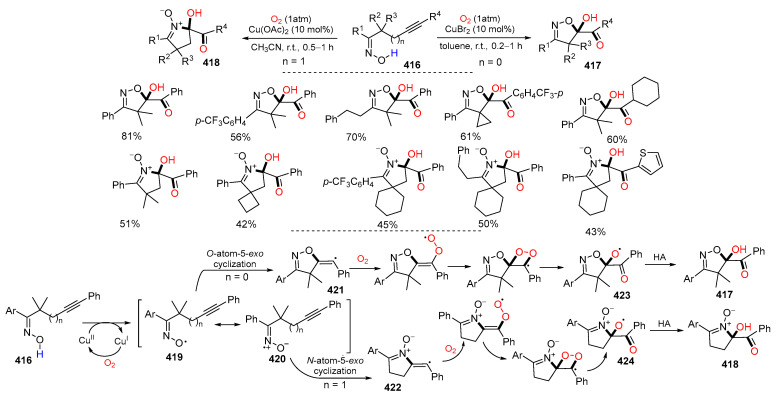
Synthesis of hydroxylated isoxazolines and dihydropyrrole oxides.

**Scheme 85:**
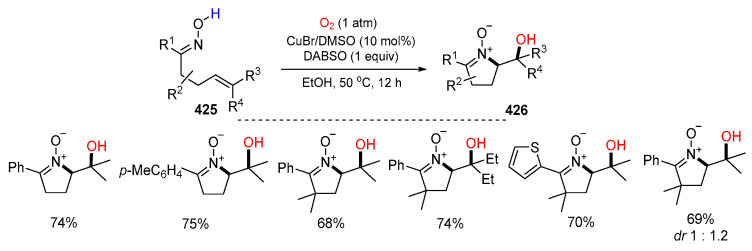
Synthesis of cyclic nitrones.

Using molecular oxygen for the second functionalization could introduce a hydroxy or carbonyl group to the products. Han and coworkers in 2017 reported a Cu-catalyzed radical reaction of oxime-tethered alkynes for the synthesis of hydroxylated isoxazolines and dihydropyrrole oxides. The reaction of oxime-tethered alkynes **416** with O_2_ in the presence of CuBr_2_/toluene or Cu(OAc)_2_/CH_3_CN gave products **417** or **418** in good yields (Scheme 84) [108]. The proposed mechanism involves the generation of iminoxyl radicals, *O*-radical **419** and *N*-radical **420** resonance structures, via SET between Cu^II^ and oximes **416**. Cyclization of **419** or **420** afford alkenyl radicals **421** or **422** followed by trapping of O_2_ to yield the alkoxy radicals **423** or **424** after cyclization of peroxy radicals followed by the O−O bond cleavage. Products **417** and **419** are generated from the H abstraction of **423** and **424**, respectively. A related reaction of unsaturated oximes for the synthesis of hydroxylated dihydropyrrole oxides was reported by Li’s group. The reaction of unsaturated oximes **425** and O_2_ in the presence of CuBr/DMSO and DABSO (1,4-diazoniabicyclo[2.2.2]-octane-1,4-disulfinate) in EtOH at 50 °C for 12–24 h gave hydroxylated dihydropyrrole oxides **426** in good yields (Scheme 85) [109]. 

**Scheme 86:**
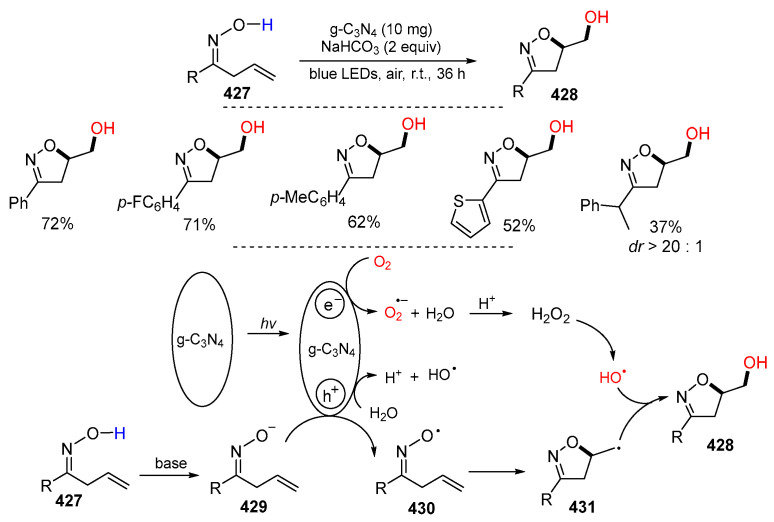
Synthesis of isoxazolines.

A visible-light-induced radical reaction of *β,γ*-unsaturated oximes for the synthesis of isoxazolines by Yu, Chen and coworkers in 2022. The reaction of *β,γ*-unsaturated oximes **427** using graphitic carbon nitride (*g*-C_3_N_4_) as photocatalyst in the presence of NaHCO_3_ in CH_3_CN/H_2_O under the irradiation of 10 W blue LEDs for 36 h gave OH-decorated isoxazolines **428** in moderate to good yields (Scheme 86) [110]. In the reaction process, the photo irradiation of the semiconductor *g*-C_3_N_4_ generates an electron and a hole. Then, a single-electron oxidation of the hole in the valence band with water forms hydroxyl radical and proton, while in the conduction band, the single-electron reduction in the electron and the O_2_ in air gives superoxide anion and water. The superoxide radical anion is protonated to produce H_2_O_2_ which then is converted to a HO radical. Intermediates **429** produced by the reaction of allyl oximes **427** with base undergo SET to the hole to generate radical intermediates **430** and then radicals **431** after cyclization. The coupling of HO radical and **431** gives final products **428**.

**Scheme 87:**
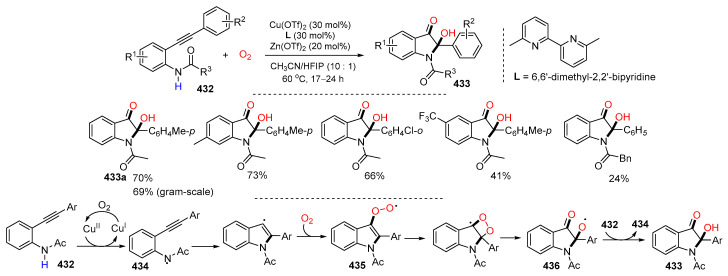
Synthesis of 2-hydroxy-2-indol-3-ones.

A Cu-catalyzed reaction of 2-arylethynylanilines for the synthesis of substituted 2-hydroxy-2-indol-3-ones was reported by Wu, Chen and their coworkers in 2023. The reaction of 2-arylethynylanilines **432** and O_2_ in the presence of Cu(OTf)_2_, Zn(OTf)_2_ and 6,6′-dimethyl-2,2′-bipyridine CH_3_CN/HFIP (hexafluoro-*iso*-propanol) at 60 °C for 17–24 h afforded substituted 2-hydroxy-2-indol-3-ones **433** in good yields (Scheme 87) [111]. This reaction could be carried out at a gram-scale under the standard conditions to give product **433a** in 69% yield. In the reaction process, the *N*-center radicals **434** generated via H-abstraction from 2-arylethynylanilines **432** undergo 5-*endo* cyclization followed by reaction with O_2_ to form peroxic species **435** for radical cyclization and subsequential O–O bond cleavage to form radicals **436**. H-abstraction of **436** from substrates **432** gives products **433** while radicals **434** are regenerated for the reaction cycle. 

**Scheme 88:**
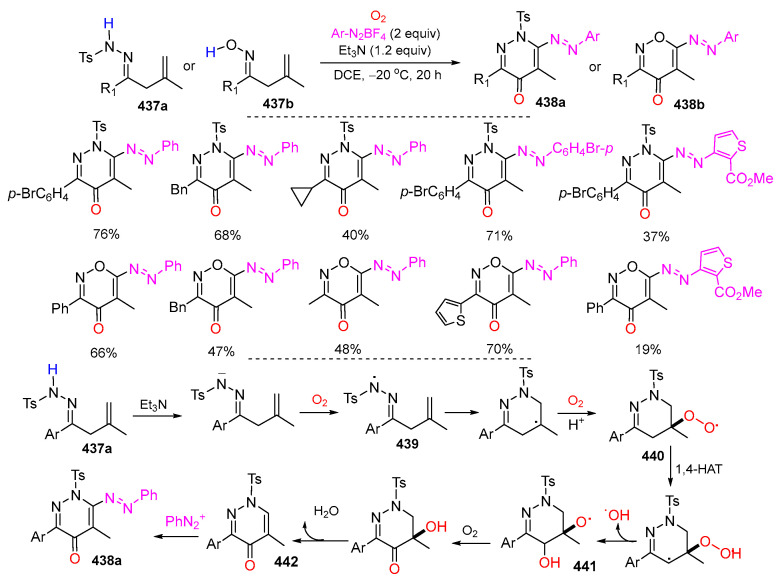
Synthesis of substituted pyridazin-4(1*H*)-ones and oxazin-4(1*H*)-ones.

A radical reaction of unsaturated hydrazones/ketoximes for the synthesis of heterocyclic compounds such as pyridazin-4(1*H*)-ones or oxazin-4(1*H*)-ones was reported by Jiang and coworkers in 2023. The reaction of *β*,*γ*-unsaturated hydrazones **437a** or ketoximes **437b** and diazonium tetrafluoroborates in the presence of Et_3_N and O_2_ in DCE at −20 °C for 24 h gave pyridazin-4(1*H*)-ones **438a** or oxazin-4(1*H*)-ones **438b** in good to excellent yields (Scheme 88) [112]. In the synthesis of **438a**, anionic intermediates generated from hydrazones **437a** via deprotonation of NHTs with Et_3_N are oxidized by O_2_ to form *N*-centered radicals **439** followed by 6-*endo* cyclization and capture with O_2_ to form peroxide radicals **440**. Then, 1,4-HAT of **440** gives *C*-radicals followed by the cleavage of the O−O bond to give radicals **441** which are oxidized, followed by dehydration and then reaction with aryldiazonium species to give products **438a**.

### 5.3. SO_2_ from DABSO as Y

**Scheme 89:**
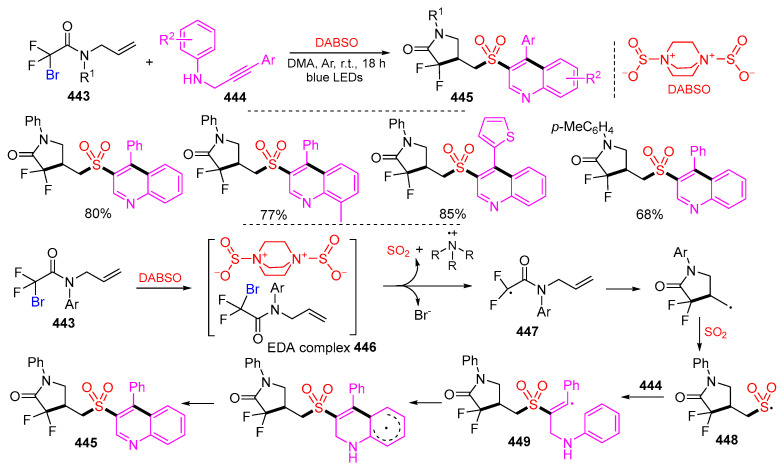
Synthesis of difluoroamidosulfonylated quinolines.

Similar to the molecular oxygen, SO_2_ from DABSO could be used as a reactant for the second functionalization to introduce the sulfonyl group to the products. Ye and coworkers in 2023 developed a visible light-induced photocatalyst-free reaction of *N*-allylbromodifluoroacetamides for the synthesis of difluoroamidosulfonylated quinolones. The reaction of *N*-allylbromodifluoroacetamides **443**, *N*-propargylamine **444** and DABSO under the irradiation of 40 W blue LEDs for 18 h in DMA at room temperature gave difluoroamidosulfonylated quinolines **445** in moderate to good yields (Scheme 89) [113]. In the reaction process, EDA complexes **446** generated from *N*-allylbromo- difluoroacetamides **443** and DABSO under SET afford difluoroalkyl radicals **447** for 5-*exo* cyclization followed by the reaction with SO_2_ to give difluoroamidosulfonyl radicals **448** which then react with *N*-propargylamines **444** to form vinyl radicals **449** followed by radical cyclization and deprotonative aromatization to afford products **445**.

**Scheme 90:**
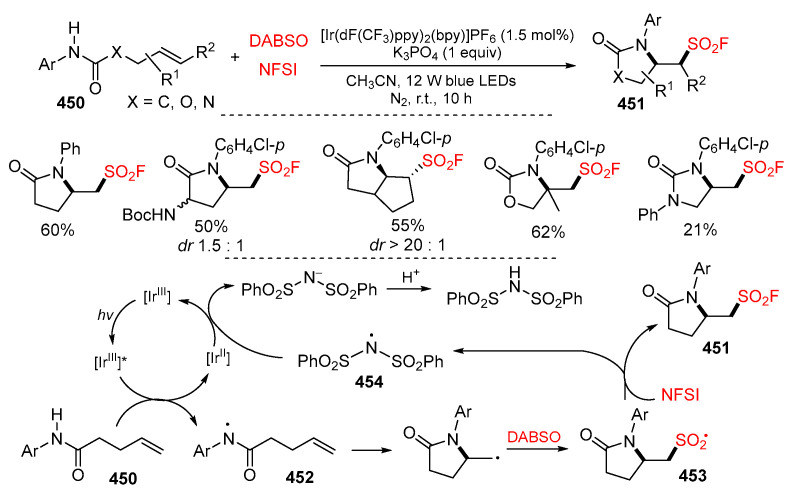
Synthesis of SO_2_F-functionalized heterocyclic compounds.

**Scheme 91:**
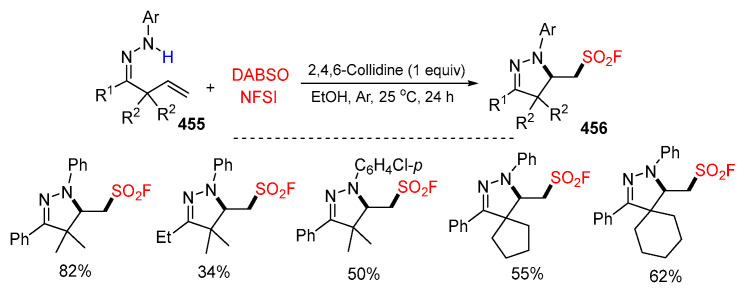
Synthesis of SO_2_F-functionalized pyrazolines.

In 2021, Weng and coworkers reported a photoredox-catalyzed reaction of unactivated olefins for the synthesis of SO_2_F-attached 5-membered heterocyclic compounds. The reaction of unactivated olefins **450** such as *N*-phenyl pent-4-enamide, DABSO and *N*-fluorobenzenesulfonimide (NFSI) in the presence of [Ir(dF(CF_3_)ppy)_2_(bpy)]PF_6_ and K_3_PO_4_ in CH_3_CN under the irradiation of blue LEDs for 10 h gave products **451** in good to excellent yields (Scheme 90) [114]. Amidyl radicals **452** generated from *N*-phenyl pent-4-enamides **450** via SET with [Ir^III^]^*^ undergo 5-*exo* cyclization followed by radical trapping with SO_2_ of DABSO to give alkylsulfonyl radicals **453**. These trap the fluorine atom from NFSI to give products **451** with concomitant generation of (PhSO_2_)_2_N radical **454** which is converted to (PhSO_2_)_2_NH by oxidation of [Ir^II^] to [Ir^III^]. Wang and coworkers reported a similar reaction of unsaturated hydrazones for the synthesis of SO_2_F-functionalized pyrazolines. The reaction of *β*,*γ*-unsaturated hydrazones **455**, DABSO and NFSI in the presence of 2,4,6-collidine, EtOH under Ar at 25 °C for 24 h gave products **456** in good to excellent yields (Scheme 91) [115]. Products **456** could be further transformed into sulfonate esters and amides.

### 5.4. Y from Other Neutral Molecules 

**Scheme 92:**
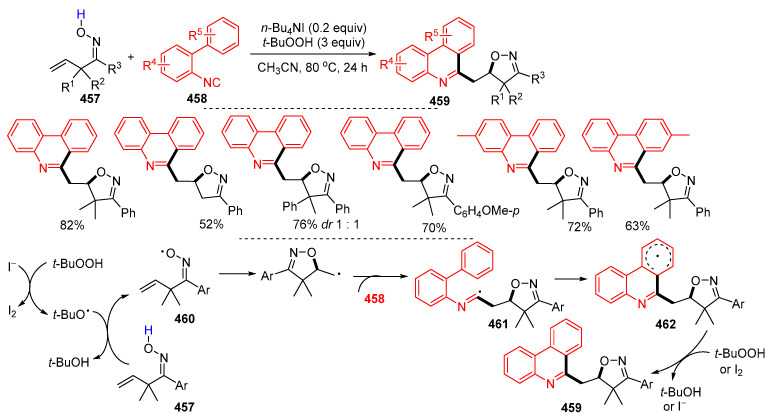
Synthesis of isoxazoline-functionalized phenanthridines.

Other than alkenes, molecular oxygen and SO_2_ described above, isocyanides, imines, CO, and B_2_(OH)_4_ could be used for the second functionalization reactions. The Han group reported a radical reaction of *β*,*γ*-unsaturated ketoximes for the synthesis of isoxazoline-functionalized phenanthridines in 2014. The reaction of *β*,*γ*-unsaturated ketoximes **457** and 2-arylphenylisonitriles **458** in the presence of *t*-BuOOH and *n*-Bu_4_NI in CH_3_CN at 80 °C for 24 h gave products **459** in good yields (Scheme 92) [116]. In the reaction process, *t*-BuO radical and I_2_ are produced by the reaction of *t*-BuOOH with *n*-Bu_4_NI with concomitant H-abstraction of oxime **457** to yield iminoxyl radicals **460** for 5-*exo* cyclization to form radicals. Then, these are added to 2-isocyanobiaryl **458** to yield imidoyl radicals **461** that cyclize to the adjacent phenyl ring to form radicals **462** which undergo oxidative aromatization with I_2_ or TBHP to afford products **459**.

**Scheme 93:**
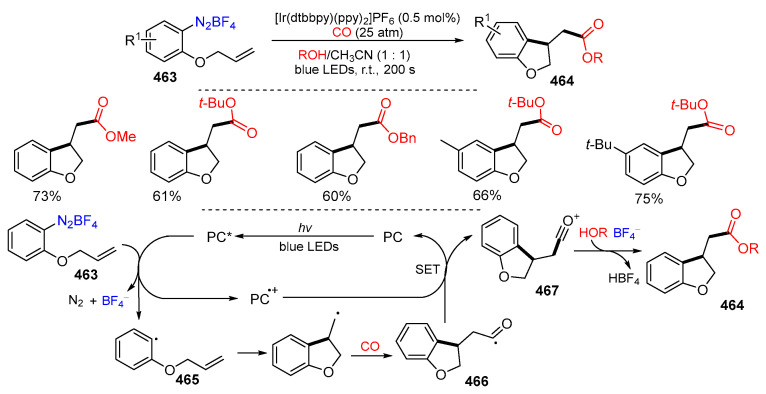
Synthesis of 2,3-dihydrobenzofurans derivatives.

In 2018, Polyzos and coworkers reported a continuous-flow reaction of alkenyl-tethered arenediazonium salts for the synthesis of 2,3-dihydrobenzofurans. The flow reaction of alkenyl-tethered arenediazonium salts **463**, CO and ROH in the presence of [Ir(dtbbpy)(ppy)_2_]PF_6_ in CH_3_CN under the irradiation of blue LEDs gave products **464**. The reaction can be completed in a short time of 200 seconds and is scalable (Scheme 93) [117]. In the reaction process, photo-exited catalyst PC^*^ promoted the reaction of diazonium tetrafluoroborates **463** to form radicals **465** which undergo 5-*exo* cyclization to yield radicals. The latter traps CO to give acyl radicals **466** which are oxidized with PC^•+^ to **467** and then react with ROH and BF_4_^−^ to afford products **464**. 

**Scheme 94:**
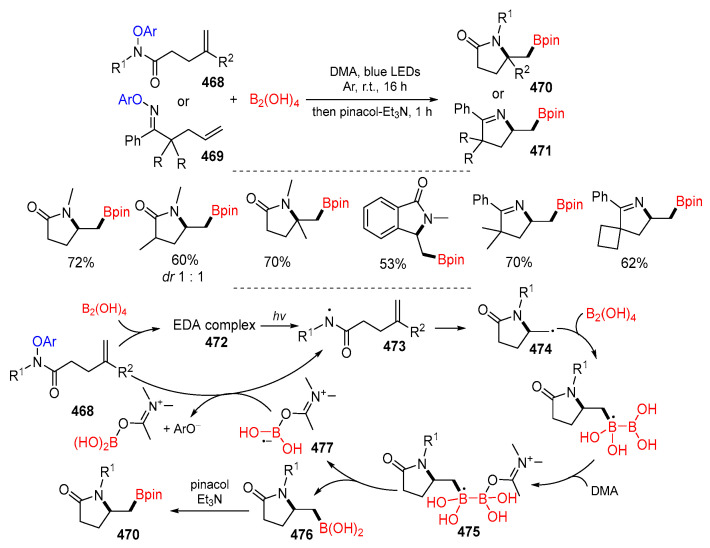
Synthesis of cyclic 1,2-aminoboronic esters.

Studer and coworkers reported a radical reaction of unactivated alkenes for the synthesis of cyclic 1,2-aminoboronic esters. The reaction of unsaturated aryloxy-amides **468** or ketoximes **469** and B_2_(OH)_4_ under the irradiation of blue LEDs with DMA as a solvent gave cyclic boronic esters **470** or **471** in good yields (Scheme 94) [118]. The proposed mechanism suggested that EDA complexes **472** generated from the reaction of **468** and B_2_(OH)_4_ are converted to radicals **473** under the photo conditions and then undergo 5-*exo* cyclization to form radicals **474**. The reactions of **474** with B_2_(OH)_4_ and then with DMA produce radicals **475** followed by the homolysis of weak B−B bond to give boronic acids **476** along with the DMA-stabilized B-centered radical **477**. The reactions of boronic acids **476** with pinacol and Et_3_N give cyclic boronic esters **470**.

**Scheme 95:**
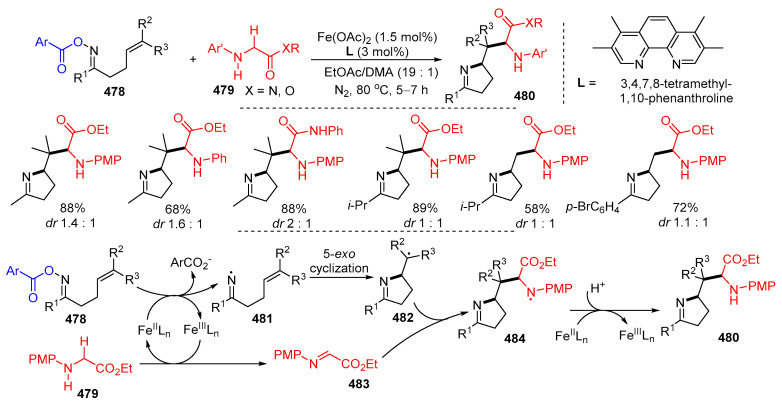
Synthesis of heterocyclic compounds.

In 2023, Gong and Lu and coworkers reported a Fe-catalyzed radical reaction of unsaturated oxime esters for the synthesis of pyrroline-containing amino acid derivatives. The reaction of *γ*,*δ*-unsaturated oxime esters **478** and glycine derivatives **479** in the presence of Fe(OAc)_2_ and 3,4,7,8-tetramethyl-1,10-phenanthroline in EtOAc/DMA at 78–80 °C for 5–7 h gave pyrroline-containing amino acids derivatives **480** in good to excellent yields (Scheme 95) [119]. The iminyl radicals **481** generated via the reductive cleavage of N–O bond of oxime ester **478** undergo 5-*exo* cyclization to give alkyl radicals **482** and then react with imines **483** generated from **479** to give *N*-centered radicals **484**. Final products **480** are obtained via SET of Fe^II^L_n_ to radicals **484**. 

## 6. Second Functionalization with Other Forms of Y (Y^•+^, Y^+^, and Y^−^)

**Scheme 96:**
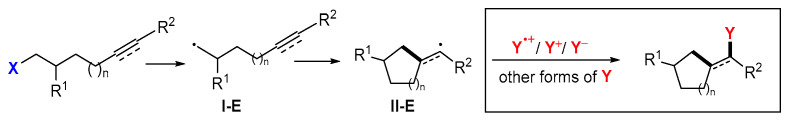
Second functionalization with other forms of Y.

In the cyclative radical difunctionalization reactions, the cyclized radicals could be converted to cations, anions or other reactive species for the second functionalization with the right counterparts. Presented in this section are the reactions using Y^•+^, Y^+^ and Y^−^ for the second functionalization reactions (Scheme 96). 

**Scheme 97:**
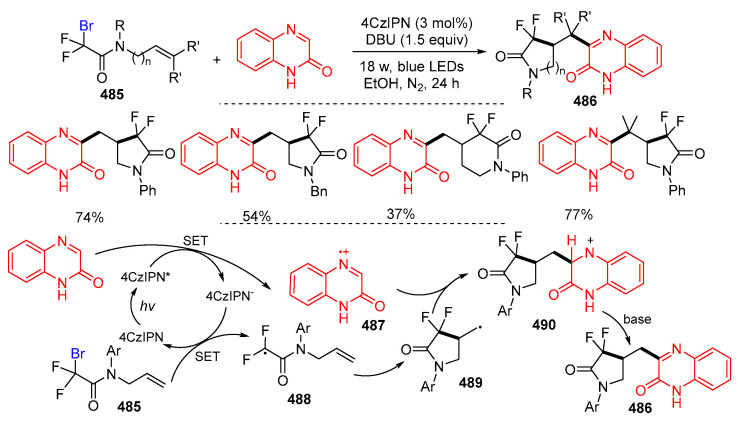
Synthesis of difluorolactam-attached quinoxalin-2(1*H*)-ones.

In 2022, Zhou, Sun and their coworkers reported a photo-promoted radical reaction of bromodifluoroacetamides with quinoxalin-2(1*H*)-ones for the synthesis of *α,α*-difluoro-*γ*-lactam-fused quinoxalin-2(1*H*)-ones (Scheme 97) [120]. The reaction of bromodifluoroacetamides **485**, quinoxalin-2(1*H*)-ones, 4CzIPN, and DBU in EtOH and under the irradiation of blue LEDs for 24 h gave *α,α*-difluoro-*γ*-lactam-fused quinoxalin-2(1*H*)-ones **486** in moderate to good yields. In this reaction, excited-state 4CzIPN^*^ reacts with quinoxalin-

2(1*H*)-one to form radical cation **487** and 4CzIPN^–^, and the latter reacts with bromodifluoroacetamides **485** to give difluoroalkyl radicals **488**. Radicals **489** generated from 5-*exo* cyclization of radicals **488** are attacked by **487** to give intermediates **490** and then products **486** after deprotonation with a base.

**Scheme 98:**
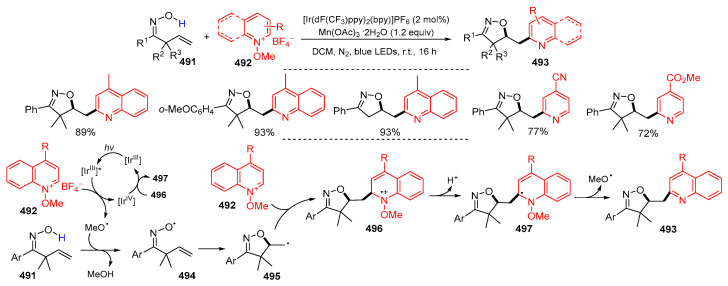
Synthesis of oxazoline-attached quinolines and pyridines.

He, Banwell and their coworkers, in 2023, introduced a visible light-mediated radical reaction of unsaturated oximes for the synthesis of methylene-bridged bis-heterocyclic compounds. The reaction of *β*,*γ*-unsaturated oximes **491**, *N*-methoxy quinolinium salts **492**, and Mn(OAc)_3_·2H_2_O with [Ir(dF(CF_3_)ppy)_2_(bpy)]PF_6_ as a photocatalyst under the irradiation of blue LEDs for 16 h gave dihydroisoxazoline-attached pyridines or quinolone **493** in good to excellent yields (Scheme 98) [121]. In the reaction process, methoxy radical generated from pyridinium salts **492** abstract an H atom from oximes **491** to form *O*-centered iminoxyl radical **494** which then undergoes 5-*exo* cyclization to form radical **495**. The reaction of radicals **495** and **492** gives radical cation **496** and then **497** after deprotonation. The demethylation via homolytic N–O bond cleavage affords oxazoline-attached quinolines **493** and regenerates the methoxy radical to complete the radical-chain process.

**Scheme 99:**
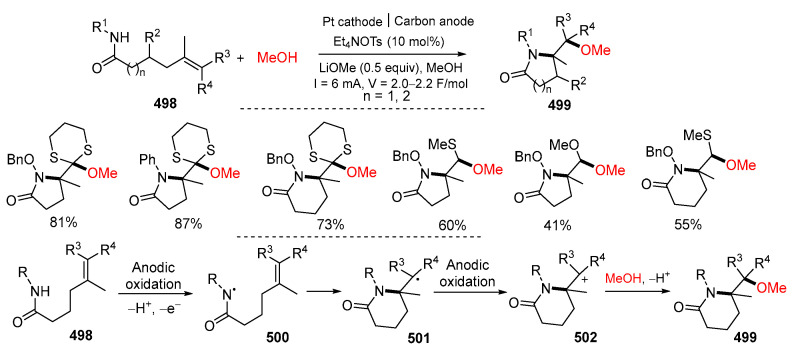
Synthesis of functionalized five- and six-membered lactams.

In 2014, Moeller and coworkers reported a radical reaction of *O*-benzyl hydroxamates or *N*-phenyl amides for the synthesis of five and six-membered lactams. In an electrolysis cell equipped with a reticulated vitreous carbon (RVC) anode and a platinum wire cathode, the reaction of electron-rich olefins such as *O*-benzyl hydroxamates or *N*-phenyl amides **498** in MeOH as a solution, Et_4_NOTs as the electrolyte, and LiOMe as a base, lactam products were obtained **499** in good yields (Scheme 99) [122]. In the synthesis of 6-membered lactams, amidyl radicals **500** generated anodically from *O*-benzyl hydroxamates **498** undergo a 6-*exo* cyclization to give radicals **501** which are oxidized to cations **502** followed by MeOH trapping to give final products **499**. 

**Scheme 100:**
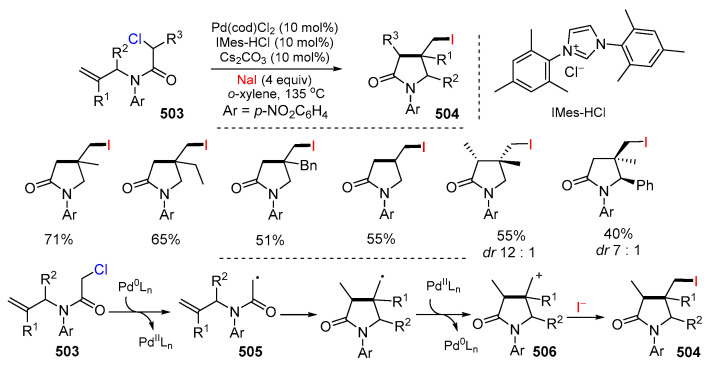
Synthesis iodosubstituted *γ*-lactams.

Tong and coworkers, in 2015, reported a Pd-catalyzed radical reaction of *N*-allyl-*α*-chloroamides for the synthesis of substituted *γ*-lactams. The reaction of *N*-allyl-*α*-chloroamides **503** in the presence of Pd(cod)Cl_2_ catalyst, *N*-heterocyclic carbene ligand (IMes), NaI additive, and Cs_2_CO_3_ base in *o*-xylene at 135 °C gave *β*-iodomethyl *γ*-lactam **504** in moderate to good yields and good stereoselectivity (Scheme 100) [123]. The proposed reaction mechanism suggests that radicals **505** produced from chloroamides **503** undergo 5-*exo* cyclization followed by single-electron oxidation with Pd^II^L_n_ to give cations **506.** Further reaction with NaI gives products **504**.

**Scheme 101:**
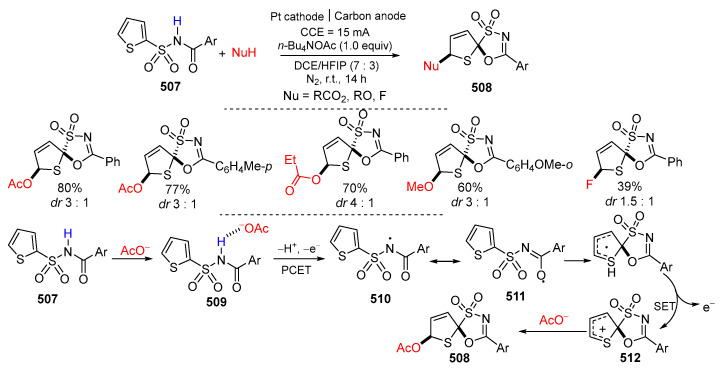
Synthesis of spirocycles.

An electrochemical dearomative spirocyclization reaction of *N*-acyl thiophene-2-sulfonamides for the synthesis of spirocycles was reported by Ye in 2022. The reaction of *N*-acyl thiophene-2-sulfonamides **507**, *n*-Bu_4_NOAc and HOAc in an undivided cell (carbon anode, Pt cathode) for 2.5 h gave spirocycles **508** in good yields (Scheme 101) [124]. It is a regio-specific radical spirocyclization of C2-tethered thiophenes. The complexes **509** produced from *N*-sulfonyl-benzamide **507** undergo electrochemical proton-coupled electron transfer (PCET) to yield amidyl radicals **510** which has a resonance structure of *O*-centered radicals **511**. The 5-*exo* spirocyclization of radicals **511** followed by oxidation to thiocarbenium ions **512** and then nucleophilic interception yields spirocycles **508**.

**Scheme 102:**
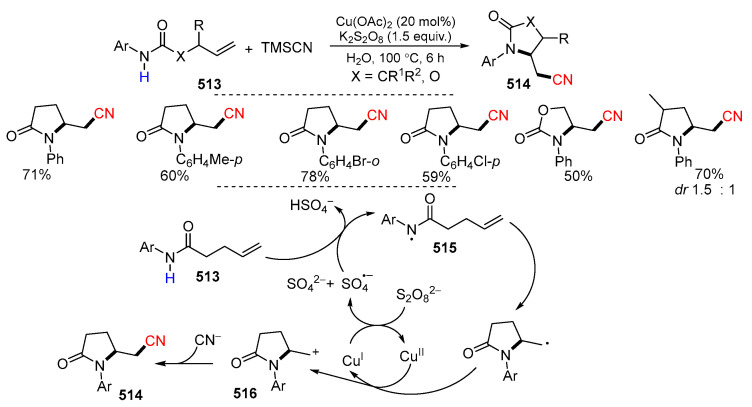
Synthesis of cyano-substituted *γ*-lactams.

In 2023, Li and Fang and coworkers introduced a Cu-catalyzed radical reaction of *N*-aryl-4-pentenamides for the synthesis of cyano-substituted *γ*-lactams. The reaction of *N*-aryl-4-pentenamides **513**, TMSCN, Cu(OAc)_2_ and K_2_S_2_O_8_ at 100 °C gave cyano-substituted *γ*-lactams **514** in moderate to good yields (Scheme 102) [125]. The proposed reaction mechanism suggested that amidyl radicals **515** generated from **513** via HAT with SO_4_ radical anion undergo 5-*exo* cyclization followed by oxidization with Cu^II^ to give cations **516**. Meanwhile, Cu^II^ is reproduced for the catalytic cycle with the aid of persulfate. Products **514** are obtained by the reaction of cations **516** and cyano anion.

**Scheme 103:**
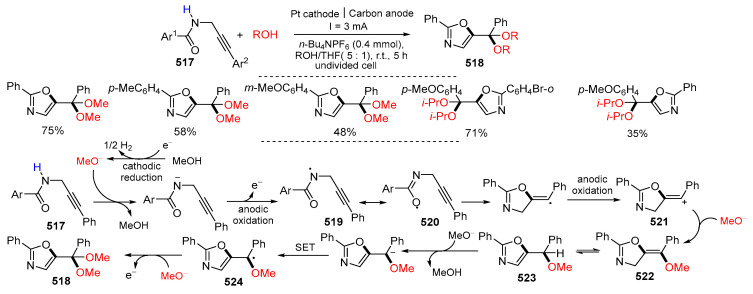
Radical cyclization for preparation of oxazole ketals.

An electrochemical reaction of *N*-propargylbenzamides for the synthesis of oxazole ketals was developed by the Xiao group in 2023. The reaction of *N*-propargylbenzamides **517** and *n*-Bu_4_NPF_6_ in an undivided cell with graphite rod anode and platinum plate cathode for 5 h produced oxazole ketals **518** in moderate to good yields (Scheme 103) [126]. In this reaction, anions generated from the deprotonation of **517** with MeO^–^ undergo anodic oxidation to give *N*-centered radicals **519** which have *O*-centered radicals **520** as the resonance structures. Cyclization of radicals **520** followed by anodic oxidation generates cations **521**, which then react with MeO^–^ to form **522** and rearrange to compounds **523**. Deprotonation of **523** with MeO^−^ followed by single-electron oxidation gives radicals **524** and then oxazole ketals **518** after anode oxidation and nucleophilic substitution with MeO^–^. 

## 7. Conclusions 

In this article, radical difunctionalization reactions are presented. These reactions are initiated by radical cyclization followed by a second functionalization. It is a new addition to our previous reviews on radical 1,2-difunctionalization, remote 1,3-, 1,4-, 1,5-, 1,6- and 1,7-difunctionalization, and addition followed by cyclization difunctionalization reactions. For the current topic, the initial cyclization could result in a wide range of cyclic and heterocyclic compounds with variable ring size, while the second functionalization decorates the ring by atom transfer, radical or transition metal coupling, reacting with neutral molecules or with cationic and anionic species. There are many options for the second functionalization to increase the diversity of the products. Future work on the cyclative difunctionalization reactions could be directed to the development of new substrates for generating the initial radicals for cyclization. It is also important to develop new processes and reactants for the second functionalization. The recent advances in photoredox reactions, electrochemical reactions, and transition metal-catalyzed coupling reactions provide ample opportunities for both the formation of initial radicals and for the second radical functionalization. The synthetically efficient and operationally simple radical cyclative difunctionalization reactions can be further explored and fully utilized in the synthesis of unique compounds with potential biological and functional material applications.

## Abbreviations

VC	ascorbic acid
ATRC	atom-transfer radical cyclization
BNPA	1,1-binaphthyl-2,2-diyl hydrogen-phosphate
TBHP	*tert*-butyl hydroperoxide
TBN	*tert*-butyl nitrite
DTBP	*di*-*tert*-butyl peroxide
NCS	*N*-chlorosuccinimide
DABSO	1,4-diazoniabicyclo[2.2.2]-octane-1,4-disulfinate
DLP	dilauroyl peroxide
DMA	dimethylacetamide
EDA	electron donor acceptor
NFSI	*N*-fluorobenzenesulfonimide
NHC	*N*-heterocyclic carbene
HFIP	hexafluoro-iso-propanol
DMDC	methyl dicarbonate
PMDETA	pentamethyldiethylenetriamine
1,10-Phen	1,10-phenanthroline
PCET	proton-coupled electron transfer
RVC	reticulated vitreous carbon
SET	single electron transfer
SED	super electron donor
4CzIPN	2,4,5,6-tetra(9*H*-carbazol-9-yl)isophthalonitrile
TMEDA	tetramethylethylenediamine
TEMPO	(2,2,6,6-tetramethylpiperidin-1-yl)-oxy
TCCA	trichloroisocyanuric acid
Me_6_TREN	tris [2-(dimethyl-amino)ethyl]amine
TPMA	tris(2-pyridylmethyl)amine

## References

[1] Chatgilialoglu C., Studer A. (2012). Encyclopedia of Radicals in Chemistry, Biology and Materials.

[2] Zarf S.Z. (2003). Radical Reactions in Organic Synthesis.

[3] Si Y.-F., Lv Q.-Y., Yu B. (2021). Radical cascade reactions of *β,γ*-unsaturated hydrazones/oximes. Adv. Synth. Catal..

[4] Coussanes G., Vila X., Diaba F., Bonjoch J. (2017). Radical cyclization of trichloroacetamides: Synthesis of lactams. Synthesis.

[5] Huang M.-H., Hao W.-J., Jiang B. (2018). Recent advances in radical-enabled bicyclization and annulation/1,n-bifunctionalization reactions. Chem. Asian J..

[6] Yan M., Kawamata Y., Baran P.S. (2017). Synthetic organic electrochemical methods since 2000: On the verge of a renaissance. Chem. Rev..

[7] Dong D.-Q., Tian B.-L., Yang H., Wei Z.-H., Yang S.-H., Zhou M.-Y., Ding C.-Z., Wang Y.-L., Gao J.-H., Wang S.-J. (2023). Visible light induced palladium-catalyzed reactions involving halogenated hydrocarbon (RX). Mol. Catal..

[8] Bag D., Koura H., Sawant S.D. (2020). Photo-induced 1,2-carbohalofunctionalization of C–C multiple bonds via ATRA pathway. Org. Biomol. Chem..

[9] Leitch J.A., Browne D.L. (2021). Mechanoredox chemistry as an emerging strategy in synthesis. Chem. Eur. J..

[10] Prier C.K., Rankic D.A., MacMillan D.W.C. (2013). Visible light photoredox catalysis with transition metal complexes: Applications in organic Synthesis. Chem. Rev..

[11] Ge Y., Tian Y., Wu J., Yan Q., Zheng L., Ren Y., Zhao J., Li Z. (2020). Iron-promoted free radical cascade difunctionalization of unsaturated benzamides with silanes. Chem. Commun..

[12] Bag D., Mahajan S., Sawant S.D. (2020). Transition-metal-catalyzed carbohalogenative 1,2-difunctionalization of C−C multiple bonds. Adv. Synth. Catal..

[13] Clark A.J. (2016). Copper catalyzed atom transfer radical cyclization reactions. Eur. J. Org. Chem..

[14] Liu Q., Dong X., Li J., Xiao J., Dong Y., Liu H. (2015). Recent advances on palladium radical involved reactions. ACS Catal..

[15] Wu Y.-C., Xiao Y.-T., Yang Y.-Z., Song R.-J., Li J.-H. (2020). Recent advances in silver-mediated radical difunctionalization of alkenes. ChemCatChem.

[16] Siu J.C., Fu N.K., Lin S. (2020). Catalyzing electrosynthesis: A homogeneous electrocatalytic approach to reaction discovery. Acc. Chem. Res..

[17] Li Z.L., Fang G.C., Gu Q.S., Liu X.Y. (2020). Recent advances in copper-catalysed radical-involved asymmetric 1,2-difunctionalization of alkenes. Chem. Soc. Rev..

[18] Jiang H., Studer A. (2020). Intermolecular radical carboamination of alkenes. Chem. Soc. Rev..

[19] Lan X.-W., Wang N.-X., Xing Y. (2017). Recent advances in radical difunctionalization of simple alkenes. Eur. J. Org. Chem..

[20] Bao X.Z., Li J., Jiang W., Huo C.D. (2019). Radical-mediated difunctionalization of styrenes. Synthesis.

[21] Lin J., Song R.J., Hu M., Li J.H. (2019). Recent advances in the intermolecular oxidative difunctionalization of alkenes. Chem. Rec..

[22] Gao Y., Tang G., Zhao Y.F. (2017). Recent progress toward organophosphorus compounds based on phosphorus-centered radical difunctionalizations. Phosphorus Sulfur Silicon Relat. Elem..

[23] Koike T., Akita M. (2016). A versatile strategy for difunctionalization of carbon–carbon multiple bonds by photoredox catalysis. Org. Chem. Front..

[24] Besset T., Poisson T., Pannecoucke X. (2015). Direct vicinal difunctionalization of alkynes: An efficient approach towards the synthesis of highly functionalized fluorinated alkenes. Eur. J. Org. Chem..

[25] Coppola G.A., Pillitteri S., Van der Eycken E.V., You S.-L., Sharma U.K. (2022). Multicomponent reactions and photo/electrochemistry join forces: Atom economy meets energy efficiency. Chem. Soc. Rev..

[26] Yao H., Hu W., Zhang W. (2021). Difunctionalization of alkenes and alkynes via intermolecular radical and nucleophilic additions. Molecules.

[27] Ma X., Zhang Q., Zhang W. (2023). Remote radical 1,3-, 1,4-, 1,5-, 1,6-and 1,7-difunctionalization reactions. Molecules.

[28] Zhi S., Yao H., Zhang W. (2023). Difunctionalization of dienes, enynes and related compounds via sequential radical addition and cyclization reactions. Molecules.

[29] Shen Y., Cornella J., Juliá-Hernández F., Martin R. (2017). Visible-light-promoted atom transfer radical cyclization of unactivated alkyl iodides. ACS Catal..

[30] Weng W.-Z., Liang H., Liu R.-Z., Ji Y.-X., Zhang B. (2019). Visible-light-promoted manganese-catalyzed atom transfer radical cyclization of unactivated alkyl iodides. Org. Lett..

[31] Zhang Y., Xu J., Guo H. (2019). Light-induced intramolecular iodine-atom transfer radical addition of alkyne: An approach from aryl iodide to alkenyl iodide. Org. Lett..

[32] Song L., Fang X., Wang Z., Liu K., Li C. (2016). Stereoselectivity of 6-*exo* cyclization of *α*-carbamoyl radicals. J. Org. Chem..

[33] Puccetti F., Schumacher C., Wotruba H., Hernández J.G., Bolm C. (2020). The use of copper and vanadium mineral ores in catalyzed mechanochemical carbon–carbon bond formations. ACS Sustain. Chem. Eng..

[34] Schumacher C., Hernández J.G., Bolm C. (2020). Electro-mechanochemical atom transfer radical cyclizations using piezoelectric BaTiO_3_. Angew. Chem. Int. Ed..

[35] Zhou Q., Chin M., Fu Y., Liu P., Yang Y. (2021). Stereodivergent atom-transfer radical cyclization by engineered cytochromes P450. Science.

[36] Tsuchiya N., Nakashima Y., Hirata G., Nishikata T. (2021). Atom-transfer radical cyclization of α-bromocarboxamides under organophotocatalytic conditions. Tetrahedron Lett..

[37] Clark A.J., Collis A.E.C., Fox D.J., Halliwell L.L., James N., O’Reilly R.K., Parekh H., Ross A., Sellars A.B., Willcock H. (2012). Atom-transfer cyclization with CuSO_4_/KBH_4_: A formal “activators generated by electron transfer” process also applicable to atom-transfer polymerization. J. Org. Chem..

[38] Lubskyy A., Guo C., Chadwick R.J., Petri-Fink A., Bruns N., Pellizzoni M.M. (2022). Engineered myoglobin as a catalyst for atom transfer radical cyclisation. Chem. Commun..

[39] Diaba F., Martínez-Laporta A., Bonjoch J. (2014). Chlorine atom transfer radical 6-*exo* cyclizations of carbamoyldichloroacetate-tethered alkenes, enol acetates and *α,β*-unsaturated nitriles leading to morphans. Eur. J. Org. Chem..

[40] Clark A.J., Cornia A., Felluga F., Gennaro A., Ghelfi F., Isse A.A., Menziani M.C., Muniz-Miranda F., Roncaglia F., Spinelli D. (2014). Arylsulfonyl groups: The best cyclization auxiliaries for the preparation of ATRC *γ*-lactams can be acidolytically removed. Eur. J. Org. Chem..

[41] Isse A.A., Visonà G., Ghelfi F., Roncaglia F., Gennaro A. (2015). Electrochemical approach to copper-catalyzed reversed atom transfer radical cyclization. Adv. Synth. Catal..

[42] Ram R.N., Soni V.K. (2016). Copper(I)-catalyzed synthesis of chlorinated tetrahydropyridazin-3-ones and 1,2-diazepan-3-ones from *N*-allyl-*N*′-trichloroacetylhydrazines. Adv. Synth. Catal..

[43] Ram R.N., Gupta D.K. (2016). Direct and stereoselective synthesis of diversely substituted 2,4-*trans*-(NH)-pyrrolidines by copper(I)-catalyzed radical cyclization at low temperature. Adv. Synth. Catal..

[44] Hou L., Zhou Z., Wang D., Zhang Y., Chen X., Zhou L., Hong Y., Liu W., Hou Y., Tong X. (2017). DPPF-catalyzed atom-transfer radical cyclization via allylic radical. Org. Lett..

[45] Sadanandan S., Gupta D.K. (2020). Changing stereoselectivity and regioselectivity in copper(I)-catalyzed 5-*exo* cyclization by chelation and rigidity in aminoalkyl radicals: Synthesis towards diverse bioactive N-heterocycles. New J. Chem..

[46] Ram R.N., Sadanandan S., Gupta D.K. (2019). *β,β,β*-Trichloroethyl-*N*H-enamine as viable system for 5-*endo*-*trig* radical cyclization via multifaceted Cu^I^−Cu^II^ redox catalysis: Single step synthesis of multi-functionalized *N*H-pyrroles. Adv. Synth. Catal..

[47] Yang X., Yu W. (2022). Promoting effect of water on light and phenanthroline-diphosphine Cu(I) complex-initiated iodine atom transfer cyclisation. Chem. Commun..

[48] Suzuki Y., Okita Y., Morita T., Yoshimi Y. (2014). An approach to the synthesis of naphtho[*b*]furans from allyl bromonaphthyl ethers employing sequential photoinduced radical cyclization and dehydrohalogenation reactions. Tetrahedron Lett..

[49] Ram R.N., Gupta D.K., Soni V.K. (2016). Copper(I)/ligand-catalyzed 5-*endo* radical cyclization-aromatization of 2,2,2-trichloroethyl vinyl ethers: Synthesis of 2,3-difunctionalized 4-chlorofurans. J. Org. Chem..

[50] Tittal R.K. (2018). Synthesis and molecular crystal of 3-chloro-2-(1-chloro-1-methyl-ethyl)-2,3-dihydro-1*H*-naphtho [2,1-*b*]oxepin-4-one. J. Mol. Struct..

[51] Roncaglia F., Ghelfi F., Felluga F., Poppi V. (2014). Simultaneous deprotection–oxidation of cyclic hemiacetals: A fine ending for a Ueno–Stork ATRC to dichloro-*γ*-lactones. Tetrahedron Lett..

[52] Ram R.N., Gupta D.K., Soni V.K. (2016). Copper(I)-promoted synthesis of highly substituted and functionalized tetrahydrothiophenes. Eur. J. Org. Chem..

[53] Ram R.N., Kumar N., Gupta D.K. (2017). Substrate-controlled diastereoselective synthesis of sugar-based chlorinated perhydrofuro [2,3-*b*]pyrans via copper(I)-catalyzed radical cyclization. Adv. Synth. Catal..

[54] Ratushnyy M., Parasram M., Wang Y., Gevorgyan V. (2018). Palladium-catalyzed atom-transfer radical cyclization at remote unactivated C(sp^3^)−H sites: Hydrogen-atom transfer of hybrid vinyl palladium radical intermediates. Angew. Chem. Int. Ed..

[55] Kim Y., Lee K., Mathi G.R., Kima I., Hong S. (2019). Visible-light-induced cascade radical ring-closure and pyridylation for the synthesis of tetrahydrofurans. Green Chem..

[56] Zidan M., McCallum T., Swann R., Barriault L. (2020). Formal bromine atom transfer radical addition of nonactivated bromoalkanes using photoredox gold catalysis. Org. Lett..

[57] Dawra N., Ram R.N., Thakur S.K. (2020). Synthesis of chlorinated, heteroatom-rich and differently fused tricyclic *β*-lactams by Cu(I)-catalyzed halogen atom transfer radical cyclization. Chem. Sel..

[58] Wei H.-Z., Wei Y., Shi M. (2021). Intramolecular difunctionalization of methylenecyclopropanes tethered with carboxylic acid by visible-light photoredox catalysis. Org. Chem. Front..

[59] Klychnikov M.K., Pohl R., Císařová I., Jahn U. (2021). *α,γ*-Dioxygenated amides via tandem Brook rearrangement / radical oxygenation reactions and their application to syntheses of *γ*-lactams. Beilstein J. Org. Chem..

[60] Marchese A.D., Durant A.G., Reid C.M., Jans C., Arora R., Lautens M. (2022). Pd(0)/blue light promoted carboiodination reaction-evidence for reversible C–I bond formation via a radical pathway. J. Am. Chem. Soc..

[61] Zhou T., Chen H., Liu Y., Wang H., Yan Q., Wang W., Chen F. (2022). Visible-light-promoted xanthate-transfer cyclization reactions of unactivated olefins under photocatalyst- and additive-free conditions. J. Org. Chem..

[62] Yang X., Zhou J., Wu S., Yu W. (2023). Copper-mediated bromine atom transfer radical cyclisation of unactivated alkyl bromides. Chem. Commun..

[63] Peng X.-X., Deng Y.-J., Yang X.-L., Zhang L., Yu W., Han B. (2014). Iminoxyl radical-promoted dichotomous cyclizations: Efficient oxyoximation and aminooximation of alkenes. Org. Lett..

[64] Wang D.-J., Chen B.-Y., Wang Y.-Q., Zhang X.-W. (2018). Ruthenium-catalyzed direct transformation of alkenyl oximes to 5-cyanated isoxazolines: A cascade approach based on non-stabilized radical intermediate. Eur. J. Org. Chem..

[65] Zhang X.-W., Xiao Z.-F., Wang M.-M., Zhuang Y.-J., Kang Y.-B. (2016). Transition-metal-free oxychlorination of alkenyl oximes: In situ generated radicals with *tert*-butyl nitrite. Org. Biomol. Chem..

[66] Zhang W., Su Y., Wang K.-H., Wu L., Chang B., Shi Y., Huang D., Hu Y. (2017). Trichloroisocyanuric acid promoted cascade cyclization/trifluoromethylation of allylic oximes: Synthesis of trifluoromethylated ioxazolines. Org. Lett..

[67] Li X., Ding Y., Qian L., Gao Y., Wang X., Yan X., Xu X. (2019). General 5-halomethyl isoxazoline synthesis enabled by copper-catalyzed oxyhalogenation of alkenes. J. Org. Chem..

[68] Paderes M.C., Keister J.B., Chemler S.R. (2013). Mechanistic analysis and optimization of the copper-catalyzed enantioselective intramolecular alkene aminooxygenation. J. Org. Chem..

[69] Folgueiras-Amador A.A., Philipps K., Guilbaud S., Poelakker J., Wirth T. (2017). An easy-to-machine electrochemical flow microreactor: Efficient synthesis of isoindolinone and flow functionalization. Angew. Chem. Int. Ed..

[70] Azzi E., Ghigo G., Sarasino L., Parisotto S., Moro R., Renzi P., Deagostino A. (2023). Photoinduced chloroamination cyclization cascade with *N*-chlorosuccinimide: From *N*-(allenyl)sulfonylamides to 2-(1-chlorovinyl)pyrrolidines. J. Org. Chem..

[71] Ma J.-W., Chen X., Zhou Z.-Z., Liang Y.-M. (2020). Visible-light-induced palladium-catalyzed carbocyclization of unactivated alkyl bromides with alkenes involving C–I or C–B coupling. J. Org. Chem..

[72] Yin H., Huang T., Shi B., Cao W., Yu C., Li T., Zhang K., Yao C. (2023). Synthesis of 2-pyrrolidinone derivatives via *N*-heterocyclic carbene catalyzed radical tandem cyclization/coupling reactions. Org. Chem. Front..

[73] Dong X., Hu Y., Xiao T., Zhou L. (2015). Synthesis of 2-trifluoromethyl indoles via visible-light induced intramolecular radical cyclization. RSC Adv..

[74] Zhou S., Yuan F., Guo M., Wang G., Tang X., Zhao W. (2018). Switchable synthetic strategy toward trisubstituted and tetrasubstituted exocyclic alkenes. Org. Lett..

[75] Zhang L., Si X., Rominger F., Hashmi A.S.K. (2020). Visible-light-induced radical carbo-cyclization/*gem*-diborylation through triplet energy transfer between a gold catalyst and aryl iodides. J. Am. Chem. Soc..

[76] Xu J., Ding A., Zhang Y., Guo H. (2021). Photochemical synthesis of 1,4-dicarbonyl bifluorene compounds via oxidative radical coupling using TEMPO as the oxygen atom donor. J. Org. Chem..

[77] Deng G., Li M., Yu K., Liu C., Liu Z., Duan S., Chen W., Yang X., Zhang H., Walsh P.J. (2019). Synthesis of benzofuran derivatives through cascade radical cyclization/intermolecular coupling of 2-azaallyls. Angew. Chem. Int. Ed..

[78] Yu K., Li M., Deng G., Liu C., Wang J., Liu Z., Zhang H., Yang X., Walsh P.J. (2019). An efficient route to isochromene derivatives via cascade radical cyclization and radical-radical coupling. Adv. Synth. Catal..

[79] Kakaeia S., Xu J. (2013). Synthesis of (2-alkylthiothiazolin-5-yl)methyl dodecanoates via tandem radical reaction. Org. Biomol. Chem..

[80] Quertenmont M., Goodall I., Lam K., Markó I., Riant O. (2020). Kolbe anodic decarboxylation as a green way to access 2-pyrrolidinones. Org. Lett..

[81] Quertenmont M., Toussaint F.C., Defrance T., Lam K., Markó I.E., Riant O. (2021). Continuous flow electrochemical oxidative cyclization and successive functionalization of 2-pyrrolidinones. Org. Process Res. Dev..

[82] Singh J., Nelson T.J., Mansfield S.A., Nickel G.A., Cai Y., Jones D.D., Small J.E., Ess D.H., Castle S.L. (2022). Microwave- and thermally promoted iminyl radical cyclizations: A versatile method for the synthesis of functionalized pyrrolines. J. Org. Chem..

[83] Burde A.S., Chemler S.R. (2022). Copper-catalyzed enantioselective oxysulfenylation of alkenols: Synthesis of arylthiomethyl-substituted cyclic ethers. ACS Catal..

[84] Ke S., Liao H., Qin H., Wang Y., Li Y. (2023). Access to benzocyclic boronates via light-promoted intramolecular arylborylation of alkenes. J. Org. Chem..

[85] Dong Y.-X., Zhang C.-L., Gao Z.-H., Ye S. (2023). Iminoacylation of alkenes via photoredox *N*-heterocyclic carbene catalysis. Org. Lett..

[86] Ward R.M., Schomaker J.M. (2021). Allene trifunctionalization via amidyl radical cyclization and TEMPO trapping. J. Org. Chem..

[87] Khoder Z.M., Wong C.E., Chemler S.R. (2017). Stereoselective synthesis of isoxazolidines via copper-catalyzed alkene diamination. ACS Catal..

[88] Chen F., Zhu F.-F., Zhang M., Liu R.-H., Yu W., Han B. (2017). Iminoxyl radical-promoted oxycyanation and aminocyanation of unactivated alkenes: Synthesis of cyano-featured isoxazolines and cyclic nitrones. Org. Lett..

[89] Wang L.-J., Ren P.-X., Qi L., Chen M., Lu Y.-L., Zhao J.-Y., Liu R., Chen J.-M., Li W. (2018). Copper-mediated aminoazidation, aminohalogenation, and aminothiocyanation of *β,γ*-unsaturated hydrazones: Synthesis of versatile functionalized pyrazolines. Org. Lett..

[90] Chen C., Ding J., Wang Y., Shi X., Jiao D., Zhu B. (2019). Copper-catalyzed iminohalogenation of *γ, δ*-unsaturated oxime esters with halide salts: Synthesis of 2-halomethyl pyrrolines. Tetrahedron Lett..

[91] Sun Q., Yoshikai N. (2019). Cobalt-catalyzed tandem radical cyclization/C–C coupling initiated by directed C–H activation. Org. Lett..

[92] Song X.-D., Li X.-R., Wang Y.-W., Chu X.-Q., Rao W., Xu H., Han G.-Z., Shen Z.-L. (2020). Indium-mediated difunctionalization of iodoalkyl-tethered unactivated alkenes via an intramolecular cyclization and an ensuing palladium-catalyzed cross-coupling reaction with aryl halides. Org. Chem. Front..

[93] Meng F., Zhang H., He H., Xu N., Fang Q., Guo K., Cao S., Shi Y., Zhu Y. (2020). Copper-catalyzed domino cyclization/thiocyanation of unactivated olefins: Access to SCN-containing pyrazolines. Adv. Synth. Catal..

[94] Chen J., Zhu Y.-P., Li J.-H., Wang Q.-A. (2021). External-oxidant-free amino-benzoyloxylation of unactivated alkenes of unsaturated ketoximes with *O*-benzoylhydroxylamines. Chem. Commun..

[95] Zhang Z., Ngo D.T., Nagib D.A. (2021). Regioselective radical amino-functionalizations of allyl alcohols via dual catalytic cross-coupling. ACS Catal..

[96] Chen S., Meervelt L.V., Van der Eycken E.V., Sharma U.K. (2022). Visible-light-driven palladium-catalyzed radical tandem dearomatization of indoles with unactivated alkenes. Org. Lett..

[97] Nieto-Carmona J.C., Román R.S., Buñuel E., Cárdenas D.J. (2022). Nickel-catalyzed cascade cyclization-Negishi coupling of redox active esters for the synthesis of pyrrolidines. Eur. J. Org. Chem..

[98] Hwang J.Y., Baek J.H., Shin T.I., Shin J.H., Oh J.W., Kim K.P., You Y., Kang E.J. (2016). Single-electron-transfer strategy for reductive radical cyclization: Fe(CO)_5_ and phenanthroline system. Org. Lett..

[99] Chen D., Chemler S.R. (2018). Synthesis of phthalans via copper-catalyzed enantioselective cyclization/carboetherification of 2-vinylbenzyl alcohols. Org. Lett..

[100] Koy M., Bellotti P., Katzenburg F., Daniliuc C.G., Glorius F. (2020). Synthesis of all-carbon quaternary centers by palladium-catalyzed olefin dicarbofunctionalization. Angew. Chem. Int. Ed..

[101] Smoleń S., Wincenciuk A., Drapała O., Gryko D. (2021). Vitamin B_12_-catalyzed dicarbofunctionalization of bromoalkenes under visible light irradiation. Synthesis.

[102] Li P.-S., Teng Q.-Q., Chen M. (2023). Photoinduced radical cascade domino Heck coupling of *N*-aryl acrylamide with vinyl arenes enabled by palladium catalysis. Chem. Commun..

[103] Duan X.-Y., Yang X.-L., Jia P.-P., Zhang M., Han B. (2015). Hydrazonyl radical-participated tandem reaction: A strategy for the synthesis of pyrazoline-functionalized oxindoles. Org. Lett..

[104] Yang X.-L., Long Y., Chen F., Han B. (2016). Synthesis of isoxazoline-featured oxindoles by iminoxyl radical-promoted cascade oxyalkylation/alkylarylation of alkenes. Org. Chem. Front..

[105] Han W.-J., Wang Y.-R., Zhang J.-W., Chen F., Zhou B., Han B. (2018). Cu-catalyzed oxyalkynylation and aminoalkynylation of unactivated alkenes: Synthesis of alkynyl-featured isoxazolines and cyclic nitrones. Org. Lett..

[106] Fang Z., Xie L., Wang L., Zhang Q., Li D. (2022). Silver-catalyzed cascade cyclization and functionalization of *N*-aryl-4-pentenamides: An efficient route to *γ*-lactam-substituted quinone derivatives. RSC Adv..

[107] Zhao W., Xuan L., Liu Y., Yin J., Wang H., Yan Q., Wang W., Huang J., Chen F. (2023). Photoinduced tandem radical cyclization/heteroarylation of *N*-allylbromodifluoroacetamides with quinoxalin-2(1*H*)-ones or coumarins under metal- and photocatalyst-free conditions. New J. Chem..

[108] Peng X.-X., Wei D., Han W.-J., Chen F., Yu W., Han B. (2017). Dioxygen activation via Cu-catalyzed cascade radical reaction: An approach to isoxazoline/cyclic nitrone-featured *α*-ketols. ACS Catal..

[109] Liu X.-D., Wang Q.-A., Zhu Y.-P., Peng Z.-H., Li J.-H. (2022). Copper-catalyzed aerobic hydroxyamination of alkenes of unsaturated keto oximes in EtOH toward cyclic nitrones. Green Chem..

[110] Fu X.-Y., Si Y.-F., Qiao L.-P., Zhao Y.-F., Chen X.-L., Yu B. (2022). Visible light-promoted recyclable carbon nitride-catalyzed dioxygenation of *β,γ*-unsaturated oximes. Adv. Synth. Catal..

[111] Sun W., Cui X., Qu J., Cai X., Hu J., Xiong Z., Guo S., Xu J., Chen W.-H., Wu J.-Q. (2023). Facile access to 2-hydroxy-2-substituted indole-3-ones via a copper-catalyzed oxidative cyclization of 2-arylethynylanilines. Chem. Commun..

[112] Qi Z., Wen S., Liu Z., Jiang D. (2023). Oxygen-promoted 6-*endo*-trig cyclization of *β,γ*-unsaturated hydrazones/ketoximes with diazonium tetrafluoroborates for pyridazin-4(1*H*)-ones/oxazin-4(1*H*)-ones. Org. Lett..

[113] Ye H., Zhou L., Chen Y., Tong H. (2023). Visible light driven multicomponent synthesis of difluoroamidosulfonyl quinoline derivatives. Org. Biomol. Chem..

[114] Zhong T., Yi J.-T., Chen Z.-D., Zhuang Q.-C., Li Y.-Z., Lu G., Weng J. (2021). Photoredox-catalyzed aminofluorosulfonylation of unactivated olefins. Chem. Sci..

[115] Yan Z.-M., Qi L., Cao T.-Y., Liu J.-L., Du H.-J., Dong Y.-C., Li W., Wang L.-J. (2023). Aminofluorosulfonylation of *β,γ*-unsaturated hydrazones with sulfur dioxide and *N*-fluorobenzenesulfonimide: Accessing pyrazoline-functionalized aliphatic sulfonyl fluorides. Org. Lett..

[116] Yang X.-L., Chen F., Zhou N.-N., Yu W., Han B. (2014). Synthesis of isoxazoline-functionalized phenanthridines via iminoxyl radical-participated cascade sequence. Org. Lett..

[117] Micic N., Polyzos A. (2018). Radical carbonylation mediated by continuous-flow visible-light photocatalysis: Access to 2,3-dihydrobenzofurans. Org. Lett..

[118] Zheng D., Jana K., Alasmary F.A., Daniliuc C.G., Studer A. (2021). Transition-metal-free intramolecular radical aminoboration of unactivated alkenes. Org. Lett..

[119] Yu F., Yang S., Xie Z., Lu D., Gong Y. (2023). An iron-catalysed radical cyclization/C(sp^3^)–H alkylation cascade between *γ,δ*-unsaturated oxime esters and glycine derivatives: Access to pyrroline-containing amino acids. Org. Chem. Front..

[120] Zhang Y.-C., Chen Y., Sun J., Wang J.-Y., Zhou M.-D. (2022). Visible-light-promoted radical cyclization/arylation cascade for the construction of *α,α*-difluoro-*γ*-lactam-fused quinoxalin-2(1*H*)-ones. Chin. J. Chem..

[121] Yuan T.-T., Chen J., Pham L.N., Paul S., White L.V., Li J., Lan P., Coote M.L., Banwell M.G., He Y.-T. (2023). Visible light-mediated syntheses of unsymmetrical methylene-bridged bis-heterocycles via an alkoxy radical relay reaction. Org. Chem. Front..

[122] Xu H.-C., Campbell J.M., Moelle K.D. (2014). Cyclization reactions of anode-generated amidyl radicals. J. Org. Chem..

[123] Liu Q., Chen C., Tong X. (2015). Pd(0)-catalyzed atom transfer radical cyclization of *N*-allyl-*α*-chloroamides: Highly stereoselective synthesis of substituted *γ*-lactam. Tetrahedron Lett..

[124] Shi Z., Wang W.-Z., Li N., Yuan Y., Ye K.-Y. (2022). Electrochemical dearomative spirocyclization of *N*-acyl thiophene-2-sulfonamides. Org. Lett..

[125] Liu W., Wang L., Mu H., Zhang Q., Fang Z., Li D. (2023). Synthesis of cyano-substituted *γ*-lactams through a copper-catalyzed cascade cyclization/cyanation reaction. Org. Biomol. Chem..

[126] Zhang H., Xiong Y., Luo M.-J., Yang R., Bai J., Song X.-R., Xiao Q. (2023). Metal-free electrochemical oxidative intramolecular cyclization of *N*-propargylbenzamides: Facile access to oxazole ketals. Org. Chem. Front..

